# Synthesis and Biological Evaluation of S-, O- and Se-Containing Dispirooxindoles

**DOI:** 10.3390/molecules26247645

**Published:** 2021-12-16

**Authors:** Maksim Kukushkin, Vladimir Novotortsev, Vadim Filatov, Yan Ivanenkov, Dmitry Skvortsov, Mark Veselov, Radik Shafikov, Anna Moiseeva, Nikolay Zyk, Alexander Majouga, Elena Beloglazkina

**Affiliations:** 1 Department of Chemistry, Lomonosov Moscow State University, Leninskie Gory, 1/3, GSP-1, 119991 Moscow, Russia; lemmingg@mail.ru (M.K.); vladnov9216@rambler.ru (V.N.); nanovf@mail.ru (V.F.); skvorratd@mail.ru (D.S.); iltarn@mail.ru (R.S.); moiseeva@org.chem.msu.ru (A.M.); zyk@org.chem.msu.ru (N.Z.); alexander.majouga@gmail.com (A.M.); 2 Research Laboratory of Biophysics, National University of Science and Technology MISiS, 119049 Moscow, Russia; 3 Laboratory of Medicinal Chemistry and Bioinformatic, Moscow Institute of Physics and Technology (MIPT), Institutski Pereulok 9, 141701 Dolgoprudny, Russia; smart_people@inbox.ru (Y.I.); veselovmark@gmail.com (M.V.); 4 Dmitry Mendeleev University of Chemical Technology of Russia, Miusskaya Sq. 9, 125047 Moscow, Russia

**Keywords:** dispirooxindoles, anticancer activity, cytotoxicity, 3D molecular docking, p53/MDM2 interaction

## Abstract

A series of novel S-, O- and Se-containing dispirooxindole derivatives has been synthesized using 1,3-dipolar cycloaddition reaction of azomethine ylide generated from isatines and sarcosine at the double C=C bond of 5-indolidene-2-chalcogen-imidazolones (chalcogen was oxygen, sulfur or selenium). The cytotoxicity of these dispiro derivatives was evaluated in vitro using different tumor cell lines. Several molecules have demonstrated a considerable cytotoxicity against the panel and showed good selectivity towards colorectal carcinoma HCT116 p53^+/+^ over HCT116 p53^−/−^ cells. In particular, good results have been obtained for LNCaP prostate cell line. The performed in silico study has revealed MDM2/p53 interaction as one of the possible targets for the synthesized molecules. However, in contrast to selectivity revealed during the cell-based evaluation and the results obtained in computational study, no significant p53 activation using a reporter construction in p53wt A549 cell line was observed in a relevant concentration range.

## 1. Introduction

Design and development of novel potent anticancer therapeutics are the most important tasks of synthetic organic and medicinal chemistry. Among the compounds with antitumor action, an important place is occupied by the spiro and dispiro derivatives of indolinones, due to the conformational rigidity of spiro scaffold which allows the introduction into the molecules of functional groups necessary for interaction with biological targets in the required arrangement, and the indolinone fragment simulates the tryptophan moiety, in many cases involved in such interactions [1,2,3,4,5]. So, spiro-oxindole alkaloids, which were firstly derived from the families *Apocynaceae* and *Rubiaceae* [6] and latter were found in a wide range of complex natural products [7,8,9,10] have shown significant anticancer activity. These compounds contain the spiro ring fusion at position 3 of the indolinone core, with different substitutions around the pyrrolidine and indolinone moieties.

A promising direction in the treatment of cancer is the development of compounds that affect the interaction of p53–MDM2 proteins. The p53 protein, which is a tumor suppressor, is one of the potential targets of antitumor therapy. Tumor suppressor p53, not being complexed with its MDM2 inhibitor, can trigger cell apoptosis [11,12]. In more than 50% of tumor cell cultures, the p53 protein is mutated [12], and its activation or restoration of its function may be effective in anticancer therapy due to apoptosis initiating or arresting cell growth [13]. Note that some small molecules inhibit MDM2/p53 interaction and now are undergoing preclinical or clinical trials against different types of cancer [14,15,16,17,18]. Among these molecules, compound nutlin-3a is one of the most known inhibitors of the p53–MDM2 protein–protein interaction; this compound is able to bind to the p53–MDM2 pocket and inhibit this protein interaction in nanomolar concentrations [19]. Nutlin-3a induced nongenotoxic stabilization of p53 protein and subsequent activation of a p53 pathway [20]. The molecule of nutlin-3a, which definitely binds to the site 1 of MDM2 protein, may be a template for the design of new p53–MDM2 inhibitor molecules [21]. Since the indole ring of Trp23 residue of p53 is located deep inside a hydrophobic pocket of MDM2 and its NH group forms a hydrogen bond with the backbone carbonyl in MDM2, Trp23 appears to be most crucial for binding of p53 to MDM2. Previously [22], Wang’s group searched for chemical moieties that can mimic the Trp23 interaction with MDM2. In addition to the indole ring itself, they have found that oxindole can perfectly mimic the side chain of Trp23 for interaction with MDM2. These modeling studies also showed that compounds with a spiro-linked structure are capable of better binding to MDM2 by limiting the conformational mobility of the molecule (пpeдыдyщaя) and the spiro-(oxindole-3,3′-pyrrolidine) core structure may be used as the starting point for the design of a new class of MDM2 inhibitors. The oxindole can closely mimic the Trp23 side chain in p53 in both hydrogen bond formation and hydrophobic interactions with MDM2, and the spiro-pyrrolidine ring provides a rigid scaffold.

We have recently described a series of novel spiro-oxindoles containing thiohydantoin [23,24,25], selenohydantoin [26] or hydantoin [25,27] moieties, presumably having an anticancer effect by inhibiting the p53/MDM2 protein interaction; one such derivative has recently successfully completed preclinical trials as a drug for the treatment of colorectal cancer [28]. The most active compounds of this type have shown cytotoxicity in the 4–11 µm range on cancer cell lines HepG2, MCF-7, SiHa and HCT116, [23], and some p53 activation by Western blotting (see Supplementary Information, Figure S1).

**Figure 1:**
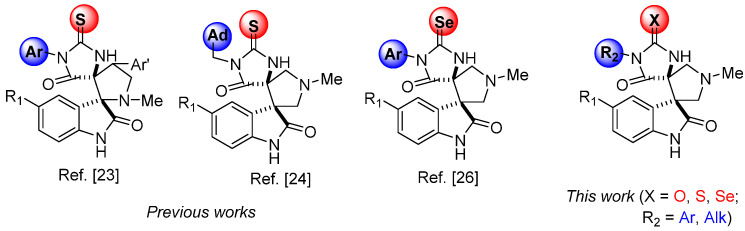
Compounds synthesized earlier and in this work.

In this paper, we present a series of novel compounds of the dispiro-indolinone series with a modified spiro-oxindole core (Figure 1) with promising anticancer activity. Most of the previously investigated thiohydantoin-based spiro-oxindoles [23,25,28] (Figure 1) had in their structures the nitrogen atom of the central pyrrolidine ring directly attached to the carbon atom at spiro-conjugation; in the series of compounds described in this article, nitrogen atom of pyrrolidine ring is in the central position of spiro-conjugated cycle, similar to the MI series compounds, which demonstrated a significant cytotoxic effect on the prostate cancer cell line LNCap, with IC_50_ = 86 nM, and on the colorectal cancer cell line HCTwt, with IC_50_ = 22 μM, and were recognized as a selective inhibitor of p53–MDM2 interaction due to theirability to induce cell apoptosis in tumor cells without affecting healthy ones [29].

In contrast to the compounds of such structural type, described in [24,26], this article presents dispiro derivatives with aryl and non-carcass alkyl substituents at N(3) position and with different exocyclic chalcogen atoms (oxygen, sulfur or selenium) in imidazolone fragment. Some results of cytotoxic action mechanisms studying for synthesized compounds are also presented, as well as the molecular docking data to evaluate their possible binding affinity toward MDM2.

## 2. Resultsand Discussion

### 2.1. Chemistry

**Scheme 1:**
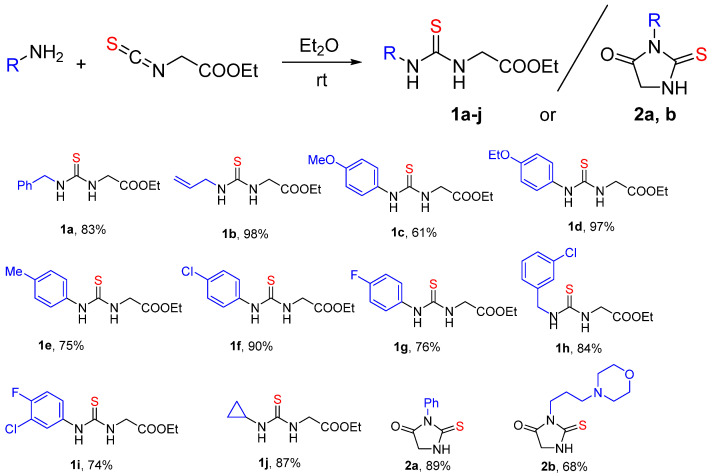
Synthesis of the compounds **1a**–**k**, **2**.

To obtain the target dispyro derivatives **4**, **6**, **9**, the series of disubstituted thioureas **1a**–**j** or thiohydantoins **2a**, **b** (Scheme 1) was initially synthesized starting from aryl- or alkyl-amine and ethyl isothiocyanatoacetate. The reaction proceeded smoothly in ether at room temperature and furnished the desired intermediates with 61–98% yield. 

**Scheme 2:**
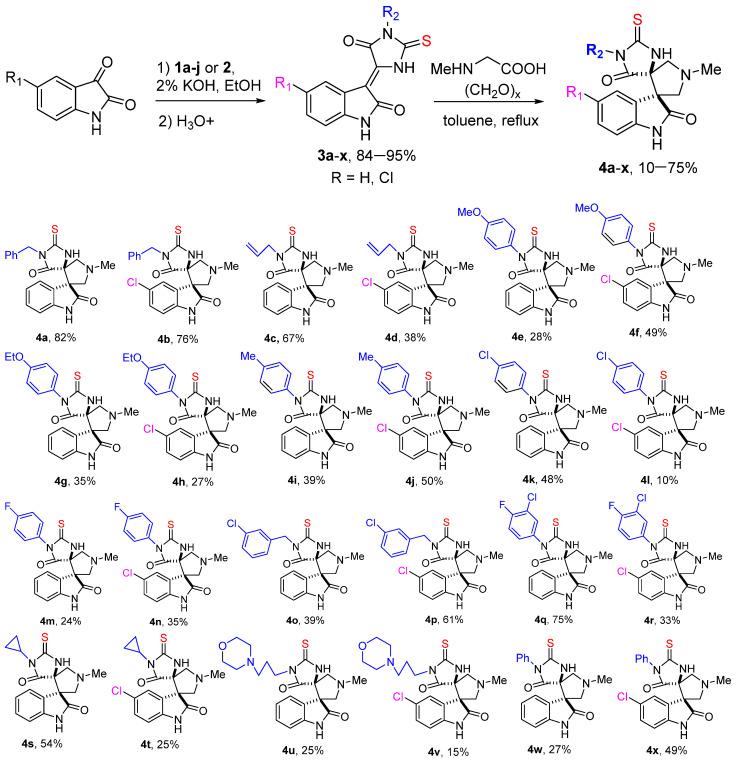
Synthesis of 3-(5-oxo-2-thioxoimidazolidin-4-ylidene)indolin-2-ones **3a**–**x** and dispiroindolinines **4a**–**x**.

**Figure 2:**
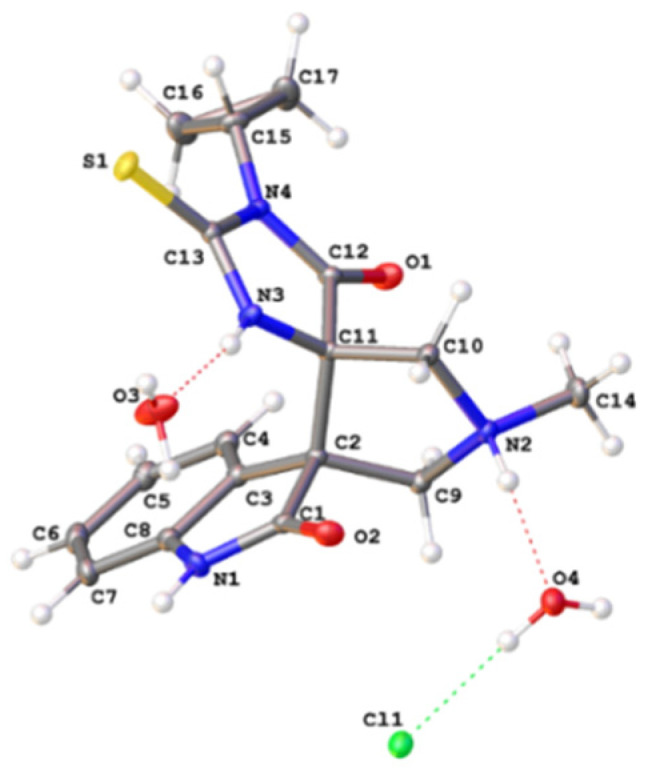
Molecular structure of compound **4s** (as a salt with HCl).

Compounds **1a**–**j** and **2a**, **b** were then treated with equimolar amount of isatin or 5-chloroisatin to obtain indolidene-thiohydantoins **3a**–**x** (Scheme 2), analogously to previously described reactions of substituted thioureas with aromatic aldehydes [30]. Finally, compounds **3a**–**x** were reacted with sarcosine and paraformaldehyde in toluene under reflux to obtain the desired substituted dispiroindolinones **4a**–**x** in a moderate-to-high yield. The reaction, apparently, proceeds according to the mechanism of 1,3-dipolar cycloaddition of azomethine ylide generated from isatin and sarcosine at the C=C double bond of indolidenehydantoins **3** [23]. According to the-NMR spectroscopy data, the reactions in all cases proceed with the formation of single diastereomeric products **4a**–**x** with the relative S*, R*-configuration, which was confirmed by the data of X-ray crystallographic analysis for the compound **4s** (Figure 2).

For comparison, we have synthesized some O- and Se-containing analogs of the spiro-hydantoins **4**, namely for compounds **4g**, **4h**, **4q**, **4r**. Some hydantoin derivatives containing spiro-linked indolinone fragments showed significant in vitro cytotoxic activity [25]. The ability of organoselenium compounds to exhibit antioxidant properties [31,32] mimicking the action of the glutathione peroxidase enzyme [33] makes it possible to use them in anticancer therapy as auxiliary antioxidants to neutralize the oxidizing agents produced by certain anticancer drugs.

**Scheme 3:**
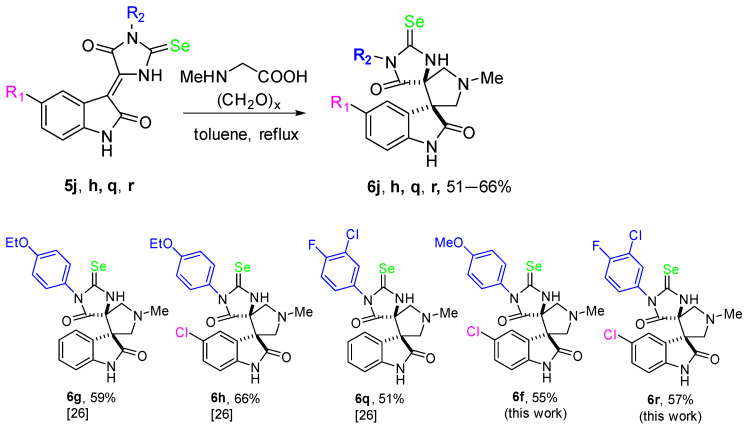
Synthesis of Se-containing analogue of the compounds **4**.

Selenohydantoin derivatives **6g**, **6h**, **6q**, **6r** may be readily obtained from sarcosine, paraformaldehyde and the corresponding indolidene-selenohydantoins **5g**, **5h**, **5q**, **5r** according to the modified method described in [26,34] (Scheme 3). 

**Scheme 4:**
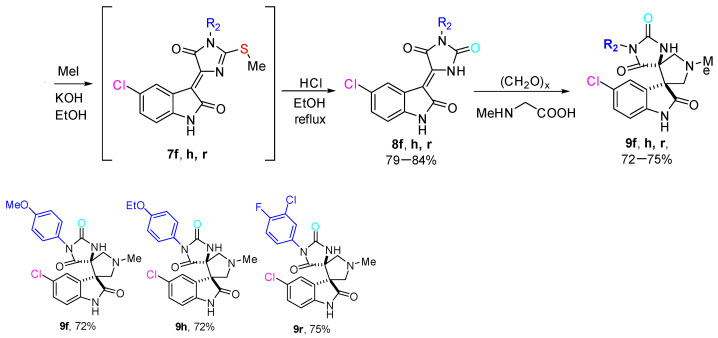
Synthesis of O-containing analogue of the compounds **4**.

O-containing derivatives **9** were synthesized from S-alkylated derivatives **7** of corresponding thiohydantoins **3** (Scheme 4). Thus, the starting compounds **3f**, **3h**, **3r** were vigorously stirred with MeI in KOH/EtOH at room temperature for 30 min and then were treated with HCl/EtOH under reflux conditions to provide the corresponding hydantoins **8** in good yield (about 70%). The desired products **9f**, **9h**, **9r** were obtained at the compounds **8** interaction with sarcosine and paraformaldehyde in 75% yields.

### 2.2. Biological Evaluation

#### 2.2.1. Cytotoxicity

All the synthesized dispiro-oxindoles **4** and some of their selenium and oxygen analogues **6**, **9** have subsequently been tested on their in vitro anticancer efficiency against a panel of different tumor cell lines, on the assumption that they, like the previously described compounds of dispiro-thiohydantoins type [23], may be able to inhibit p53/MDM protein interaction. The used models included human prostate cancer cell lines LNCaP and PC3, breast cancer cell line (MCF-7), human colon cancer cells (HCT116^+/+^, p53 positive, p53^+/+^ and HCT116^−/−^, p53 negative, p53^−/−^), human lung adenocarcinoma epithelial cell line (A549), SV40-transformed normal human lung fibroblast cells (VA13), as well as human embryonic kidney 293 cells (Hek293) stably expressing SV40 large T antigen (Hek293T). The cytotoxicity of the evaluated molecules was properly assessed using a MTT assay based on the modified approach reported by Ferrari and colleagues [35]. Nutlin-3 [19], known as P53/MDM2 interaction inhibitor, were also tested on some cell lines for comparison. The results of the cytotoxicity study are summarized in Table 1.

As shown in Table 1, the most potent compounds from the **4a–x** series exhibited a CC_50_ value in the range of 1.1–12.6 µM against the used cells’ panel. However, no relevant selectivity was observed among the cell types, although compound **4f**, for example, exhibits a cytotoxic effect that exceeds the effect of the nutlinreference sample. Therefore, this compound demonstrated rather overt cytotoxic effect. Seven compounds (**4a–f**, **4u**, **4f**, **6r**, **9r**) were found to be selective on LNCaP cells over PC3 cells. Among them, the upper selectivity towards the remaining cells was observed for compounds **4e, 4f, 4u, 4w**. Compounds **4e, 4f, 4u, 4w** showed the best selectivity index (S value for cell line pairs is defined as ratio of its CC_50_ values) for LNCaP/PC3 cells (>30, 2.8, 15.7, 5.3, respectively). Compounds **4a, 4b, 4d, 4f** showed moderate selectivity and efficiency. The negative response of PC3 cells to the treatment with the compounds having high S values possible may be associated with MDM2/p53 mode of action due to p53 tumor suppressor pathway in this cell type which is, in most cases, disrupted by human papilloma virus (HPV) [36]. The activated p53 induces the transcription of MDM2, which can directly interact with transactivation domain of p53 thereby inhibiting its transcription activity by targeting it for polyubiquitination and further proteasome-mediated degradation [37]. In many cancer cells, including HepG2, Hek and MCF-7, the overexpression of MDM2 gene is actually observed resulting in significant apoptosis attenuation. However, the obtained results do not allow to draw an unambiguous conclusion whether the studied compounds are actually involved in the direct activation of p53.

For instance, compound **4f** inhibited the proliferation of LNCaP and PC3 cells with CC_50_ values of 4.5 ± 0.32 µM and 12.6 ± 2.3 µM, respectively, in contrast to its close structural analogue **4h** with no activity at all (the difference is in *p*-position of the phenyl ring: compound **4f** contains methoxy substituent while compound **4h** ethoxy group). It can be primarily attributed to steric clashes; however, this hypothesis is under debate because of compounds **4i** and **4j** with methyl group in *p*-position weakly inhibited cell growth across the panel as compared to compound **4f**. This may be partly explained by an additional hydrogen bond that can be provided by OMe group. To further elucidate the dominant mode of action, we used HCT116^+/+^ and HCT116^−/−^ cells by analogy with the paper published by Shangary and colleagues [38]. This isogenic cell line is commonly applied to investigate the p53/MDM2-dependent mode of action. Compounds, **4p**, **4u** and **4w** showed a significant selectivity against HCT116^+/+^ over HCT116^−/−^, with absolute S values which are >2.9, >1.8 and >4.8, respectively. For the control sample, etoposide (a topoisomerase poison [39]), the selectivity index was 1.97, therefore compounds with S > 2 should be rather classified as having poor selectivity. Under the same conditions, nutlin-3, the knownp53/MDM2-interaction inhibitor [19] was found to be also active and selective against HCT116^+/+^ cells over HCT116^−/−^ cells showing CC_50_ values of 3.3 ± 0.13 µM and 35.12 ± 2.65 µM, respectively, and S = 10.6. Summarizing, based on the assay performed, only three thiohydantoine derivatives, **4p**, **4u** and **4w**, can be reasonably regarded as the most promising candidates for further evaluation and optimization. 

Interestingly, although compound **4r** was absolutely inactive against LNCaP and PC3 cells, both their analogues, as **6r** (selenohydantoin derivative) and as **9r** (hydantoin derivative) showed good activity vs. LNCaP line over PC3 cells. 

#### 2.2.2. Molecular Docking Study

**Figure 3:**
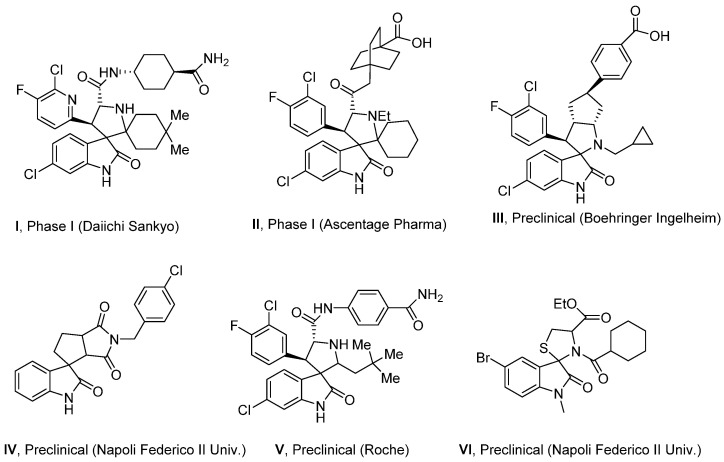
Examples of small-molecule MDM2 inhibitors with spiro-oxindole fragments in the molecules [40,41,42,43,44].

To further investigate the possibility of the obtained spiro derivatives to inhibit the interaction of P53 and MDM2 proteins, we studied them using the molecular docking. Initially, we have collected a database of some analogues of the compounds **4** scaffold (**I**–**VI**, Figure 3) [3,4,14,40,41] and speculated that small-molecule MDM2 inhibitors are the most similar in structure to our series. All compounds selected for comparison contained an oxindole fragment spiro-conjugated with the pyrrolidine ring. For instance, compound **II** has shown an IC_50_ value of 22 µM (mitogenesis inhibition, dye assay, WST-8) against PC3 human prostate adenocarcinoma cells (p53-null) [41], while under the same conditions towards LNCaP cells (androgen-dependent), it has demonstrated IC_50_ = 18 ± 13 nM [41].

Cytotoxicity of the compound **II** has been evaluated also against HCT116^+/+^ and HCT116^−/−^ human colon carcinoma cells [41]. It has shown 80-fold selectivity towards p53-positive cells with IC_50_ values of 0.1 µM (vs. HCT116^+/+^) and 8 µM (vs. HCT116^−/−^), respectively. Compound **IV** demonstrated a CC_50_ value of 0.44 µM against HEK293 cells (24 h cytotoxicity, MTT assay) [14]. Compound **V** was reported to inhibit the growth and progression of HCT116 cells with an IC_50_ value of 90 nM (MTT assay) [4]. Molecule **VI** was evaluated against MCF7 cells (hormone-dependent) and PC3 cell line [42]. As a result, it showed high cytotoxicity and provided IC_50_ values of 40 nM and 0.41 µM, respectively. These reference activities are comparable with that observed for the most active compounds disclosed in the current work. The protein–protein interaction between MDM2 and p53 is observed via the first ∼120 *N*-terminal amino acid (AA) residues of MDM2 and the first 30 *N*-terminal AAs of p53 [43]. Twenty years ago, the first high-resolution co-crystal structure of MDM2 with a p53 peptide (residues 15–29, PDB code 1YCR) was reported by Kussie and co-workers [44]. Since, more than 50 crystallographic complexes have been published for a variety of small-molecule MDM2/p53 inhibitors belonging to different classes, including spiro-oxindoles.

The analysis of MDM2/p53 binding interface revealed that MDM2-bound p53 peptide adopts an α-helical conformation and interacts with MDM2 primarily through the hydrophobic triad of Phe19, Trp23 and Leu26. These “*trident*” of i, i + 4, i + 7 binds tightly into a medium-sized pocket in the structure of MDM2. This compact and well-defined binding site has been used to design many small-molecule high-affinity MDM2 inhibitors which effectively block the MDM2/p53 interaction thereby blocking tumor growth and progression. To elucidate the possible binding affinity of the evaluated compounds toward MDM2, a static 3D molecular docking study was performed in ICM-Pro software [45] based on several available X-ray data, including 4JVR, 4MDQ, 4JWR as well as 5C5A, 5HMH and 4ZYF. The binding site for 3D-molecular docking study was constructed following the standard procedure of binding site preparation in ICM-Pro with default settings. The procedure included the following steps: converting PDB-file to ICM-Pro object, optimizing hydrogens, excluding water molecules, moving template ligand out from pocket, and constructing receptor maps also with default settings in ICM-Pro. 

The binding site for 3D-molecular docking study was constructed following the standard procedure of binding site preparation in ICM-Pro with default settings. The procedure included the following steps: converting PDB-file to ICM-Pro object, optimizing hydrogens, excluding water molecules, moving template ligand out from pocket, and constructing receptor maps also with default settings in ICM-Pro. The binding site was then compared with the binding mode revealed recently for recombinant p53 binding domain (residues 17–125) [21].

**Figure 4:**
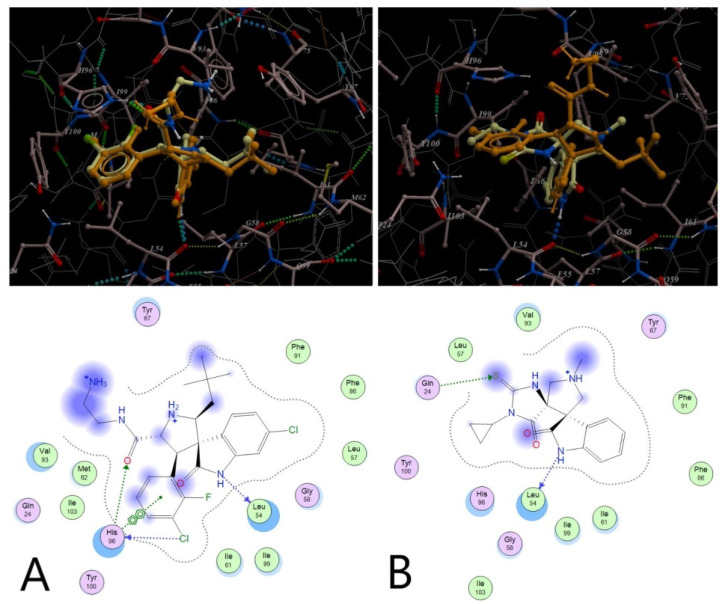
The results of moleculardocking study: (**A**) MDM2 inhibitor **V** (see Figure 3) bound in the target pocket (4JVR)—RSA data (orange), the predicted active conformation (yellow, E^b^ = −88 kcal/mol), RMSD = 0.23; (**B**) compound **4u** (orange, E^b^ = −53 kcal/mol) and the reference compound **V** (yellow) docked into the same binding site (the best conformations are shown).

The validation of the constructed docking model was performed using the reference compounds as they were found in PDB-files. For the 4JVR-based model particularly, we have provided the results of molecular docking study as compared to original X-ray data (Figure 4A, compound V docked into the binding site in the same conformation with RMSD = 0.23). The reference compounds were then docked into the constructed model starting from 2D or 3D structures with or without stereo assignment. The obtained results (Figure 4A) were well correlated with the published RSA data. The most promising dispiro compound **4u**, described in this work, was then docked into the static pocket using an extensive range of key force-fields, particularly describing hydrophobic interactions. As shown in Figure 4B, the selected molecule has a very similar to the reference compound binding mode. However, the predicted active conformation is distinct from that published for other MDM2 inhibitors. Thus, methyl group of compound **4u** is located in a deep cavity by analogy to most of the reported 5-halogen substituted oxindoles.

Thus, although the mode of binding for the compound **4u** areambiguous, its scaffold has the 3D-pharmacophore elements critical for binding to the pre-defined MDM2 pocket as compared to the reported MDM2/p53 inhibitors, including other spiro-oxindoles.

#### 2.2.3. P53 Activation 

In an effort to further elucidate the underlying mechanism of action of the compounds **4–6** we have performed cell-based assay with the p53 reporter construction [46] particularly sensitive to MDM2 inhibitors. In general, the obtained results demonstrated that the activation of p53 was observed upon the treatment with high concentrations of all synthesized compounds (>100 μM). This concentration is close to highly cytotoxic range (only 7–10% of cells stayed alive). In the same concentrations, 2.1-fold p53 activation was observed for compound **9r**; under the same conditions, nutlin-3a showed from 3.6- and up to 5.1-fold increase in the p53 activation [47]. The effect of compounds **4** was slightly higher than the threshold value and could presumably be attributed to p53 activation primarily due to cell death and not vice versa.

Thus, although nutlin, chosen as a reference molecule, is comparable to the compound **4f** in terms of cytotoxicity, unlike nutlin, the MDM2 protein is apparently not the main target of the compounds described in this work

## 3. Materials and Methods

### 3.1. General Information

All common reagents were purchased from commercial suppliers and used as received. The melting points are uncorrected.^1^H-NMR spectra were recorded on Bruker Avance 400 and Agilent MR-400 spectrometers at 400 MHz in CDCl_3_ or DMSO-d_6_. Chemical shifts were measured relative to solvent residual signals and referenced in part per million to TMS. Chemical shifts are reported in parts per million relative to TMS. High resolution mass spectra (HRMS) were recorded on an OrbitrapElite (Thermo Scientific) mass spectrometer with electrospray ionization (ESI) and orbital trap. To inject solutions with a concentration of 0.1 to 9 mg/mL (in 1% formic acid in acetonitrile), direct injection into the ion source using a syringe pump (5 mL/min) was used. The spray voltage was ±3.5 kV, the temperature of the capillary was 275 °C.

Compound **2a** was synthesized as described in [48,49].

X-ray study was performed on diffractometer Bruker APEX DUO (MoKα-radiation, graphite monochromator, φ-scan). The X-ray structure was solved by direct methods and refined using full-matrix anisotropic approximation for F^2^_hkl_. The location of the hydrogen atoms was predicted geometrically, their positions were well-adjusted using the “rider” model Uiso(H) = 1.5Ueq(C) formethyl groups and 1.2 Ueq(X) for the remaining H-atoms. All calculations were performed in SHELX software version 2015 [50] and OLEX-2 [51].

CCDC 2120852 contains the supplementary crystallographic data for this paper. These data can be obtained free of charge via www.ccdc.cam.ac.uk/data_request/cif (Embargoed Date 8 September 2022), or by emailing data_request@ccdc.cam.ac.uk, or by contacting The Cambridge Crystallographic Data Centre, 12 Union Road, Cambridge CB2 1EZ, UK; fax: +44 1223 336033.

### 3.2. Synthesis

#### 3.2.1. General Procedure for the Synthesis of Thioureas (**1**) and Thiohydantoins (**2**)

Amine (1 equiv) was added to a solution of ethyl isothiocyanatoacetate (1 equiv) in ether. The resulting mixture was stirred for 1 hour at room temperature. After the reaction was completed (TLC control), the solvent was evaporated in vacuo and the formed precipitate was filtered off, washed with cold diethyl ether and dried in air.

**Ethyl 2-(3-benzylthioureido)acetate (1a)**

From 0.54 g (5.0 mmol) of benzylamine and 0.73 g (5.0 mmol) of ethyl isothiocyanatoacetate, compound **1a** (1.0 g, 83%) was obtained as a white solid.^1^H-NMR (400 MHz, CDCl_3_) δ: 8.25 (bs, 1H, NH), 7.40 (d, *J* = 8.7 Hz, 2H), 7.26 (d, *J* = 8.7 Hz, 2H), 6.70 (bs, 1H, NH), 4.42 (s, 2H), 4.22 (q, *J* = 9.2 Hz, 2H), 1.29 (t, *J* = 9.2 Hz, 3H). HRMS (ESI+) *m/z* calcd. for (C_12_H_16_N_2_O_2_S, M + H): 253.1005, found: (M + H): 253.1015.

**Ethyl 2-(3-allylthioureido)acetate (1b)**

From 0.29 g (5.0 mmol) of allylamine and 0.73 g (5.0 mmol) of ethyl isothiocyanatoacetate, compound **1b** (0.93 g, 98%) was obtained as a yellow oil. ^1^H-NMR (400 MHz, CDCl_3_) δ: 6.82 (bs, 1H, NH), 6.74 (s, 1H, NH), 5.85 (m, 1H), 5.28 (d, *J* = 17.1 Hz, 1H), 5.20 (d, *J* = 10.2 Hz, 1H), 4.38 (d, *J* = 4.9 Hz, 1H), 4.21 (q, *J* = 7.2 Hz, 2H), 4.05 (s, 2H), 1.28 (t, *J* = 7.2 Hz, 3H). HRMS (ESI+) *m/z* calcd. for (C_8_H_15_N_2_O_2_S, M + H): 203.0849, found: (M + H): 203.0857.

**Ethyl 2-(3-(4-methoxyphenyl)thioureido)acetate (1c)**

From 0.62 g (5.0 mmol) of 4-methoxyaniline and 0.73 g (5.0 mmol) of ethyl isothiocyanatoacetate, compound **1c** (0.77 g, 61%) was obtained as a light yellow solid. M.p. 94–96 °C. ^1^H-NMR (400 MHz, CDCl_3_) δ: 8.12 (bs, 1H, NH), 7.23 (d, *J* = 8.1 Hz, 2H), 7.16 (d, *J* = 8.3 Hz, 2H), 6.60 (bs, 1H, NH), 4.41 (s, 2H), 4.20 (q, *J* = 7.2 Hz, 2H), 2.36 (s, 3H), 1.27 (t, *J* = 7.2 Hz, 3H). HRMS (ESI+) *m/z* calcd. for (C_12_H_16_N_2_O_3_S, M + H): 269.0954, found: (M + H): 269.0958.

**Ethyl 2-(3-(4-ethoxyphenyl)thioureido)acetate (1d)**

From 0.69 g (5.0 mmol) of 4-ethoxyaniline and 0.73 g (5.0 mmol) of ethyl isothiocyanatoacetate, compound **1d** (1.30 g, 97%) was obtained as a purple solid. M.p. 124–126 °C. ^1^H-NMR (400 MHz, CDCl_3_) δ: 7.90 (bs, 1H, NH), 7.19 (d, *J* = 9.1 Hz, 2H), 6.93 (d, *J* = 8.9 Hz, 2H), 6.44 (bs, 1H, NH), 4.41 (s, 2H), 4.20 (q, *J* = 7.2 Hz, 2H), 4.04 (q, *J* = 7.0 Hz, 2H), 1.42 (t, *J* = 7.0 Hz, 3H), 1.27 (t, *J* = 7.2 Hz, 3H). HRMS (ESI+) *m/z* calcd. for (C_13_H_18_N_2_O_3_S, M + H): 283.1111, found: (M + H): 283.1106.

**Ethyl 2-(3-(p-tolyl)thioureido)acetate (1e)**

From 0.54 g (5.0 mmol) of 4-methylaniline and 0.73 g (5.0 mmol) of ethyl isothiocyanatoacetate, compound **1e** (0.89 g, 75%) was obtained as a plum solid. M.p. 132–134 °C. ^1^H-NMR (400 MHz, CDCl_3_) δ: 7.91 (bs, 1H, NH), 7.21 (d, *J* = 8.9 Hz, 2H), 6.95 (d, *J* = 8.9 Hz, 2H), 6.45 (bs, 1H, NH), 4.41 (s, 2H), 4.20 (q, *J* = 7.2 Hz, 2H), 3.82 (s, 3H), 1.28 (t, *J* = 7.2 Hz, 3H).

**Ethyl 2-(3-(4-chlorophenyl)thioureido)acetate (1f)**

From 0.64 g (5.0 mmol) of 4-chloroaniline and 0.73 g (5.0 mmol) of ethyl isothiocyanatoacetate, compound **1f** (1.16 g, 90%) was obtained as a white solid. M.p. 154–156 °C. ^1^H-NMR (400 MHz, CDCl_3_) δ: 8.25 (bs, 1H, NH), 7.40 (d, *J* = 8.7 Hz, 2H), 7.25 (d, *J* = 8.7 Hz, 2H), 6.70 (bs, 1H, NH), 4.42 (s, 2H), 4.22 (q, *J* = 7.2 Hz, 2H), 1.29 (t, *J* = 7.2 Hz, 3H). HRMS (ESI+) *m/z* calcd. for (C_11_H_13_ClN_2_O_2_S, M + H): 273.0459, found: (M + H): 273.0468.

**Ethyl 2-(3-(4-fluorophenyl)thioureido)acetate (1g)**

From 0.56 g (5.0 mmol) of 4-fluoroaniline and 0.73 g (5.0 mmol) of ethyl isothiocyanatoacetate, compound **1g** (0.97 g, 76%) was obtained as a white solid. M.p. 119–120 °C. ^1^H-NMR (400 MHz, CDCl_3_) δ: 8.15 (bs, 1H, NH), 7.33–7.25 (m, 2H), 7.14 (t, *J* = 8.5 Hz, 2H), 6.56 (bs, 1H, NH), 4.42 (s, 2H), 4.21 (q, *J* = 7.1 Hz, 2H), 1.28 (t, *J* = 7.1 Hz, 3H). HRMS (ESI+) *m/z* calcd. for (C_11_H_13_FN_2_O_2_S, M + H): 257.0755, found: (M + H): 257.0766.

**Ethyl 2-(3-(3-chlorobenzyl)thioureido)acetate (1h)**

From 0.74 g (5.0 mmol) of e-chlorobenzylamine and 0.73 g (5.0 mmol) of ethyl isothiocyanatoacetate. compound **1h** (1.12 g, 84%) was obtained as a white solid. M.p. 122–123 °C. 1H-NMR(400 MHz, CDCl_3_) δ: 7.39–7.19 (m, 5H), 4.67 (bs, 2H), 4.38 (s, 2H), 4.18 (q, *J* = 7.1 Hz, 2H), 1.27 (t, *J* = 7.1 Hz, 3H). HRMS (ESI+) *m/z* calcd. for (C_12_H_15_ClN_2_O_2_S, M + H): 287.0616, found: (M + H): 287.0628.

**Ethyl 2-(3-(3-chloro-4-fluorophenyl)thioureido)acetate (1i)**

From 0.73 g (5.0 mmol) of 4-fluoro,3-chloroaniline and 0.73 g (5.0 mmol) of ethyl isothiocyanatoacetate, compound **1i** (1.08 g, 74%) was obtained as a white solid. M.p. 111–112 °C. ^1^H-NMR (400 MHz, CDCl_3_) δ: 8.26 (bs, 1H, NH), 7.42 (dd, *J*_1_ = 2.4 Hz, *J*_2_ = 6.4 Hz, 1H), 7.26–7.17 (m, 2H), 6.71 (bs, 1H, NH), 4.42 (s, 2H), 4.22 (q, *J* = 7.1 Hz, 2H), 1.30 (t, *J* = 7.2 Hz, 3H). HRMS (ESI+) *m/z* calcd. for (C_11_H_12_ClFN_2_O_2_S, M + H): 291.0365, found: (M + H): 291.0377.

**Ethyl 2-(3-cyclopropylthioureido)acetate (1j)**

From 0.29 g (5.0 mmol) of cyclopropylamine and 0.73 g (5.0 mmol) of ethyl isothiocyanatoacetate, compound **1j** (0.8 g, 87%) was obtained as a white solid. M.p. 129–130 °C. ^1^H-NMR (400 MHz, CDCl_3_) δ: 6.86 (bs, 1H, NH), 6.56 (bs, 1H, NH), 4.45 (d, *J* = 4.5 Hz, 2H), 4.27 (q, *J* = 7.0 Hz, 2H), 2.54 (bs, 1H), 1.32 (t, *J* = 7.2 Hz, 1H), 0.92–0.85 (m, 2H), 0.76–0.69 (m, 2H).

**3-(3-Morpholinopropyl)-2-thioxoimidazolidin-4-one (2b)**

From 0.72 g (5.0 mmol) of 3-(N-morpholino)propylamine and 0.73 g (5.0 mmol) of ethyl isothiocyanatoacetate, compound **1k** (0.82 g, 68%) was obtained as a pink solid. M.p. 149–151 °C. ^1^H-NMR (400 MHz, CDCl_3_) δ: 8.33 (bs, 1H, NH), 4.08 (s, 2H), 3.89 (t, *J* = 6.9 Hz, 2H), 3.82–3.74 (m, 4H), 2.72–2.55 (m, 6H), 2.04–1.93 (m, 2H).

#### 3.2.2. General Procedure for the Synthesis of 5-Substituted-2-thiohydantoins **3a–x**

Thioureidoacetate **1** or 2-thioxoimidazolidine **2** (1 equiv) was dissolved in 2% KOH/EtOH; then isatin or 5-chloroisatin (1 equiv) was added. The resulting mixture was stirred for 30 min. After the reaction was completed (TLC control), the mixture was poured into water and neutralized with HCl. The formed precipitate was filtered off, washed with cold water, then washed with cold diethyl ether and dried in air.

**(Z)-3-(1-Benzyl-5-oxo-2-thioxoimidazolidin-4-ylidene)indolin-2-one (3a)**

From **1a** (0.36 g, 1.5 mmol) and isatin (0.22 g, 1.5 mmol), compound 3a (0.48 g, 94%) was obtained as a red solid. M.p. > 300 °C. ^1^H-NMR (400 MHz, DMSO-d_6_) δ: 11.56 (s, 1H, NH), 11.10 (s, 1H, NH)8.53 (d, *J* = 7.8 Hz, 1H), 7.43–7.26 (m, 6H), 7.03 (td, *J*_1_ = 1.0 Hz, *J*_2_ = 7.7 Hz, 1H), 6.93 (d, *J* = 7.8 Hz, 1H), 5.06 (s, 2H). HRMS (ESI+) *m/z* calcd. for (C_18_H_13_N_3_O_2_S, M + H): 336.0801, found: (M + H): 336.0797.

**(Z)-3-(1-Benzyl-5-oxo-2-thioxoimidazolidin-4-ylidene)-5-chloroindolin-2-one (3b)**

From **1a** (0.36 g, 1.5 mmol) and 5-chloroisatin (0.27 g, 1.5 mmol), compound **3b** (0.51 g, 92%) was obtained as a dark red solid. M.p. > 300 °C. ^1^H-NMR (400 MHz, DMSO-d_6_) δ: 11.63 (s, 1H, NH), 11.18 (s, 1H, NH),8.55 (m, 1H), 7.43–7.25 (m, 6H), 6.92 (d, *J* = 8.3 Hz, 1H), 5.05 (s, 2H). HRMS (ESI+) *m/z* calcd. for (C_18_H_12_ClN_3_O_2_S, M + H): 370.0411, found: (M + H): 370.0411.

**(Z)-3-(1-Allyl-5-oxo-2-thioxoimidazolidin-4-ylidene)indolin-2-one (3c)**

From **1b** (0.28 g, 1.5 mmol) and isatin (0.22 g, 1.5 mmol,) compound **3c** (0.36 g, 83%) was obtained as a red solid. M.p. 257–259 °C. ^1^H-NMR (400 MHz, DMSO-d_6_) δ: 11.50 (bs, 1H, NH), 11.07 (s, 1H, NH),8.52 (d, *J* = 7.8 Hz, 1H), 7.32 (td, *J*_1_ = 1.0 Hz, *J*_2_ = 7.7 Hz, 1H), 7.04 (td, *J*_1_ = 0.7 Hz, *J*_2_ = 7.7 Hz, 1H), 6.92 (d, *J* = 7.8 Hz, 1H), 5.86 (m, 1H), 5.20 (dd, *J*_1_ = 1.0 Hz, *J*_2_ = 9.7 Hz, 1H), 5.17 (m, 1H), 4.45 (d, *J* = 5.0 Hz, 2H). HRMS (ESI+) *m/z* calcd. for (C_14_H_11_N_3_O_2_S, M + H): 286.0644, found: (M + H): 286.0643.

**(Z)-3-(1-Allyl-5-oxo-2-thioxoimidazolidin-4-ylidene)-5-chloroindolin-2-one (3d)**

From **1b** (0.28 g, 1.5 mmol) and 5-chloroisatin (0.27 g, 1.5 mmol), compound **3d** (0.40 g, 83%) was obtained as a dark red solid. M.p. 258–260 °C. ^1^H-NMR (400 MHz, DMSO-d_6_) δ: 11.56 (bs, 1H, NH), 11.19 (s, 1H, NH),8.55 (d, *J* = 2.0 Hz, 1H), 7.36 (dd, *J*_1_ = 2.2 Hz, *J*_2_ = 8.3 Hz, 1H), 6.93 (d, *J* = 8.31 Hz, 1H), 5.86 (m, 1H), 5.25–5.16 (m, 2H), 4.45 (d, *J* = 5.1 Hz, 2H). HRMS (ESI+) *m/z* calcd. for (C_14_H_10_ClN_3_O_2_S, M + H): 320.0255, found: (M + H): 320.0252.

**(Z)-3-(1-(4-Methoxyphenyl)-5-oxo-2-thioxoimidazolidin-4-ylidene)indolin-2-one (3e)**

From **1c** (0.38 g, 1.5 mmol) and isatin (0.22 g, 1.5 mmol), compound **3e** (0.49 g, 93%) was obtained as a red solid. M.p. > 300 °C. ^1^H-NMR (400 MHz, DMSO-d_6_) δ: 11.60 (bs, 1H, NH), 10.95 (bs, 1H, NH), 8.56 (d, *J* = 7.70 Hz, 1H), 7.32 (d, *J* = 8.1 Hz, 2H), 7.29–7.22 (m, 3H), 6.97 (t, *J* = 7.6 Hz, 1H),6.90 (d, *J* = 7.8 Hz, 1H), 2.38 (s, 3H). HRMS (ESI+) *m/z* calcd. for (C_18_H_13_N_3_O_3_S, M + H): 352.0750, found: (M + H): 352.0771.

**(Z)-5-Chloro-3-(1-(4-methoxyphenyl)-5-oxo-2-thioxoimidazolidin-4-ylidene)indolin-2-one (3f)**

From **1c** (0.38 g, 1.5 mmol) and 5-chloroisatin (0.27 g, 1.5 mmol), compound **3f** (0.54 g, 94%) was obtained as a dark red solid. M.p. > 300 °C. ^1^H-NMR (400 MHz, DMSO-d_6_) δ: 11.69 (bs, 1H, NH), 11.24 (bs, 1H, NH), 8.54 (d, *J* = 2.2 Hz, 1H), 7.36–7.29 (m, 5H), 6.96 (dd, *J*_1_ = 2.2 Hz, *J*_2_ = 8.3 Hz, 1H), 2.39 (s, 3H). HRMS (ESI+) *m/z* calcd. for (C_18_H_12_ClN_3_O_3_S, M + H): 386.0360, found: (M + H): 386.0387.

**(Z)-3-(1-(4-Ethoxyphenyl)-5-oxo-2-thioxoimidazolidin-4-ylidene)indolin-2-one (3g)**

From **1d** (0.40 g, 1.5 mmol) and isatin (0.22 g, 1.5 mmol), compound **3g** (0.49 g, 89%) was obtained as a red solid. M.p. > 300 °C. ^1^H-NMR (400 MHz, DMSO-d_6_) δ: 11.62 (bs, 1H, NH), 11.14 (s, 1H, NH), 8.50 (d, *J* = 7.6 Hz, 1H), 7.37–7.29 (m, 3H), 7.06 (d, *J* = 8.9 Hz, 2H), 7.02 (t, *J* = 7.7 Hz, 1H), 6.95 (d, *J* = 8.0 Hz, 1H), 4.09 (q, *J* = 6.9 Hz, 2H), 1.36 (t, *J* = 6.9 Hz, 3H). HRMS (ESI+) *m/z* calcd. for (C_19_H_15_N_3_O_3_S, M + H): 366.0906, found: (M + H): 366.0895.

**(Z)-5-Chloro-3-(1-(4-ethoxyphenyl)-5-oxo-2-thioxoimidazolidin-4-ylidene)indolin-2-one (3h)**

From **1d** (0.40 g, 1.5 mmol) and 5-chloroisatin (0.27 g, 1.5 mmol), compound **3h** (0.52 g, 88%) was obtained as a dark red solid. M.p. > 300 °C. ^1^H-NMR (400 MHz, DMSO-d_6_) δ: 11.66 (bs, 1H, NH), 11.23 (s, 1H, NH),8.55 (s, 1H), 7.37 (dd, *J*_1_ = 2.2 Hz, *J*_2_ = 8.4 Hz, 1H), 7.33 (d, *J* = 8.7 Hz, 2H), 7.07 (d, *J* = 8.6 Hz, 2H), 6.96 (d, *J* = 8.3 Hz, 1H), 4.09 (q, *J* = 6.8 Hz, 2H), 1.36 (t, *J* = 6.8 Hz, 3H). HRMS (ESI+) *m/z* calcd. for (C_19_H_15_ClN_3_O_3_S, M + H): 400.0517, found: (M + H): 400.0496.

**(Z)-3-(5-oxo-2-thioxo-1-(p-tolyl)imidazolidin-4-ylidene)indolin-2-one (3i)**

From **1e** (0.36 g, 1.5 mmol) and isatin (0.22 g, 1.5 mmol), compound **3i** (0.48 g, 95%) was obtained as a red solid. M.p. > 300 °C. ^1^H-NMR (400 MHz, DMSO-d_6_) δ: 11.62 (s, 1H, NH), 11.14 (s, 1H, NH), 8.50 (d, *J* = 7.8 Hz, 1H), 7.38–7.30 (m, 3H), 7.09 (d, *J* = 8.8 Hz, 2H), 7.03 (t, *J* = 7.6 Hz, 1H), 6.95 (d, *J* = 7.7 Hz, 1H), 3.83 (s, 3H). HRMS (ESI+) *m/z* calcd. for (C_18_H_13_N_3_O_2_S, M + H): 336.0801, found: (M + H): 336.0796.

**(Z)-5-Chloro-3-(5-oxo-2-thioxo-1-(p-tolyl)imidazolidin-4-ylidene)indolin-2-one (3j)**

From **1e** (0.36 g, 1.5 mmol) and 5-chloroisatin (0.27 g, 1.5 mmol), compound **3j** (0.51 g, 92%) was obtained as a dark red solid. M.p. > 300 °C. ^1^H-NMR (400 MHz, DMSO-d_6_) δ: 11.67 (bs, 1H, NH), 11.25 (s, 1H, NH),8.55 (d, *J* = 1.4 Hz, 1H), 7.40–7.32 (m, 3H), 7.09 (d, *J* = 8.8 Hz, 2H), 6.96 (d, *J* = 8.3 Hz, 1H), 3.83 (s, 3H). HRMS (ESI+) *m/z* calcd. for (C_18_H_12_ClN_3_O_2_S, M + H): 370.0411, found: (M + H): 370.0414.

**(Z)-3-(1-(4-Chlorophenyl)-5-oxo-2-thioxoimidazolidin-4-ylidene)indolin-2-one (3k)**

From **1f** (0.39 g, 1.5 mmol) and isatin (0.22 g, 1.5 mmol), compound **3k** (0.45 g, 84%) was obtained as a red solid. M.p. > 300 °C. ^1^H-NMR (400 MHz, DMSO-d_6_) δ: 11.69 (bs, 1H, NH), 11.14 (bs, 1H, NH),8.48 (d, *J* = 7.7 Hz, 1H), 7.63 (d, *J* = 8.4 Hz, 2H), 7.48 (d, *J* = 8.4 Hz, 2H), 7.32 (t, *J* = 7.7 Hz, 1H), 7.02 (t, *J* = 7.7 Hz, 1H), 6.94 (d, *J* = 7.7 Hz, 1H). HRMS (ESI+) *m/z* calcd. for (C_17_H_10_ClN_3_O_2_S, M + H): 356.0255, found: (M + H): 356.0257.

**(Z)-5-Chloro-3-(1-(4-chlorophenyl)-5-oxo-2-thioxoimidazolidin-4-ylidene)indolin-2-one (3l)**

From **1f** (0.39 g, 1.5 mmol) and 5-chloroisatin (0.27 g, 1.5 mmol), compound **3l** (0.51 g, 86%) was obtained as a dark red solid. M.p. > 300 °C. ^1^H-NMR (400 MHz, DMSO-d_6_) δ: 11.76 (bs, 1H, NH), 11.24 (s, 1H, NH), 8.53 (d, *J* = 2.0 Hz, 1H), 7.65 (d, *J* = 8.6 Hz, 2H), 7.49 (d, *J* = 8.6 Hz, 2H), 7.38 (dd, *J*_1_ = 2.1 Hz, *J*_2_ = 8.3 Hz, 1H), 6.96 (d, *J* = 8.3 Hz, 1H). HRMS (ESI+) *m/z* calcd. for (C_17_H_9_Cl_2_N_3_O_2_S, M + H): 389.9865, found: (M + H): 389.9846.

**(Z)-3-(1-(4-Fluorophenyl)-5-oxo-2-thioxoimidazolidin-4-ylidene)indolin-2-one (3m)**

From **1g** (0.38 g, 1.5 mmol) and isatin (0.22 g, 1.5 mmol), compound **3m** (0.45 g, 89%) was obtained as a red solid. M.p. > 300 °C. ^1^H-NMR (400 MHz, DMSO-d_6_) δ: 11.68 (s, 1H, NH), 11.13 (s, 1H, NH), 8.50 (d, *J* = 7.0 Hz, 1H), 7.58–7.46 (m, 2H), 7.40 (t, *J* = 8.0 Hz, 2H), 7.33 (t, 7.5 Hz, 1H), 7.03 (t, *J* = 7.0 Hz, 1H), 6.95 (d, *J* = 7.0 Hz, 1H). HRMS (ESI-) *m/z* calcd. for (C_17_H_10_FN_3_O_2_S, M-H): 338.0394, found: (M-H): 338.0405.

**(Z)-5-Chloro-3-(1-(4-fluorophenyl)-5-oxo-2-thioxoimidazolidin-4-ylidene)indolin-2-one (3n)**

From **1g** (0.38 g, 1.5 mmol) and 5-chloroisatin (0.27 g, 1.5 mmol), compound **3n** (0.51 g, 91%) was obtained as a red solid. M.p. > 300 °C. ^1^H-NMR (400 MHz, DMSO-d_6_) δ: 11.75 (s, 1H, NH), 11.24 (s, 1H, NH), 8.56–8.52 (m, 1H), 7.55–7.48 (m, 2H), 7.46–7.35 (m, 3H), 6.96 (dd, *J*_1_ = 3.6 Hz, *J*_2_ = 8.3 Hz, 1H). HRMS (ESI-) *m/z* calcd. for (C_17_H_9_ClFN_3_O_2_S, M-H): 372.0004, found: (M-H): 372.0017.

**(Z)-3-(1-(3-Chlorobenzyl)-5-oxo-2-thioxoimidazolidin-4-ylidene)indolin-2-one (3o)**

From **1h** (0.43 g, 1.5 mmol) and isatin (0.22 g, 1.5 mmol), compound **3o** (0.48 g, 87%) was obtained as a red solid. M.p. 296–298 °C. ^1^H-NMR (400 MHz, DMSO-d_6_) δ: 11.59 (bs, 1H, NH), 11.09 (s, 1H, NH), 8.52 (d, *J* = 7.7 Hz, 1H), 7.46 (s, 1H), 7.40–7.29 (m, 4H), 7.03 (t, *J* = 7.6 Hz, 1H), 6.93 (d, *J* = 7.8 Hz, 1H), 5.05 (s, 2H). HRMS (ESI-) *m/z* calcd. for (C_18_H_12_ClN_3_O_2_S, M-H): 368.0266, found: (M-H): 368.0255.

**(Z)-5-Chloro-3-(1-(3-chlorobenzyl)-5-oxo-2-thioxoimidazolidin-4-ylidene)indolin-2-one (3p)**

From **1h** (0.43 g, 1.5 mmol) and 5-chloroisatin (0.27 g, 1.5 mmol), compound **3p** (0.53 g, 87%) was obtained as a red solid. M.p. > 300 °C. ^1^H-NMR (400 MHz, DMSO-d_6_) δ: 11.66 (bs, 1H, NH), 11.18 (s, 1H, NH), 8.56 (d, *J* = 1.7 Hz, 1H), 7.46 (s, 1H), 7.41–7.32 (m, 4H), 6.94 (d, *J* = 8.3 Hz, 1H), 5.05 (s, 2H). HRMS (ESI-) *m/z* calcd. for (C_18_H_11_Cl_2_N_3_O_2_S, M-H): 401.9865, found: (M-H): 401.9879.

**(Z)-3-(1-(3-Chloro-4-fluorophenyl)-5-oxo-2-thioxoimidazolidin-4-ylidene)indolin-2-one (3q)**

From **1i** (0.44 g, 1.5 mmol) and isatin (0.22 g, 1.5 mmol), compound **3q** (0.47 g, 84%) was obtained as a red solid. M.p. > 300 °C. ^1^H-NMR (400 MHz, DMSO-d_6_) δ: 11.75 (bs, 1H, NH), 10.92 (s, 1H, NH), 8.56 (d, *J* = 7.8 Hz, 1H), 7.73 (dd, *J*_1_ = 1.88 Hz, *J* = 6.5 Hz, 1H), 7.61 (t, *J* = 9.0 Hz, 1H), 7.48 (m, 1H), 7.27 (t, *J* = 7.3 Hz, 1H), 6.98 (t, *J* = 7.6 Hz, 1H), 6.90 (d, *J* = 7.6 Hz, 1H). HRMS (ESI-) *m/z* calcd. for (C_17_H_9_ClFN_3_O_2_S, M-H): 372.0004, found: (M-H): 372.0017.

**(Z)-5-Chloro-3-(1-(3-chloro-4-fluorophenyl)-5-oxo-2-thioxoimidazolidin-4-ylidene)indolin-2-one (3r)**

From **1i** (0.44 g, 1.5 mmol) and 5-chloroisatin (0.27 g, 1.5 mmol), compound **3r** (0.53 g, 86%) was obtained as a red solid. M.p. > 300 °C. ^1^H-NMR (400 MHz, DMSO-d_6_) δ: 11.81 (bs, 1H, NH), 11.24 (s, 1H, NH), 8.51 (d, *J* = 1.8 Hz, 1H), 7.77 (dd, *J*_1_ = 2.3 Hz, *J*_2_ = 6.7 Hz, 1H), 7.65 (t, *J* = 8.99 Hz, 1H), 7.52 (m, 1H), 7.37 (dd, *J*_1_ = 2.02 Hz, *J*_2_ = 8.38 Hz, 1H), 6.95 (d, *J* = 8.3 Hz, 1H). HRMS (ESI-) *m/z* calcd. for (C_17_H_8_Cl_2_FN_3_O_2_S, M-H): 405.9615, found: (M-H): 405.9628.

**(Z)-3-(1-Cyclopropyl-5-oxo-2-thioxoimidazolidin-4-ylidene)indolin-2-one (3s)**

From **1j** (0.30 g, 1.5 mmol) and isatin (0.22 g, 1.5 mmol), compound **3s** (0.26 g, 92%) was obtained as a red solid. M.p. 277–279 °C (decomp.). ^1^H-NMR (400 MHz, DMSO-d_6_) δ: 11.35 (bs, 1H, NH), 11.08 (s, 1H, NH), 8.52 (d, *J* = 7.8 Hz, 1H), 7.32 (t, *J* = 7.6 Hz, 1H), 7.04 (t, *J* = 7.6 Hz, 1H), 6.92 (d, *J* = 7.8 Hz, 1H), 2.80 (m, 1H), 1.04–0.98 (m, 4H). HRMS (ESI-) *m/z* calcd. for (C_14_H_11_N_3_O_2_S, M-H): 284.0488, found: (M-H): 284.0499.

**(Z)-5-Chloro-3-(1-cyclopropyl-5-oxo-2-thioxoimidazolidin-4-ylidene)indolin-2-one (3t)**

From **1j** (0.30 g, 1.5 mmol) and 5-chloroisatin (0.27 g, 1.5 mmol), compound **3t** (0.29 g, 92%) was obtained as a red solid. M.p. 286–288 °C (decomp.). ^1^H-NMR (400 MHz, DMSO-d_6_) δ: 11.40 (s, 1H, NH), 11.19 (s, 1H, NH), 8.56 (m, 1H), 7.36 (d, *J* = 8.3 Hz, 1H), 6.94 (dd, *J*_1_ = 2.1 Hz, *J* = 8.3 Hz, 1H), 2.80 (m, 1H), 1.04–0.99 (m, 4H). HRMS (ESI-) *m/z* calcd. for (C_14_H_10_ClN_3_O_2_S, M-H): 318.0099, found: (M-H): 318.0112.

**(Z)-3-(1-(3-Morpholinopropyl)-5-oxo-2-thioxoimidazolidin-4-ylidene)indolin-2-one hydrochloride (3u)**

From **2a** (0.37 g, 1.5 mmol) and isatin (0.22 g, 1.5 mmol), compound **3u** (0.57 g, 93%) was obtained as a red solid. M.p. 270–272 °C. ^1^H-NMR (400 MHz, DMSO-d_6_) δ: 11.54 (s, 1H), 11.18 (s, 1H), 11.07 (bs, 1H), 8.54 (d, *J* = 8.0 Hz, 1H), 7.33 (t, *J* = 7.7 Hz, 1H), 7.05 (t, *J* = 7.7 Hz, 1H), 6.95 (d, *J* = 7.7 Hz, 1H), 3.96–3.88 (m, 4H), 3.78 (t, *J* = 11.8 Hz, 2H), 3.36 (d, *J* = 11.8 Hz, 2H), 3.22–3.13 (m, 2H), 3.07–2.95 (m, 2H), 2.19–2.09 (m, 2H). HRMS (ESI+) *m/z* calcd. for (C_18_H_20_N_4_O_3_S, M + H): 373.1328, found: (M + H): 373.1322.

**(Z)-5-Chloro -3-(1-(3-morpholinopropyl)-5-oxo-2-thioxoimidazolidin-4-ylidene)indolin-2-one hydrochloride (3v)**

From **2a** (0.37 g, 1.5 mmol) and 5-chloroisatin (0.27 g, 1.5 mmol), compound **3v** (0.63 g, 95%) was obtained as a dark red solid. M.p. 249–251 °C. ^1^H-NMR (400 MHz, DMSO-d_6_) δ: 11.61 (s, 1H), 11.31 (s, 1H), 11.09 (bs, 1H), 8.58 (d, *J* = 2.0 Hz, 1H), 7.37 (dd, *J*_1_ = 2.2 Hz, *J*_2_ = 8.4 Hz, 1H), 6.97 (d, *J* = 8.3 Hz, 1H), 3.96–3.88 (m, 4H), 3.85–3.75 (m, 4H), 3.35 (d, *J* = 11.6 Hz, 2H), 3.22–3.13 (m, 2H), 3.07–2.95 (m, 2H), 2.20–2.10 (m, 2H). HRMS (ESI+) *m/z* calcd. for (C_18_H_19_Cl_1_N_4_O_3_S, M + H): 407.0939, found: (M + H): 407.0917.

**(Z)-3-(5-Oxo-1-phenyl-2-thioxoimidazolidin-4-ylidene)indolin-2-one (3w)**

From **2b** (0.29 g, 1.5 mmol) and isatin (0.22 g, 1.5 mmol), compound **3w** (0.45 g, 93%) was obtained as a red solid. M.p. > 300 °C. ^1^H-NMR (400 MHz, DMSO-d_6_) δ: 11.57 (s, 1H, NH), 10.87 (s, 1H, NH), 8.46 (d, *J* = 7.8 Hz, 1H), 7.52–7.41 (m, 3H), 7.37–7.33 (m, 2H), 7.21 (td, *J*_1_ = 1.1 Hz, *J*_2_ = 7.7 Hz, 1H), 6.91 (td, *J*_1_ = 1.0 Hz, *J*_2_ = 7.7 Hz, 1H), 6.86 (d, *J* = 7.7 Hz, 1H). HRMS (ESI-) *m/z* calcd. for (C_17_H_11_N_3_O_2_S, M-H): 320.0488, found: (M-H): 320.0506.

**(Z)-5-Chloro-3-(5-oxo-1-phenyl-2-thioxoimidazolidin-4-ylidene)indolin-2-one (3x)**

From **2b** (0.29 g, 1.5 mmol) and 5-chloroisatin (0.27 g, 1.5 mmol), compound three times (0.50 g, 94%) was obtained as a red solid. M.p. > 300 °C. ^1^H-NMR (400 MHz, DMSO-d_6_) δ: 11.74 (bs, 1H, NH), 11.25 (s, 1H, NH), 8.55 (m, 1H), 7.59–7.49 (m, 3H), 7.47–7.42 (m, 2H), 7.37 (m, 1H), 6.96 (d, *J* = 8.4 Hz, 1H). HRMS (ESI-) *m/z* calcd. for (C_17_H_10_ClN_3_O_2_S, M-H): 354.0099, found: (M-H): 354.0113.

#### 3.2.3. General Procedure for the Synthesis of Dispiroindolinones **4a**–**x**

Corresponding 5-indolidene-2-thioxoimidazolidin **3** (1equiv) and sarcosine (4 equiv) were dissolved in toluene and the mixture heated to a boiling point. After that, paraformaldehyde (4 equiv) was added. The resulting mixture was refluxed for 5–8 hours (TLC control). After the reaction was completed, the solvent was evaporated in vacuo. The product was then purified using column chromatography (silica gel 60, 0.04–0.063 mm/230–400 mesh, CHCl_3_:MeOH/50:1) to afford products as a yellow or pink solid. This solid was washed with acetone to yield corresponding dispirooxindole as white crystalline solid.

**1′-Methyl-1-benzyl-2-thioxodispiro[imidazolidine-4,3′-pyrrolidine-4′,3″-indoline]-2″,5-dione (4a)**

From **3a** (0.22 g, 0.65 mmol), sarcosine (0.23 g, 2.6 mmol) and paraformaldehyde (0.08 g, 2.6 mmol), compound **4a** (0.21 g, 82%) was obtained as a white solid. M.p. 189–190 °C. ^1^H-NMR (400 MHz, DMSO-d_6_) δ: 10.42 (bs, 1H, NH), 7.26 (t, *J* = 7.6 Hz, 1H), 7.23–7.10 (m, 4H), 7.08 (d, *J* = 7.7 Hz, 1H), 6.86–6.74 (m, 3H), 4.74 (d, *J* = 15.3 Hz, 1H), 4.66 (d, *J* = 15.3 Hz, 1H), 4.38 (d, *J* = 12.6 Hz, 1H), 4.23 (d, *J* = 12.6 Hz, 1H), 3.40–3.30 (m, 3H), 3.25 (dd, *J*_1_ = 6.7 Hz, *J*_2_ = 9.4 Hz, 2H), 3.06 (d, *J* = 9.9 Hz, 1H), 2.44 (s, 3H), 2.17 (s, 6H). HRMS (ESI+) *m/z* calcd. for (C_21_H_20_N_4_O_2_S, M + H): 393.1379, found: (M + H): 393.1364.

**5″-Chloro-1′-methyl-1-benzyl-2-thioxodispiro[imidazolidine-4,3′-pyrrolidine-4′,3″-indoline]-2″,5-dione (4b)**

From **3b** (0.24 g, 0.65 mmol), sarcosine (0.23 g, 2.6 mmol) and paraformaldehyde, (0.08 g, 2.6 mmol) compound **4b** (0.21 g, 76%) was obtained as a white solid. M.p. 152–153 °C. ^1^H-NMR (400 MHz, DMSO-d_6_) δ: 10.54 (bs, 1H, NH), 7.34 (dd, *J*_1_ = 2.0 Hz, *J*_2_ = 8.3 Hz, 1H), 7.29 (d, *J* = 2.0 Hz, 1H), 7.25 (t, *J* = 7.5 Hz, 1H), 7.20–7.12 (m, 4H), 7.09 (d, *J* = 8.6 Hz, 1H), 6.75 (d, *J* = 6.5 Hz, 2H), 4.74 (d, *J* = 15.5 Hz, 1H), 4.67 (d, *J* = 15.5 Hz, 1H), 4.37 (d, *J* = 12.7 Hz, 1H), 4.20 (d, *J* = 12.7 Hz, 1H), 3.40–3.30 (m, 3H), 3.19 (t, *J* = 10.8 Hz, 2H), 3.07 (d, *J* = 9.7 Hz, 1H), 2.43 (s, 3H), 2.12 (s, 6H). HRMS (ESI+) *m/z* calcd. for (C_21_H_19_ClN_4_O_2_S, M + H): 427.0990, found: (M + H): 427.0976.

**1′-Methyl-1-allyl-2-thioxodispiro[imidazolidine-4,3′-pyrrolidine-4′,3″-indoline]-2″,5-dione (4c)**

From **3c** (0.19 g, 0.65 mmol), sarcosine (0.23 g, 2.6 mmol) and paraformaldehyde (0.08 g, 2.6 mmol), compound **4c** (0.15 g, 67%) was obtained as a white solid. M.p. 281–283 °C(decomp.). ^1^H-NMR (400 MHz, DMSO-d_6_) δ: 10.57 (s, 1H, NH), 10.44 (s, 1H, NH), 7.19 (d, *J* = 7.7 Hz, 1H), 7.15 (d, *J* = 8.1 Hz, 1H), 6.86 (t, *J* = 7.6 Hz, 1H), 6.79 (d, *J* = 7.7 Hz, 1H), 5.47 (m, 1H), 4.89 (d, *J* = 10.4 Hz, 1H), 4.59 (d, *J* = 17.4 Hz, 1H), 4.18–4.03 (m, 2H), 3.40 (d, *J* = 9.8 Hz, 1H), 3.32 (d, *J* = 9.8 Hz, 1H), 3.16 (d, *J* = 9.9 Hz, 1H), 3.05 (d, *J* = 10.0 Hz, 1H), 2.45 (c, 3H). HRMS (ESI+) *m/z* calcd. for (C_17_H_18_N_4_O_2_S, M + H): 343.1223, found: (M + H): 343.1207.

**5″-Chloro-1′-methyl-1-allyl-2-thioxodispiro[imidazolidine-4,3′-pyrrolidine-4′,3″-indoline]-2″,5-dione (4d)**

From **3d** (0.21 g, 0.65 mmol), sarcosine (0.23 g, 2.6 mmol) and paraformaldehyde (0.08 g, 2.6 mmol), compound **4d** (0.09 g, 38%) was obtained as a white solid. **M.p.** 141–142 °C. ^1^H-NMR (400 MHz, DMSO-d_6_) δ: 10.49 (bs, 1H, NH), 7.33 (dd, *J*_1_ = 1.9 Hz, *J*_2_ = 8.4 Hz, 1H), 7.26 (d, *J* = 1.7 Hz, 1H), 7.09 (d, *J* = 8.4 Hz, 1H), 5.47 (m, 1H), 4.88 (d, *J* = 10.2 Hz, 1H), 4.55 (d, *J* = 17.2 Hz, 1H), 4.38 (d, *J* = 12.6 Hz, 1H), 4.22 (d, *J* = 12.6 Hz, 1H), 4.20–4.02 (m, 2H), 3.30–3.21 (m, 3H), 3.04 (d, *J* = 10.0 Hz, 1H), 2.44 (s, 3H), 2.19 (s, 6H). HRMS (ESI+) *m/z* calcd. for (C_17_H_17_ClN_4_O_2_S, M + H): 377.0833, found: (M + H): 377.0822.

**1′-Methyl-1-(4-methoxyphenyl)-2-thioxodispiro[imidazolidine-4,3′-pyrrolidine-4′,3″-indoline]-2″,5-dione (4e)**

From **3e** (0.23 g, 0.65 mmol), sarcosine (0.23 g, 2.6 mmol) and paraformaldehyde (0.08 g, 2.6 mmol), compound **4e** (0.08 g, 28%) was obtained as a white solid. M.p. 155–156 °C. ^1^H-NMR (400 MHz, DMSO-d_6_) δ: 10.67–10.57 (m, 2H, NH), 7.26 (t, *J* = 7.5 Hz, 1H), 7.20 (d, *J* = 8.1 Hz, 2H), 7.12 (d, *J* = 7.5 Hz, 1H), 6.93 (t, *J* = 7.5 Hz, 1H), 6.86 (d, *J* = 7.7 Hz, 1H), 6.71 (d, *J* = 8.1 Hz, 2H), 3.49 (d, *J* = 9.9 Hz, 1H), 3.38 (m, 1H), 3.32 (m, 1H), 3.07 (d, *J* = 10.2 Hz, 1H), 2.47 (s, 3H), 2.31 (s, 3H). HRMS (ESI+) *m/z* calcd. for (C_21_H_20_N_4_O_3_S, M + H): 409.1328, found: (M + H): 409.1323.

**5″-Cchloro-1′-methyl-1-(4-methoxyphenyl)-2-thioxodispiro[imidazolidine-4,3′-pyrrolidine-4′,3″-indoline]-2″,5-dione (4f)**

From **3f** (0.25 g, 0.65 mmol), sarcosine (0.23 g, 2.6 mmol) and paraformaldehyde (0.08 g, 2.6 mmol), compound **4f** (0.14 g, 49%) was obtained as a white solid. M.p. 289–290 °C. ^1^H-NMR (400 MHz, DMSO-d_6_) δ: 10.79 (s, 1H, NH), 10.74 (s, 1H, NH), 7.34 (dd, *J*_1_ = 2.0 Hz, *J*_2_ = 8.3 Hz, 1H), 7.23 (d, *J* = 8.1 Hz, 2H), 7.12 (d, *J* = 1.8 Hz, 1H), 6.88 (d, *J* = 8.3 Hz, 1H), 6.74 (d, *J* = 8.1 Hz, 2H), 3.45–3.30 (m, 3H), 3.09 (d, *J* = 10.2 Hz, 1H), 2.47 (s, 3H), 2.32 (s, 3H). HRMS (ESI+) *m/z* calcd. for (C_21_H_19_ClN_4_O_3_S, M + H): 443.0939, found: (M + H): 443.0940.

**1′-Methyl-1-(4-ethoxyphenyl)-2-thioxodispiro[imidazolidine-4,3′-pyrrolidine-4′,3″-indoline]-2″,5-dione (4g)**

From **3g** (0.24 g, 0.65 mmol), sarcosine (0.23 g, 2.6 mmol) and paraformaldehyde (0.08 g, 2.6 mmol), compound **4g** (0.10 g, 35%) was obtained as a white solid. M.p. 240–241 °C. ^1^H-NMR (400 MHz, DMSO-d_6_) δ: 10.63 (s, 1H, NH), 10.60 (s, 1H, NH), 7.26 (t, *J* = 7.7 Hz, 1H), 7.13 (d, *J* = 7.5 Hz, 1H), 6.98–6.89 (m, 3H), 6.68 (d, *J* = 7.7 Hz, 1H),6.73 (d, *J* = 8.6 Hz, 2H), 4.03 (q, *J* = 7.0 Hz, 2H), 3.49 (d, *J* = 10.0 Hz, 1H), 3.40–3.28 (m, 2H),3.07 (d, *J* = 10.0 Hz, 1H), 2.47 (s, 3H), 1.32 (t, *J* = 7.0 Hz, 3H). HRMS (ESI+) *m/z* calcd. for (C_22_H_22_N_4_O_3_S, M + H): 423.1485, found: (M + H): 423.1480.

**5″-Chloro-1′-methyl-1-(4-ethoxyphenyl)-2-thioxodispiro[imidazolidine-4,3′-pyrrolidine-4′,3″-indoline]-2″,5-dione (4h)**

From **3h** (0.26 g, 0.65 mmol), sarcosine (0.23 g, 2.6 mmol) and paraformaldehyde (0.08 g, 2.6 mmol), compound **4h** (0.08 g, 27%) was obtained as a white solid. M.p. 268–271 °C. ^1^H-NMR (400 MHz, DMSO-d_6_) δ: 10.80–10.61 (bs, 2H, NH), 7.33 (dd, *J*_1_ = 1.6 Hz, *J*_2_ = 8.2 Hz, 1H), 7.13 (d, *J* = 1.6 Hz, 1H), 6.95 (d, *J* = 8.8 Hz, 2H),6.87 (d, *J* = 8.3 Hz, 1H), 6.76 (d, *J* = 8.4 Hz, 2H), 4.04 (q, *J* = 7.0 Hz, 2H), 3.45–3.34 (m, 3H),3.40–3.28 (m, 2H), 3.09 (d, *J* = 10.3 Hz, 1H), 2.47 (s, 3H), 1.32 (t, *J* = 7.0 Hz, 3H). HRMS (ESI+) *m/z* calcd. for (C_22_H_21_ClN_4_O_3_S, M + H): 457.1095, found: (M + H): 457.1090.

**1′-Methyl-1-(p-tolyl)-2-thioxodispiro[imidazolidine-4,3′-pyrrolidine-4′,3″-indoline]-2″,5-dione (4i)**

From **3i** (0.22 g, 0.65 mmol), sarcosine (0.23 g, 2.6 mmol) and paraformaldehyde (0.08 g, 2.6 mmol), compound **4i** (0.10 g, 39%) was obtained as a white solid. M.p. 155–157 °C. ^1^H-NMR (400 MHz, DMSO-d_6_) δ: 10.61 (bs, 1H, NH), 10.57 (bs, 1H, NH), 7.26 (dd, *J*_1_ = 1.0 Hz, *J*_2_ = 7.7 Hz, 1H), 7.14 (d, *J* = 7.2 Hz, 1H), 6.97–6.91 (m, 3H), 6.86 (d, *J* = 7.7 Hz, 1H), 6.75 (d, *J* = 8.8 Hz, 2H), 3.76 (s, 3H), 3.49 (d, *J* = 10.1 Hz, 1H), 3.37 (d, *J* = 10.1 Hz, 1H), 3.32 (m, 1H), 3.07 (d, *J* = 10.1 Hz, 1H), 2.47 (s, 3H). HRMS (ESI+) *m/z* calcd. for (C_21_H_20_N_4_O_2_S, M + H): 393.1379, found: (M + H): 393.1384.

**5″-Chloro-1′-methyl-1-(p-tolyl)-2-thioxodispiro[imidazolidine-4,3′-pyrrolidine-4′,3″-indoline]-2″,5-dione (4j)**

From **3j** (0.24 g, 0.65 mmol), sarcosine (0.23 g, 2.6 mmol) and paraformaldehyde (0.08 g, 2.6 mmol), compound **4j** (0.14 g, 50%) was obtained as a white solid. M.p. 159–160 °C. ^1^H-NMR (400 MHz, DMSO-d_6_) δ: 10.76 (s, 1H, NH), 10.69 (bs, 1H, NH), 7.33 (dd, *J*_1_ = 2.2 Hz, *J*_2_ = 8.3 Hz, 1H), 7.14 (d, *J* = 2.2 Hz, 1H), 6.99–6.94 (m, 2H), 6.87 (d, *J* = 8.3 Hz, 1H), 6.80–6.75 (m, 2H), 3.78 (s, 3H), 3.44–3.32 (m, 3H), 3.09 (d, *J* = 10.2 Hz, 1H), 2.47 (s, 3H). HRMS (ESI+) *m/z* calcd. for (C_21_H_19_ClN_4_O_2_S, M + H): 427.0990, found: (M + H): 427.0987.

**5″-Chloro-1′-methyl-1-(4-chlorophenyl)-2-thioxodispiro[imidazolidine-4,3′-pyrrolidine-4′,3″-indoline]-2″,5-dione (4k)**

From **3k** (0.23 g, 0.65 mmol), sarcosine (0.23 g, 2.6 mmol) and paraformaldehyde (0.08 g, 2.6 mmol), compound **4k** (0.13 g, 48%) was obtained as a white solid. M.p. 163–164 °C. ^1^H-NMR (400 MHz, DMSO-d_6_) δ: 10.72 (bs, 1H, NH), 10.62 (s, 1H, NH), 7.53–7.48 (m, 2H), 7.26 (td, *J*_1_ = 1.2 Hz, *J*_2_ = 7.7 Hz, 1H), 7.12 (d, *J* = 7.4 Hz, 1H), 6.93 (td, *J*_1_ = 0.9 Hz, *J*_2_ = 7.7 Hz, 1H), 6.91–6.87 (m, 2H), 6.86 (d, *J* = 7.9 Hz, 1H), 3.49 (d, *J* = 10.1 Hz, 1H), 3.39–3.33 (m, 2H), 3.08 (d, *J* = 10.0 Hz, 1H), 2.48 (s, 3H). HRMS (ESI+) *m/z* calcd. for (C_20_H_17_ClN_4_O_2_S, M + H): 413.0833, found: (M + H): 413.0829.

**5″-Chloro-1′-methyl-1-(4-chlorophenyl)-2-thioxodispiro[imidazolidine-4,3′-pyrrolidine-4′,3″-indoline]-2″,5-dione (4l)**

From **3l** (0.25 g, 0.65 mmol), sarcosine (0.23 g, 2.6 mmol) and paraformaldehyde (0.08 g, 2.6 mmol), compound **4l** (0.03 g, 10%) was obtained as a white solid. M.p. 239–240 °C. ^1^H-NMR (400 MHz, DMSO-d_6_) δ: 10.84 (bs, 1H, NH), 10.77 (s, 1H, NH), 7.55–7.50 (m, 2H), 7.26 (dd, *J*_1_ = 2.2 Hz, *J*_2_ = 8.3 Hz, 1H), 7.12 (d, *J* = 2.1 Hz, 1H), 6.94–6.89 (m, 2H), 6.87 (d, *J* = 8.3 Hz, 1H), 3.49 (d, *J* = 10.1 Hz, 1H), 3.44–3.38 (m, 2H), 3.33 (m, 1H), 3.10 (d, *J* = 10.4 Hz, 1H), 2.48 (s, 3H). HRMS (ESI+) *m/z* calcd. for (C_20_H_16_Cl_2_N_4_O_2_S, M + H): 447.0443, found: (M + H): 447.0433.

**1′-Methyl-1-(4-fluorophenyl)-2-thioxodispiro[imidazolidine-4,3′-pyrrolidine-4′,3″-indoline]-2″,5-dione (4m)**

From **3m** (0.22 g, 0.65 mmol), sarcosine (0.23 g, 2.6 mmol) and paraformaldehyde (0.08 g, 2.6 mmol), compound **4m** (0.06 g, 24%) was obtained as a white solid. M.p. 273–274 °C. ^1^H-NMR (400 MHz, DMSO-d_6_) δ: 10.91 (bs, 1H, NH), 10.79 (s, 1H, NH), 7.55 (t, *J* = 9.0 Hz, 1H), 7.43 (dd, *J*_1_ = 2.1 Hz, *J*_2_ = 8.3 Hz, 1H), 7.13–7.07 (m, 2H), 6.94 (m, 1H), 6.89 (d, *J* = 8.3 Hz, 1H), 3.45–3.37 (m, 2H), 3.32 (d, *J* = 10.2 Hz, 1H), 3.10 (d, *J* = 10.2 Hz, 1H), 2.48 (s, 3H). HRMS (ESI+) *m/z* calcd. for (C_20_H_17_FN_4_O_2_S, M + H): 397.1129, found: (M + H): 397.1115

**5″-Chloro-1′-methyl-1-(4-fluorophenyl)-2-thioxodispiro[imidazolidine-4,3′-pyrrolidine-4′,3″-indoline]-2″,5-dione (4n)**

From **3n** (0.24 g, 0.65 mmol), sarcosine (0.23 g, 2.6 mmol) and paraformaldehyde (0.08 g, 2.6 mmol), compound 4n (0.10 g, 35%) was obtained as a white solid. M.p. 273–274 °C. ^1^H-NMR (400 MHz, DMSO-d_6_) δ: 10.82 (bs, 1H, NH), 10.78 (bs, 1H, NH), 7.37–7.26 (m, 3H), 7.12 (s, 1H), 6.97–6.85 (m, 3H), 3.43–3.38 (m, 2H), 3.32 (d, *J* = 10.2 Hz, 1H), 3.10 (d, *J* = 10.2 Hz, 1H), 2.48 (s, 3H). HRMS (ESI+) *m/z* calcd. for (C_20_H_16_FClN_4_O_2_S, M + H): 431.0739, found: (M + H): 431.0760.

**1′-Methyl-1-(3-chlorobenzyl)-2-thioxodispiro[imidazolidine-4,3′-pyrrolidine-4′,3″-indoline]-2″,5-dione (4o)**

From **3o** (0.24 g, 0.65 mmol), sarcosine (0.23 g, 2.6 mmol) and paraformaldehyde (0.08 g, 2.6 mmol), compound **4o** (0.24 g, 87%) was obtained as a white solid. M.p. 273–274 °C. ^1^H-NMR (400 MHz, DMSO-d_6_) δ: 10.59 (bs, 1H, NH), 10.57 (bs, 1H, NH), 7.28 (d, *J* = 7.6 Hz, 1H), 7.23–7.11 (m, 2H), 7.05 (s, 1H), 7.02 (d, *J* = 7.6 Hz, 1H), 6.78 (d, *J* = 7.7 Hz, 1H), 6.74 (d, *J* = 7.6 Hz, 1H), 6.67 (t, *J* = 7.6 Hz, 1H), 4.76 (d, *J* = 15.5 Hz, 1H), 4.66 (d, *J* = 15.5 Hz, 1H), 3.39 (d, *J* = 10.1 Hz, 1H), 3.20 (d, *J* = 10.0 Hz, 1H), 3.05 (d, *J* = 10.0 Hz, 1H), 2.44 (s, 3H). HRMS (ESI+) *m/z* calcd. for (C_21_H_19_ClN_4_O_2_S, M + H): 427.0995, found: (M + H): 427.0981.

**5″-Chloro-1′-methyl-1-(3-chlorobenzyl)-2-thioxodispiro[imidazolidine-4,3′-pyrrolidine-4′,3″-indoline]-2″,5-dione (4p)**

From **3p** (0.26 g, 0.65 mmol), sarcosine (0.23 g, 2.6 mmol) and paraformaldehyde (0.08 g, 2.6 mmol), compound **4p** (0.18 g, 61%) was obtained as a white solid. M.p. 261–262 °C. ^1^H-NMR (400 MHz, DMSO-d_6_) δ: 10.71 (s, 1H, NH), 10.68 (bs, 1H, NH), 7.29–7.20 (m, 2H), 7.18 (t, *J* = 7.9 Hz, 1H), 7.12 (s, 1H), 6.98 (s, 1H), 6.80 (d, *J* = 8.3 Hz, 1H), 6.67 (d, *J* = 7.3 Hz, 1H), 4.77 (d, *J* = 15.7 Hz, 1H), 4.68 (d, *J* = 15.7 Hz, 1H), 3.30 (d, *J* = 11.0 Hz, 1H), 3.24 (d, *J* = 8.8 Hz, 2H), 3.07 (d, *J* = 9.9 Hz, 1H), 2.44 (s, 3H). HRMS (ESI+) *m/z* calcd. for (C_21_H_18_Cl_2_N_4_O_2_S, M + H): 461.0600, found: (M + H): 461.0610.

**1′-Methyl-1-(3-chloro-4-fluorophenyl)-2-thioxodispiro[imidazolidine-4,3′-pyrrolidine-4′,3″-indoline]-2″,5-dione(4q)**

From **3q** (0.24 g, 0.65 mmol), sarcosine (0.23 g, 2.6 mmol) and paraformaldehyde (0.08 g, 2.6 mmol), compound **4q** (0.21 g, 75%) was obtained as a white solid. M.p. 252–254 °C. ^1^H-NMR (400 MHz, DMSO-d_6_) δ: 10.81 (s, 1H, NH), 10.64 (s, 1H, NH), 7.51 (t, *J* = 8.9 Hz, 1H), 7.27 (t, *J* = 7.6 Hz, 1H), 7.12 (d, *J* = 7.3 Hz, 2H), 6.93 (t, *J* = 7.5 Hz, 1H), 6.89–6.81 (m, 3H), 3.47 (d, *J* = 10.2 Hz, 1H), 3.37 (d, *J* = 10.8 Hz, 2H), 3.08 (d, *J* = 10.2 Hz, 1H), 2.48 (s, 3H). HRMS (ESI+) *m/z* calcd. for (C_20_H_16_ClFN_4_O_2_S, M + H): 431.0739, found: (M + H): 431.0721.

**5″-Chloro-1′-methyl-1-(3-chloro-4-fluorophenyl)-2-thioxodispiro[imidazolidine-4,3′-pyrrolidine-4′,3″-indoline]-2″,5-dione (4r)**

From **3r** (0.27 g, 0.65 mmol), sarcosine (0.23 g, 2.6 mmol) and paraformaldehyde (0.08 g, 2.6 mmol), compound **4r** (0.10 g, 33%) was obtained as a white solid. M.p. 177–180 °C. ^1^H-NMR (400 MHz, DMSO-d_6_) δ: 10.91 (bs, 1H, NH), 10.79 (s, 1H, NH), 7.55 (t, *J* = 9.0 Hz, 1H), 7.34 (dd, *J*_1_ = 2.1 Hz, *J*_2_ = 8.3 Hz, 1H), 7.13–7.07 (m, 2H), 6.94 (m, 1H), 6.89 (d, *J* = 8.3 Hz,1H), 3.45–3.37 (m, 2H), 3.32 (d, *J* = 10.2 Hz, 1H), 3.10 (d, *J* = 10.2 Hz, 1H), 2.48 (s, 3H). HRMS (ESI+) *m/z* calcd. for (C_20_H_15_Cl_2_FN_4_O_2_S, M + H): 465.0350, found: (M + H): 465.0341.

**1′-Methyl-1-cyclopropyl-2-thioxodispiro[imidazolidine-4,3′-pyrrolidine-4′,3″-indoline]-2″,5-dione (4s)**

From **3s** (0.19 g, 0.65 mmol), sarcosine (0.23 g, 2.6 mmol) and paraformaldehyde (0.08 g, 2.6 mmol), compound **4s** (0.12 g, 54%) was obtained as a white solid. M.p. 272–273 °C (decomp.). ^1^H-NMR (400 MHz, DMSO-d_6_) δ: 10.50 (s, 1H, NH), 10.28 (s, 1H, NH), 7.19 (t, *J* = 7.7 Hz, 1H), 7.11 (d, *J* = 7.5 Hz, 1H), 6.89 (t, *J* = 7.6 Hz, 1H), 6.77 (d, *J* = 7.7 Hz, 1H), 3.30 (d, *J* = 10.0 Hz, 1H), 3.25 (d, *J* = 10.0 Hz, 1H), 3.14 (d, *J* = 10.0 Hz, 1H), 3.01 (d, *J* = 10.0 Hz, 1H), 2.45 (m, 1H), 2.43 (s, 3H), 0.82–0.70 (m, 2H), 0.62 (m, 1H), 0.11 (m, 1H). HRMS (ESI+) *m/z* calcd. for (C_17_H_18_N_4_O_2_S, M + H): 343.1223, found: (M + H): 343.1241.

**5″-Chloro-1′-methyl-1-cyclopropyl-2-thioxodispiro[imidazolidine-4,3′-pyrrolidine-4′,3″-indoline]-2″,5-dione (4t)**

From **3t** (0.21 g, 0.65 mmol), sarcosine (0.23 g, 2.6 mmol) and paraformaldehyde (0.08 g, 2.6 mmol), compound **4t** (0.06 g, 25%) was obtained as a white solid. M.p. 275–276 °C. ^1^H-NMR (400 MHz, DMSO-d_6_) δ: 10.65 (s, 1H, NH), 10.41 (s, 1H, NH), 7.26 (m, 1H), 7.10 (m, 1H), 6.79 (dd, *J*_1_ = 2.1 Hz, *J*_2_ = 8.3 Hz, 1H), 3.26–3.16 (m, 3H), 3.02 (d, *J* = 10.0 Hz, 1H), 2.43 (s, 3H), 0.89–0.74 (m, 2H), 0.59 (m, 1H), 0.11 (m, 1H). HRMS (ESI+) *m/z* calcd. for (C_17_H_17_ClN_4_O_2_S, M + H): 377.0834, found: (M + H): 377.0851.

**1′-Methyl-1-(3-morpholinopropyl)-2-thioxodispiro[imidazolidine-4,3′-pyrrolidine-4′,3″-indoline]-2″,5-dione (4u)**

From **3u** (0.24 g, 0.65 mmol), sarcosine (0.23 g, 2.6 mmol) and paraformaldehyde (0.08 g, 2.6 mmol), compound **4u** (0.08 g, 25%) was obtained as a white solid. M.p. 216–217 °C. ^1^H-NMR (400 MHz, DMSO-d_6_) δ: 10.55 (s, 1H, NH), 10.39 (s, 1H, NH), 7.22–7.14 (m, 2H), 6.88 (t, *J* = 7.6 Hz, 1H), 6.78 (d, *J* = 7.8 Hz, 1H), 3.61–3.43 (m, 6H), 3.38 (d, *J* = 7.1 Hz, 1H), 3.31 (d, *J* = 10.6 Hz, 1H), 3.15 (d, *J* = 10.0 Hz, 1H), 3.04 (d, *J* = 10.0 Hz, 1H), 2.44 (s, 3H), 2.28–2.18 (m, 4H), 2.05 (t, *J* = 6.7 Hz, 2H), 1.42–1.33 (m, 2H). HRMS (ESI+) *m/z* calcd. for (C_21_H_27_N_5_O_3_S, M + H): 430.1907, found: (M + H): 430.1904.

**5″-Chloro-1′-methyl-1-(3-morpholinopropyl)-2-thioxodispiro[imidazolidine-4,3′-pyrrolidine-4′,3″-indoline]-2″,5-dione (4v)**

From **3v** (0.26 g, 0.65 mmol), sarcosine (0.23 g, 2.6 mmol) and paraformaldehyde (0.08 g, 2.6 mmol), compound **4v** (0.05 g, 15%) was obtained as a white solid. M.p. 224–225 °C. ^1^H-NMR (400 MHz, DMSO-d_6_) δ: 10.69 (s, 1H, NH), 10.49 (s, 1H, NH), 7.25 (dd, *J*_1_ = 1.8 Hz, *J*_2_ = 8.5 Hz, 1H), 7.19 (s, 1H), 6.80 (d, *J* = 8.1 Hz, 1H), 3.64–3.46 (m, 6H), 3.30–3.18 (m, 3H), 3.06 (d, *J* = 9.9 Hz, 1H), 2.43 (s, 3H), 2.28–2.19 (m, 4H), 2.10–2.03 (m, 2H), 1.44–1.28 (m, 2H). HRMS (ESI+) *m/z* calcd. for (C_21_H_26_ClN_5_O_3_S, M + H): 464.1517, found: (M + H): 464.1519.

**1′-Methyl-1-phenyl-2-thioxodispiro[imidazolidine-4,3′-pyrrolidine-4′,3″-indoline]-2″,5-dione (4w)**

From **3w** (0.21 g, 0.65 mmol), sarcosine (0.23 g, 2.6 mmol) and paraformaldehyde (0.08 g, 2.6 mmol), compound **4w** (0.07 g, 27%) was obtained as a white solid. M.p. 273–274 °C. ^1^H-NMR (400 MHz, DMSO-d_6_) δ: 10.65 (bs, 1H, NH), 10.63 (s, 1H, NH), 7.45–7.36 (m, 3H), 7.27 (t, *J* = 7.7 Hz, 1H), 7.15 (d, *J* = 7.5 Hz, 1H), 6.94 (t, *J* = 7.6 Hz, 1H), 6.89–6.82 (m, 3H), 3.51 (d, *J* = 10.0 Hz, 1H), 3.37 (d, *J* = 10.0 Hz, 2H), 3.08 (d, *J* = 10.2 Hz, 1H), 2.48 (s, 3H). HRMS (ESI+) *m/z* calcd. for (C_20_H_18_N_4_O_2_S, M + H): 379.1223, found: (M + H): 379.1236.

**5″-Chloro-1′-methyl-1-phenyl-2-thioxodispiro[imidazolidine-4,3′-pyrrolidine-4′,3″-indoline]-2″,5-dione (4x)**

From **three times** (0.23 g, 0.65 mmol), sarcosine (0.23 g, 2.6 mmol) and paraformaldehyde (0.08 g, 2.6 mmol), compound four times (0.14 g, 49%) was obtained as a white solid. M.p. 268–269 °C. ^1^H-NMR (400 MHz, DMSO-d_6_) δ: 10.79 (bs, 1H, NH), 10.78 (bs, 1H, NH), 7.47–7.39 (m, 3H), 7.34 (dd, *J*_1_ = 1.6 Hz, *J*_2_ = 8.3 Hz, 1H), 7.14 (s, 1H), 6.91–6.85 (m, 3H), 3.43 (d, *J* = 10.2 Hz, 1H), 3.39 (d, *J* = 10.2 Hz, 1H), 3.34 (d, *J* = 10.2 Hz, 1H), 3.10 (d, *J* = 10.2 Hz, 1H), 2.48 (s, 3H). HRMS (ESI+) *m/z* calcd. for (C_20_H_17_ClN_4_O_2_S, M + H): 413.0834, found: (M + H): 413.0850.

#### 3.2.4. General Procedure for the Synthesis of Dispiroindolinones **6**

Corresponding 5-indolidene-2-selenoxoimidazolidin **5** (1 equiv) and sarcosine (4 equiv) were dissolved in toluene and the mixture heated to a boiling point. After that paraformaldehyde (4 equiv) was added. The resulting mixture was refluxed for 5–8 hours (TLC control). After the reaction was completed, the solvent was evaporated in vacuo. The product was then purified using column chromatography (silica gel 60, 0.04–0.063 mm/230–400 mesh, CHCl_3_:MeOH/50:1) to afford products as a white solid. This solid was washed with cold methanol to yield corresponding dispirooxindole as light brown crystalline solid.

**5″-Chloro-1-(3-chloro-4-fluorophenyl)-1′-methyl-2-selenoxodispiro[imidazolidine-4,3′-pyrrolidine-4′,3″-indoline]-2″,5-dione (6r)**

From **5r** (0.30 g, 0.65 mmol), sarcosine (0.23 g, 2.6 mmol) and paraformaldehyde (0.08 g, 2.6 mmol), compound **6r** (0.19 g, 57%) was obtained as light brown solid. M.p. 193–194 °C (decomp.). ^1^H-NMR (400 MHz, DMSO-d_6_) δ: 11.62 (s, 1H, NH), 10.81 (s, 1H, NH), 7.57 (t, *J* = 9.0 Hz, 1H), 7.36 (dd, *J*_1_ = 1.8 Hz, *J*_2_ = 8.3 Hz, 1H), 7.12 (m, 1H), 7.10 (m, 1H), 6.96 (m, 1H), 6.90 (d, *J* = 8.3 Hz, 1H), 3.43 (s, 2H), 3.32 (m, 1H), 3.12 (d, *J* = 10.2 Hz, 1H), 2.49 (s, 3H). HRMS (ESI+) *m/z* calcd. for (C_20_H_17_ClN_4_O_2_S, M + H): 512.9800, found: (M + H): 513.0050.

**5″-Chloro-1-(4-methoxyphenyl)-1′-methyl-2-selenoxodispiro[imidazolidine-4,3′-pyrrolidine-4′,3″-indoline]-2″,5-dione (6f)**

From **5f** (0.28 g, 0.65 mmol), sarcosine (0.23 g, 2.6 mmol) and paraformaldehyde (0.08 g, 2.6 mmol), compound **6f** (0.19 g, 71%) was obtained as a white solid. M.p. 284–285 °C (decomp.).^1^H-NMR (400 MHz, DMSO-d_6_) δ: 11.41 (bs, 1H, NH), 10.79 (s, 1H, NH), 7.33 (dd, *J*_1_ = 1.9 Hz, *J*_2_ = 8.3 Hz, 1H), 7.13 (d, *J* = 1.9 Hz, 1H), 6.96 (d, *J* = 8.7 Hz, 2H), 6.87 (d, *J* = 8.3 Hz, 1H), 6.78 (d, *J* = 8.6 Hz, 2H), 3.77 (s, 3H), 3.45–3.37 (m, 2H), 3.31 (m, 1H), 3.10 (d, *J* = 10.2 Hz, 1H), 2.48 (s, 3H). HRMS (ESI+) *m/z* calcd. for (C_21_H20ClN_4_O_3_Se, M + H): 491.0384, found: (M + H): 491.0385.

#### 3.2.5. General Procedure for the Synthesis of 5-Substituted Hydantoins **8**

Corresponding 5-substituted-2-thiohydantoin **4** (1 equiv) was added to solution of the potassium hydroxide (1.05 equiv) in EtOH at room temperature (~4 mL EtOH for 100 mg of **3**). After that, MeI (1.5 eq) was added and the reaction mixture was stirred at room temperature overnight. Then EtOH:HCl conc.(1:1) was added to the reaction (~4 mL EtOH for 100 mg of **3**) and refluxed for 2 hours. Further, the reaction cooled to room temperature and formed precipitate was filtered off, washed with ethanol and dried in air. All compounds were obtained as red crystalline powders.

**(Z)-5-Chloro-3-(1-(4-methoxyphenyl)-5-oxo-2-thioxoimidazolidin-4-ylidene)indolin-2-one (8f)**

From **3f** (0.116 g, 0.30 mmol), KOH (0.018 g, 0.32 mmol) and MeI (0.064 g, 0.45 mmol), compound **8f** (0.089 g, 80%) was obtained as a red solid. M.p. > 300 °C. ^1^H-NMR (400 MHz, DMSO-d_6_) δ: 11.08 (s, 1H, NH), 11.06 (s, 1H, NH), 8.59 (d, *J* = 2.0 Hz, 1H), 7.95 (s, 1H, Ar), 7.39 (d, *J* = 8.8 Hz, 2H), 7.33 (dd, *J*_1_= 2.2 Hz, *J*_2_ = 8.4 Hz, 1H), 7.08 (d, *J* = 9.0 Hz, 2H), 6.94 (d, *J* = 8.4 Hz, 1H), 3.82 (s, 3H). HRMS (ESI+) *m/z* calcd. for (C_18_H_12_ClN_3_O_4_ M + H): 370.0595, found: (M + H): 370.0588.

**(Z)-5-Chloro-3-(1-(4-ethoxyphenyl)-5-oxo-2-thioxoimidazolidin-4-ylidene)indolin-2-one (8h)**

From **3h** (0.120 g, 0.30 mmol), KOH (0.018 g, 0.32 mmol) and MeI (0.064 g, 0.45 mmol), compound 8h (0.097 g, 84%) was obtained as a white solid. M.p. > 300 °C. ^1^H-NMR (400 MHz, DMSO-d_6_) δ: 11.66 (s, 1H, NH), 11.24 (s, 1H, NH), 8.54 (m, 1H), 7.39–7.70 (m, 3H), 7.09–7.04 (m, 2H), 6.94 (m, 1H) 4.08 (q, *J* = 6.9 Hz, 2H), 1.36 (t, *J* = 6.9 Hz, 3H). HRMS (ESI+) *m/z* calcd. for (C_19_H_14_ClN_3_O_4_, M + H): 084.0751, found: (M + H): 413.0781.

**(Z)-5-Chloro-3-(1-(3-chloro-4-fluorophenyl)-5-oxo-2-thioxoimidazolidin-4-ylidene)indolin-2-one (8r)**

From **3r** (0.122 g, 0.30 mmol), KOH (0.018 g, 0.32 mmol) and MeI (0.064 g, 0.45 mmol), compound 8r (0.093 g, 79%) was obtained as a white solid. M.p. > 300 °C. ^1^H-NMR (400 MHz, DMSO-d_6_) δ: 11.22 (s, 1H, NH), 11.10 (s, 1H, NH), 8.54 (m, 1H), 7.77 (dd, *J*_1_ = 2.2 Hz, *J*_2_ = 6.7 Hz, 1H), 7.64 (m, 1H), 7.54 (m, 1H), 7.36 (m, 1H), 6.95 (t, *J* = 9.0 Hz, 1H). HRMS (ESI+) *m/z* calcd. for (C_17_H_9_Cl_2_FN_3_O_3_, M + H): 392.0005, found: (M + H): 491.0998.

#### 3.2.6. General Procedure for the Synthesis of Dispiroindolinones **9**

Corresponding imidazolidin **8** (1 equiv) and sarcosine (4 equiv) were dissolved in toluene and the mixture heated to a boiling point. After that, paraformaldehyde (4 equiv) was added. The resulting mixture was refluxed for 5–8 hours (TLC control). After the reaction was completed, the solvent was evaporated in vacuo. The product was then purified using column chromatography (silica gel 60, 0.04–0.063 mm/230–400 mesh, CHCl_3_:MeOH/50:1) to afford products as a white solid. This solid was washed with acetone to yield corresponding dispirooxindole as a white crystalline solid.

**5″-Chloro-1-(4-methoxyphenyl)-1′-methyldispiro[imidazolidine-4,3′-pyrrolidine-4′,3″-indoline]-2,2″,5-trione (9f)**

From **8f** (0.074 g, 0.20 mmol), sarcosine (0.071 g, 0.80 mmol) and paraformaldehyde (0.026 g, 0.80 mmol), compound **9f** (0.061 g, 72%) was obtained as a white solid. M.p. 295–296 °C. ^1^H-NMR (400 MHz, DMSO-d_6_) δ: 10.74 (s, 1H NH), 8.80 (s, 1H, NH), 7.33 (dd, *J*_1_ = 2.2 Hz, *J*_2_ = 8.3 Hz, 1H), 7.17 (d, *J* = 2.0 Hz, 1H), 6.98 (d, *J* = 9.0 Hz, 2H), 6.90 (d, *J* = 8.9 Hz, 2H), 6.87 (m, 1H), 3.44–3.36 (m, 2H), 3.31 (d, *J* = 10.2 Hz, 1H), 3.08 (d, *J* = 10.3 Hz, 1H), 2.47 (s, 3H). HRMS (ESI+) *m/z* calcd. for (C_21_H_19_ClN_4_O_4_, M + H): 427.1173, found: (M + H): 427.1177.

**5″-Chloro-1-(4-ethoxyphenyl)-1′-methyldispiro[imidazolidine-4,3′-pyrrolidine-4′,3″-indoline]-2,2″,5-trione (9f)**

From **8h** (0.076 g, 0.20 mmol), sarcosine (0.071 g, 0.80 mmol) and paraformaldehyde (0.026 g, 0.80 mmol), compound **9h** (0.055 g, 72%) was obtained as a white solid. M.p. 273–274 °C. ^1^H-NMR (400 MHz, DMSO-d_6_) δ: 10.74 (s, 1H, NH), 8.80 (s, 1H, NH), 7.32 (m, 1H), 7.17 (s, 1H), 7.00–6.92 (m, 2H), 6.92–6.82 (m, 3H), 4.08–3.98 (m, 2H), 3.45–3.36 (m, 2H), 3.29 (m, 1H), 3.07 (m, 1H), 2.46 (s, 3H), 1.37–1.27 (m, 3H). HRMS (ESI+) *m/z* calcd. for (C_22_H_21_ClN_4_O_4_ M + H): 441.1330, found: (M + H): 441.1295.

**5″-Chloro-1-(3-chloro-4-fluorophenyl)-1′-methyldispiro[imidazolidine-4,3′-pyrrolidine-4′,3″-indoline]-2,2″,5-trione (9r)**

From **8r** (0.078 g, 0.20 mmol), sarcosine (0.071 g, 0.80 mmol) and paraformaldehyde (0.026 g, 0.80 mmol), compound **9r** (0.067 g, 75%) was obtained as a white solid. M.p. 197–198 °C. ^1^H-NMR (400 MHz, DMSO-d_6_) δ: 10.75 (s, 1H, NH), 8.99 (s, 1H, NH), 7.55 (t, *J* = 9.0 Hz, 1H), 7.33 (dd, *J*_1_ = 2.2 Hz, *J*_2_ = 8.3 Hz, 1H), 7.24 (dd, *J*_1_ = 2.5 Hz, *J*_2_ = 6.7 Hz, 1H), 7.15 (d, *J* = 2.0 Hz, 1H), 7.06 (m, 1H), 6.87 (d, *J* = 8.3 Hz, 1H), 3.41–3.34 (m, 3H), 3.08 (d, *J* = 10.2 Hz, 1H), 2.46 (s, 3H). HRMS (ESI+) *m/z* calcd. for (C_20_H_16_Cl_2_FN_4_O_3_, M + H): 449.0578, found: (M + H): 449.0589.

### 3.3. Biological Evaluation

MTT test. The MTT assay was carried out according to [35] with few modifications; 3000 Cells (for HEK293T, A549 and MCF7cell lines) or 4000 cells (for VA13 cell line) were seeded in each well of a 96-well plate. After 20 h incubation, the tested compounds diluted in culture medium were added to the cells and incubated 72 h at 37 °C under CO_2_ (5%) atmosphere. Assays were performed in triplicates. The MTT (3-[4,5-dimethylthiazol-2-yl]-2,5 diphenyl-tetrazolium bromide) reagent was then added to the cells up to final concentration of 0.5 g/L (10X stock solution in PBS was used) and incubated for 2 h at 37 °C (5% CO_2_). The MTT solution was then discarded and 140 µL of DMSO was added. The plates were swayed on a shaker (60 rpm) to solubilize the formazan. The absorbance was measured using a microplate reader at a wavelength of 565 nm. The analysis of cytotoxicity and the estimation of IC50 values were carried out with the built-in functions in the GraphPad Prism program (GraphPad Software, Inc., San Diego, CA) P53 activation.

The β-galactosidase reporter construction equipped with the p53 promotor frame [46] was used to assess the p53 expression level in p53wt A549 cell line. The compounds were tested in the concentration range of 0.5–120 μM with triple dilution steps. The incubation time was 24 h. To take into account the toxic effect of the molecules, the output signal was normalized considering the number of the cells estimated by MTT test with the same incubation tome (24 h). The output was statistically significant if the background signal was exceeded two or more times.

## 4. Conclusions

In the present study, a series of novel dispiro-oxindole derivatives of 2-chaicogen-imidazol-4-ones has been described. The synthesized molecules have key 3D-pharmacophore features essential for binding into the major MDM2 pocket as it has been predicted during the molecular docking study. However, these compounds have an alternative binding mode in contrast to other MDM2 inhibitors, therefore, they should be cautiously regarded as having this mechanism of action. MTT test with different cell lines, including p53 positive and negative, has not provided unambiguous results on the mechanism of action for this series although some signs of p53 activation have been observed. Nevertheless, the most active compounds from this series show fairly good cytotoxicity values (2.2–9.8 μM) on various cell lines in the MTT test, which makes them promising for further optimization and research. 

## Figures and Tables

**Table 1:** Cytotoxicity of dispiro-oxindoles **4**, **6**, **9** against different cell lines (MTT test).

Compound	Cell Lines							
	A549	MCF7	VA13	Hek293T	HCT^+/+^	HCT^−/−^	LNCaP	PC3
	CC_50_, μM							
**4a**	20.3 ± 3.7	31.3 ± 6.7	16.1 ± 2.5	9.8 ± 1.6	66.2 ± 11.74	40.11 ± 19.11	12.5 ± 2.1	30.1 ± 9.1
**4b**	9.1 ± 2.2	24.3 ± 2.3	21.4 ± 1.9	9.5 ± 1.7	60.73 ± 13.75	30.01 ± 15.21	10.3 ± 1.1	49.0 ± 12.7
**4c**	45.9 ± 13.4	na	na	41.8 ± 12.4	na	14.33 ± 6.65	9.8 ± 3.3	53.1 ± 15.8
**4d**	32.1 ± 6.7	68.4 ± 8.8	30.8 ± 4.5	11.5 ± 3	57.73 ± 18.41	40.11 ± 5.23	20.0 ± 3.6	50.0 ± 9.1
**4e**	18.9 ± 3.1	22.8 ± 5.6	13.2 ± 2.3	6.5 ± 0.9	27.9 ± 6.94	36.0 ± 12.55	3.45 ± 0.45	na
**4f**	2.8 ± 0.4	2.3 ± 0.5	2.3 ± 0.4	1.1 ± 0.1	1.95 ± 0.43	2.35 ± 0.95	4.5 ± 0.32	12.6 ± 2.3
**6f**	15.8 ± 1.5	20.4 ± 3.6	12.5 ± 1.5	14 ± 1.5	–	–	*–*	*–*
**4g**	38.2 ± 11.5	75.2 ± 29.3	73.6 ± 23.4	30.2 ± 8.2	na	na	na	na
**4h**	6.2 ± 2.1	8.5 ± 1.2	6.3 ± 1.1	6.7 ± 0.9	na	na	na	na
**4i**	na	88.2 ± 32.2	na	na	na	na	na	na
**4j**	62 ± 8.3	13.7 ± 2.1	27.2 ± 5.3	17.4 ± 2	na	na	na	na
**4k**	67.5 ± 6.9	27.9 ± 6.9	59.1 ± 7.6	69 ± 3.7	52.7 ± 11.4	40.9 ± 20.5	5.2 ± 0.91	7.8 ± 1.4
**4l**	37 ± 6.5	19.1 ± 4.1	30.4 ± 3	34 ± 3.4	42.5 ± 9.1	57.11 ± 5.76	4.4 ± 0.67	2.2 ± 0.7
**4m**	na	105.9 ± 43.1	171.0 ± 15.8	na	23.3 ± 3.3	42.4 ± 4.7	3.4 ± 0.7	1.93 ± 0.91
**4n**	na	–	–	53.0± 9.5	na	na	na	na
**4o**	53.4 ± 11.2	23.2 ± 3.6	36.3 ± 3.2	48 ± 5.3	na	na	na	na
**4p**	11.3 ± 2.4	6.6 ± 1	12.3 ± 1.6	18.4 ± 1.7	34.2 ± 5.7	na	4.1 ± 0.1	2.2 ± 1.4
**4q**	na	62.2 ± 27.7	57.9 ± 18.3	69.1 ± 21	na	na	na	na
**4r**	14.1 ± 1.8	14.1 ± 1.2	13.9 ± 1.5	11.1 ± 1.3	na	na	na	na
**6r**	26.5 ± 5.4	–	–	11.9 ± 0.9	–	–	11.8 ± 1.5	>50
**9r**	35.3 ± 17.7	–	–	15.1 ± 1.2	–	–	19.6 ± 1.8	>50
**4s**	na	na	na	na	na	na	na	na
**4t**	na	na	na	na	na	na	na	na
**4u**	na	na	na	na	20.8 ± 4.8	na	3.4 ± 0.6	53.6 ± 17.3
**4v**	na	–	–	103.2 ± 27.7	na	na	na	na
**4w**	na	57.9 ± 17.1	94.1 ± 19.7	75.1 ± 8.6	54.7 ± 9.5	na	9.8 ± 1.8	52.2 ± 11.6
**4x**	na	–	–	na	na	na	na	na
Etoposide	0.3 ± 0.1	2.6 ± 0.9	1.1 ± 0.2	0.3 ± 0.1	0.43 ± 0.12	0.85 ± 0.22	–	–
Nutlin–3	–	–	–	–	3.3 ± 0.13	35.12 ± 2.65	–	–

na—not active (CC_50_>100 μM); “–“—not tested. Etoposide [39] as a topoisomerase poison, and nutlin-3 [19] as P53/MDM2 interaction inhibitor, were also tested on some cell lines as positive control.

## Data Availability

The data presented in this study are openly available in file mail.ru repository at https://cloud.mail.ru/public/svf7/4u5R7wxqq, accessed on 20 November 2021.

## Supplementary Materials

The following are available online. Figure S1: NMR spectra of synthesized compounds.

## Sample Availability

Samples of the compounds are not available from the authors.
