# Supplementary material for: Synthesis and Biological Evaluation of S-, O- and Se-Containing Dispirooxindoles
Source: Molecules. 2021 Dec 16;26(24):7645. doi: 10.3390/molecules26247645 (PMC8703884; doi:10.3390/molecules26247645)

Synthesis and biological evaluation of S-, O- and Se-containing dispirooxindoles

Maksim Kukushkin[1,2], Vladimir Novotortsev[1], Vadim Filatov[1], Yan Ivanenkov[1-3], Dmitry Skvortsov[1], Mark Veselov[3], Radik Shafikov[1], Anna Moiseeva[1], Nikolay Zyk[1], Alexander Majouga[1,4] and Elena Beloglazkina[1,]*

[1] Lomonosov Moscow State University, Leninskie gory, 1/3, GSP-1, Moscow, 119991, Russian Federation

[2] National University of Science and Technology MISiS, Moscow, 119049, Russian Federation

[3] Moscow Institute of Physics and Technology (MIPT), Institutski Pereulok 9, Dolgoprudny,141701, Russian Federation

[4] Dmitry Mendeleev University of Chemical Technology of Russia, Miusskaya sq. 9, Moscow, 125047, Russian Federation

Correspondence: beloglazki@mail.ru

Supplementary Information

^{1}HNMR spectra of compounds 1-9

Ethyl 2-(3-benzylthioureido)acetate (1a)

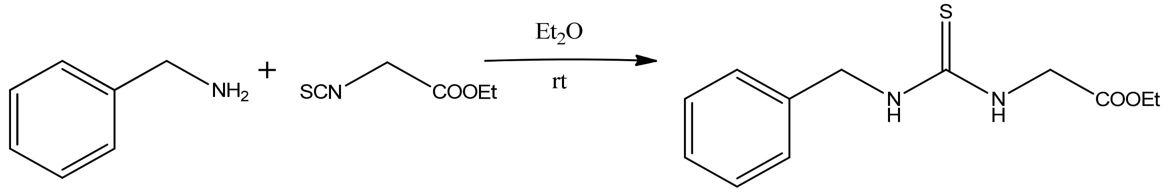

Chemical Formula: $C_{12}H_{16}N_2O_2S$
Molecular Weight: 252,3326

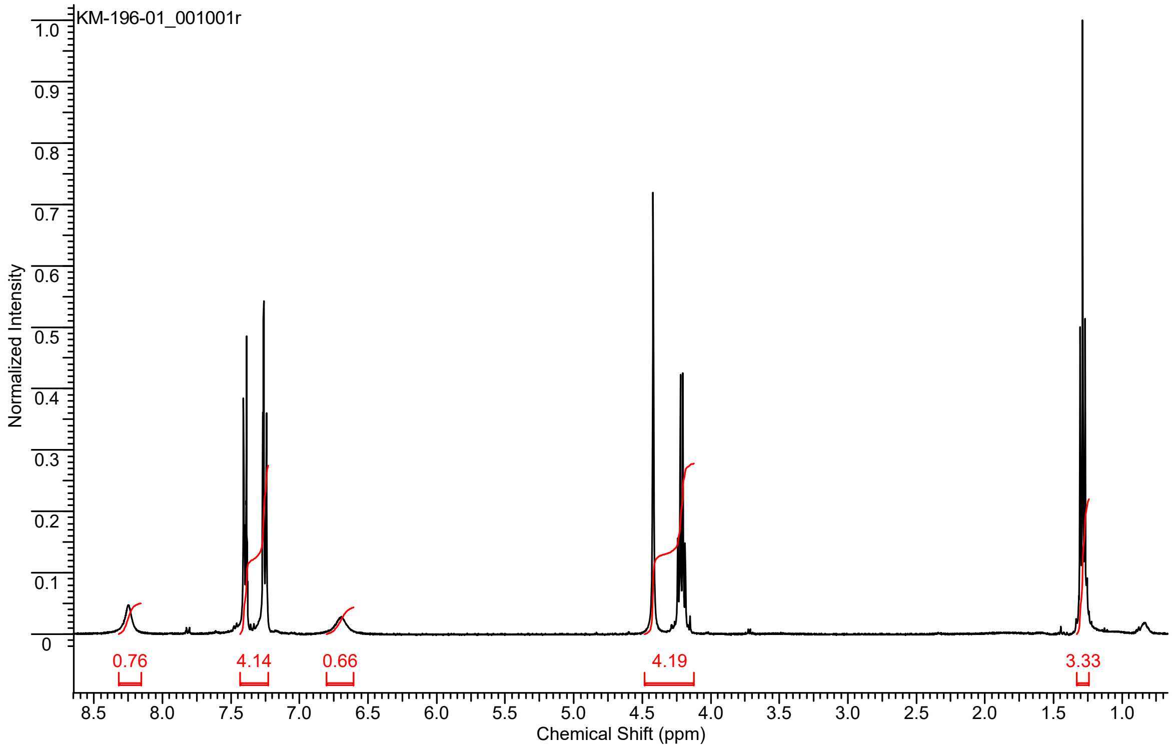

Ethyl 2-(3-allylthioureido)acetate (1b)

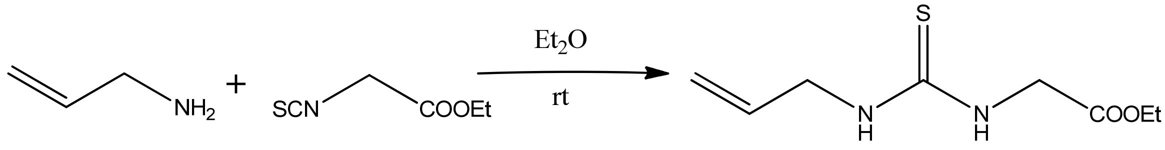

Chemical Formula: $C_8H_{14}N_2O_2S$
Molecular Weight: 202,2740

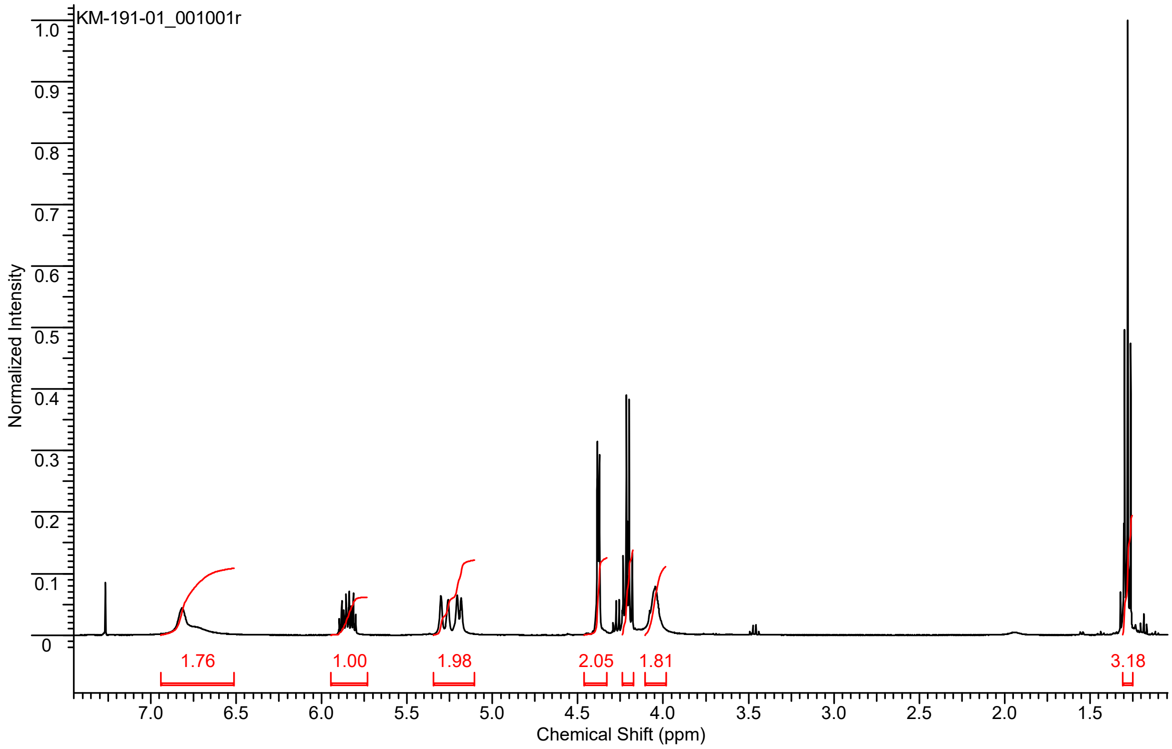

Ethyl 2-(3-(4-methoxyphenyl)thioureido)acetate (1c)

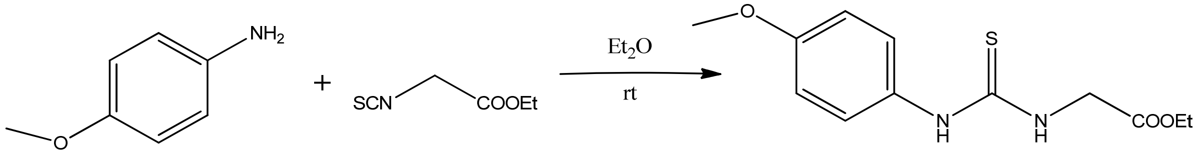

Chemical Formula: $C_{12}H_{16}N_2O_3S$
Molecular Weight: 268,3320

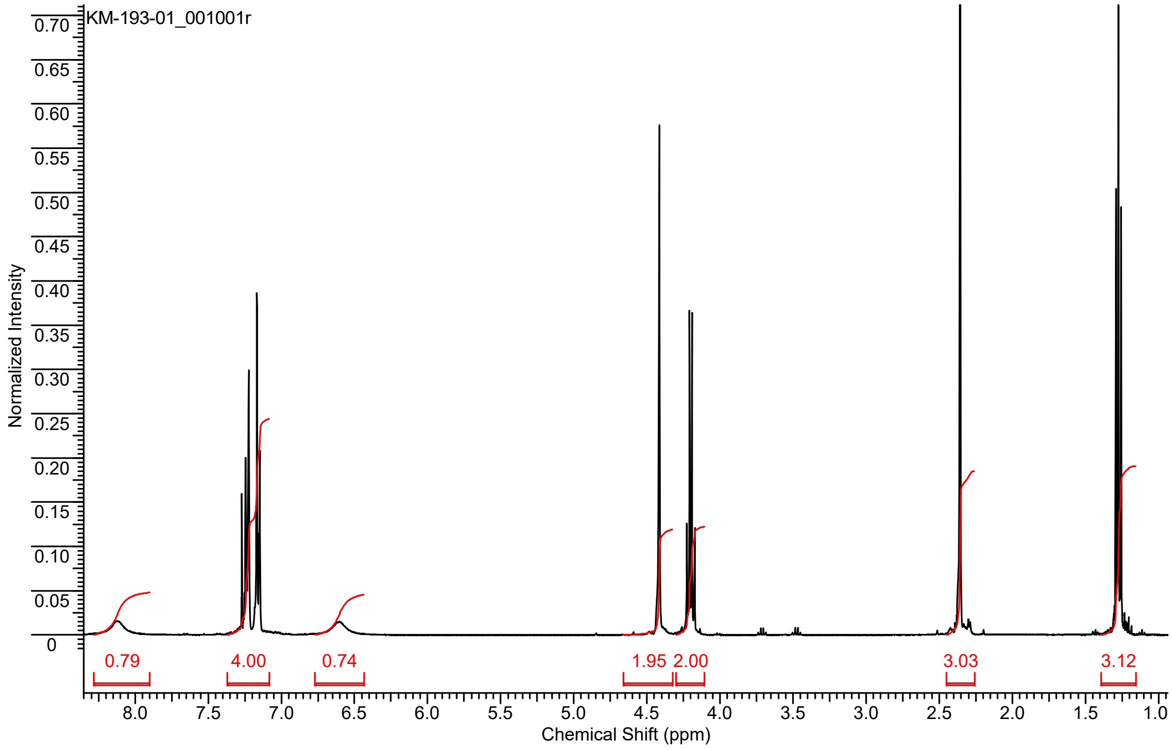

Ethyl 2-(3-(4-ethoxyphenyl)thioureido)acetate (1d)

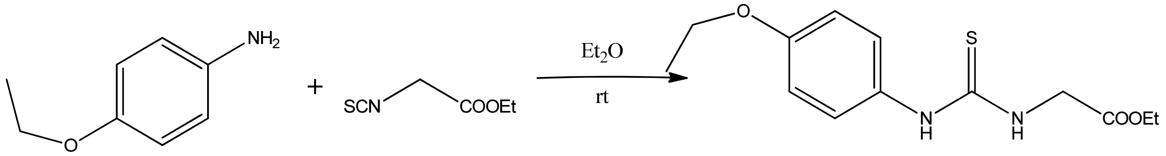

Chemical Formula: $C_{13}H_{18}N_2O_3S$
Molecular Weight: 282,3586

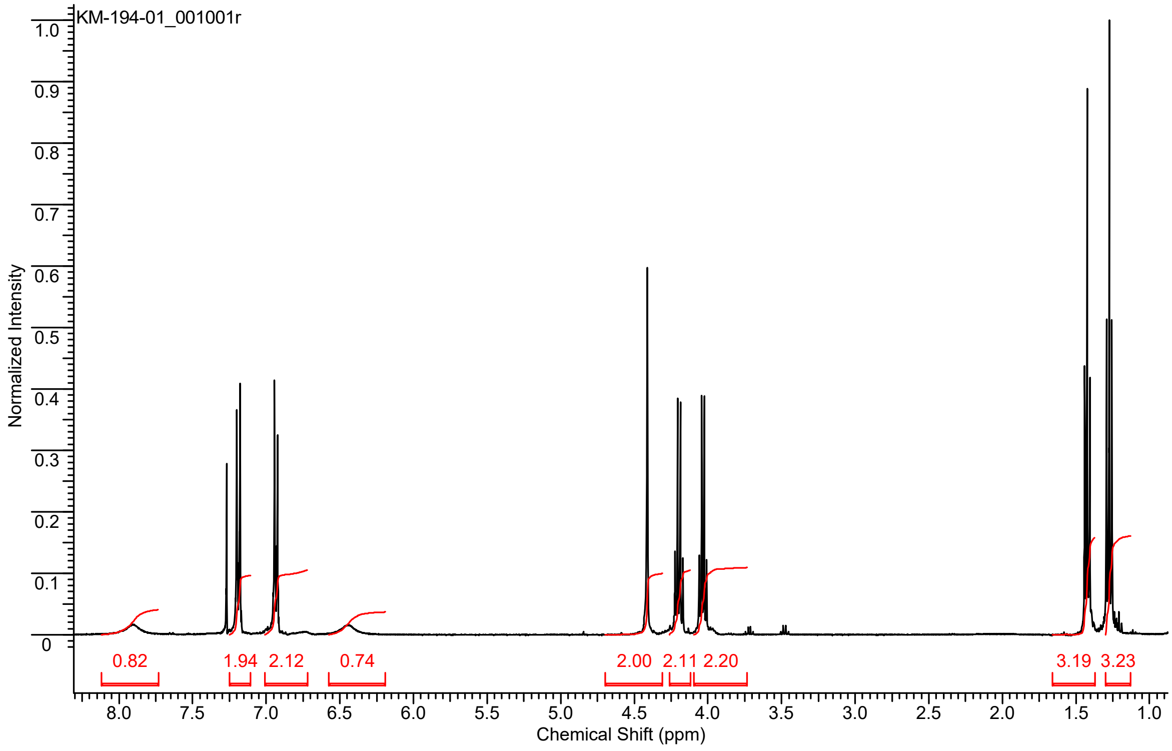

Ethyl 2-(3-(p-tolyl)thioureido)acetate (1e)

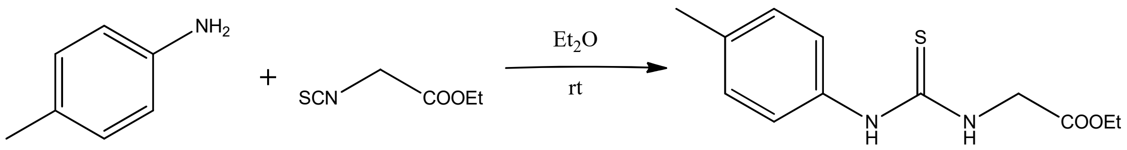

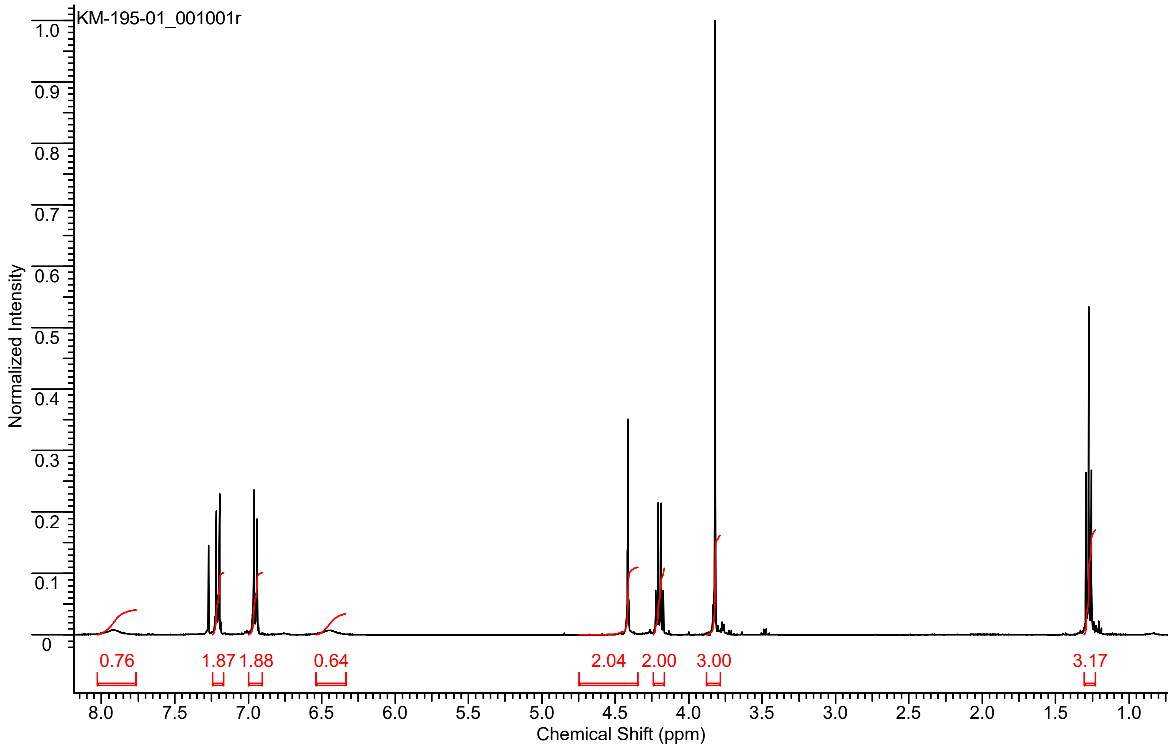

Ethyl 2-(3-(4-chlorophenyl)thioureido)acetate (1f)

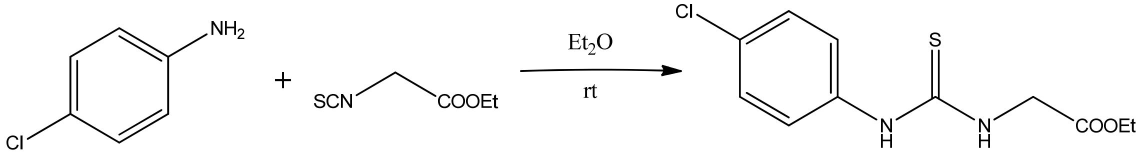

Chemical Formula: $C_{11}H_{13}ClN_2O_2S$
Molecular Weight: 272,7511

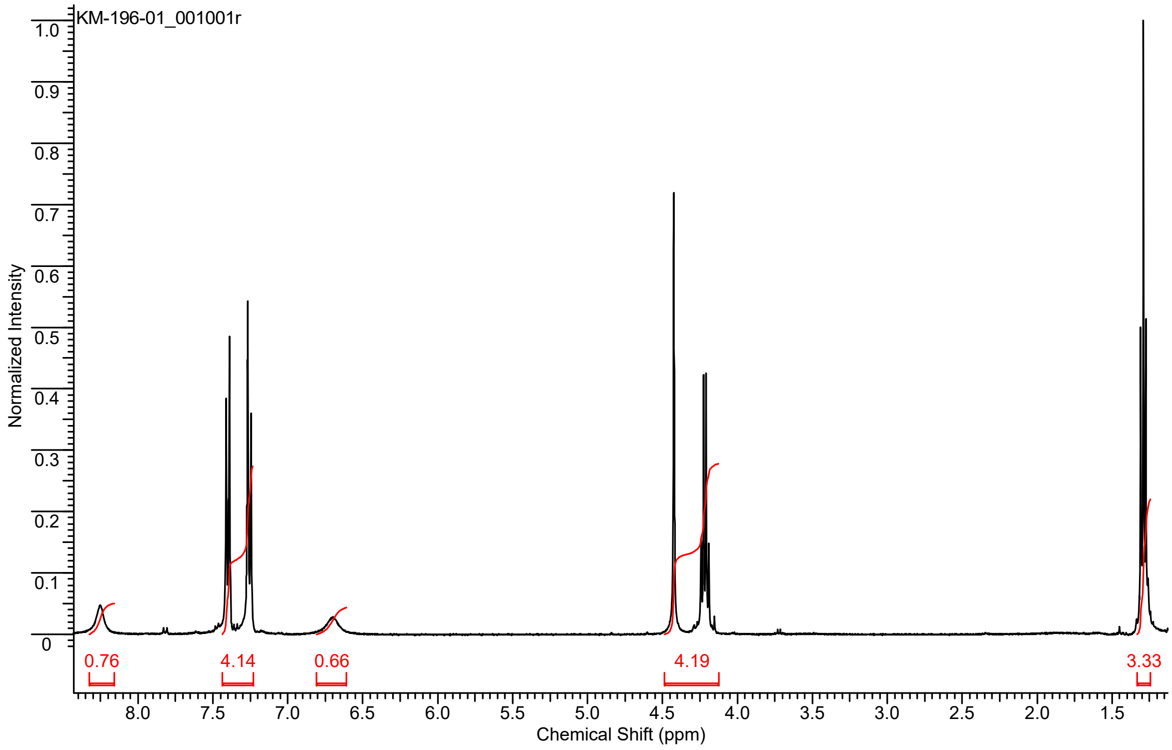

Ethyl 2-(3-(4-fluorophenyl)thioureido)acetate (1g)

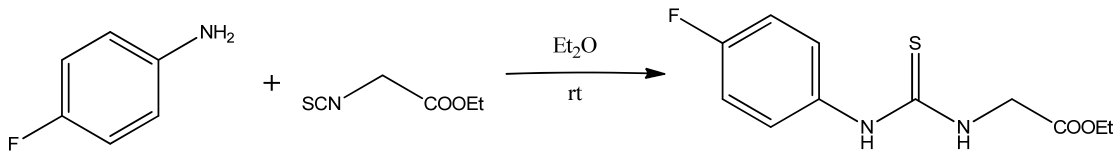

Chemical Formula: $C_{11}H_{13}FN_2O_2S$
Molecular Weight: 256,2965

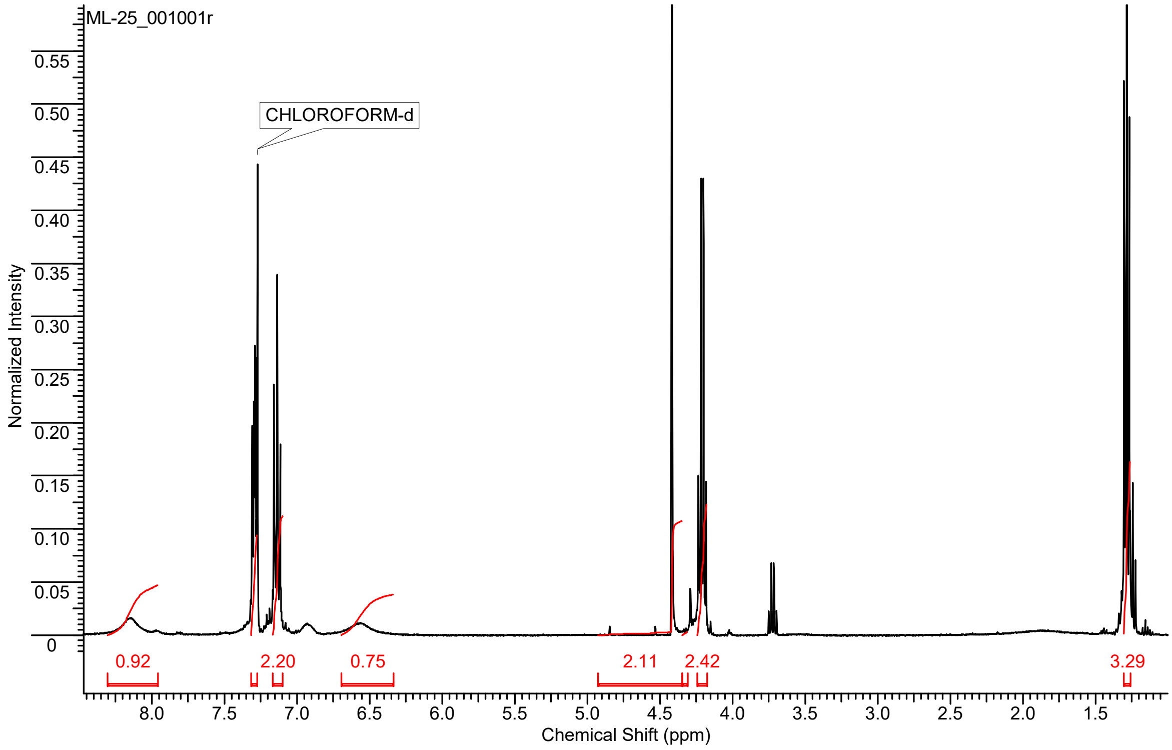

Ethyl 2-(3-(3-chlorobenzyl)thioureido)acetate (1h)

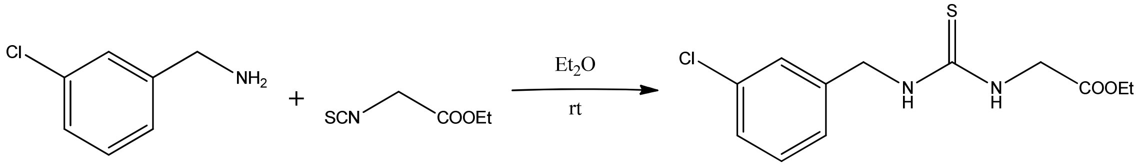

Chemical Formula: $C_{12}H_{15}ClN_2O_2S$
Molecular Weight: 286,7777

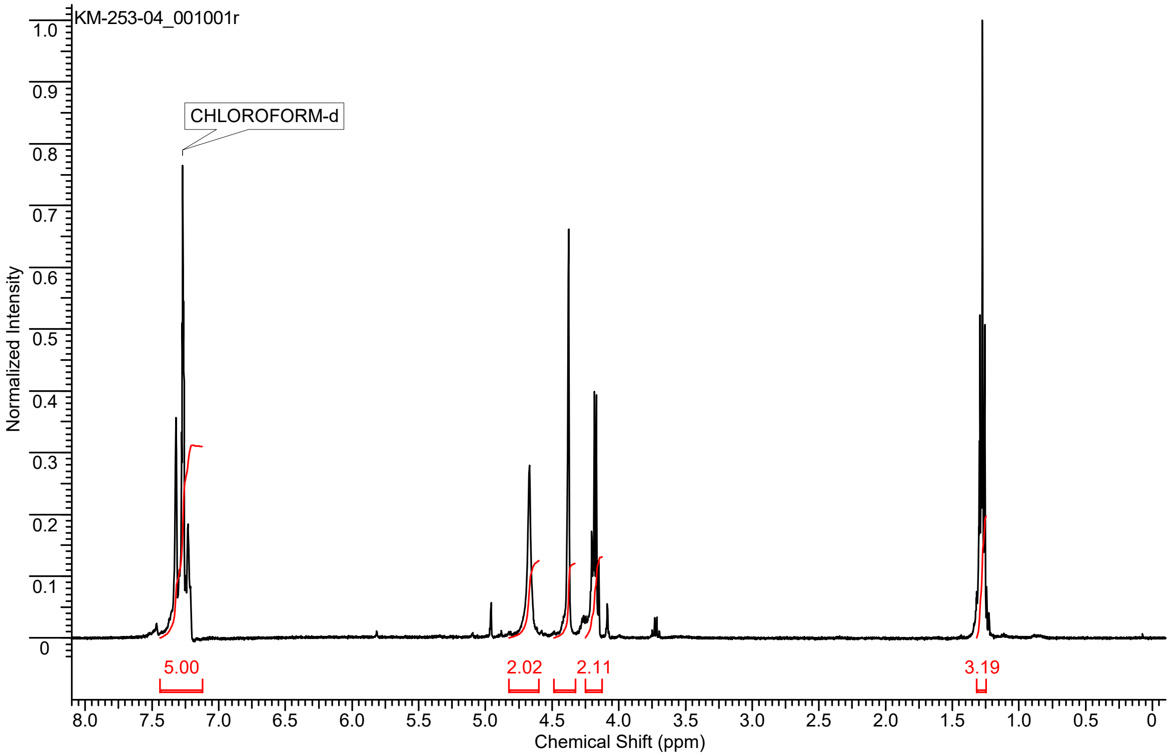

Ethyl 2-(3-(3-chloro-4-fluorophenyl)thioureido)acetate (1i)

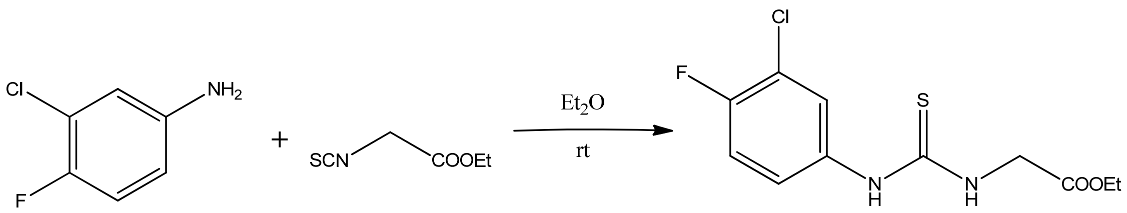

Chemical Formula: $C_{11}H_{12}ClFN_2O_2S$
Molecular Weight: 290,7416

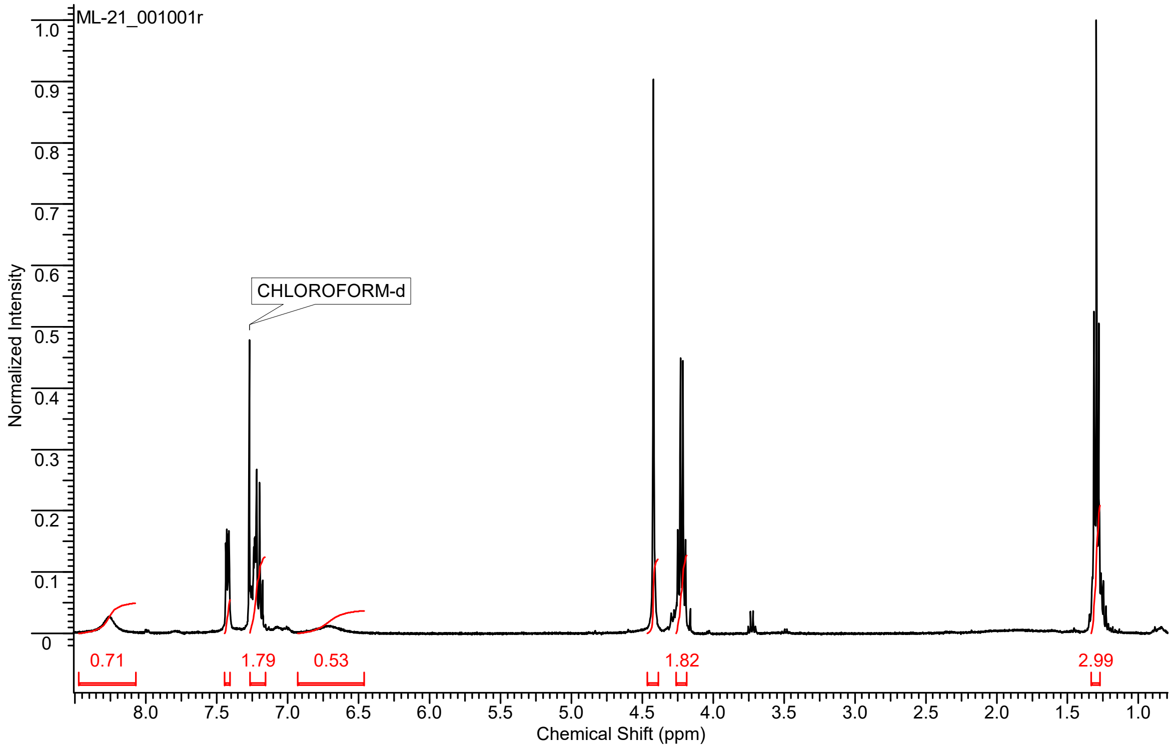

Ethyl 2-(3-cyclopropylthioureido)acetate (1j)

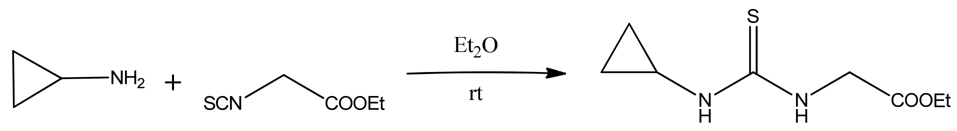

Chemical Formula: $C_8H_{14}N_2O_2S$
Molecular Weight: 202,2740

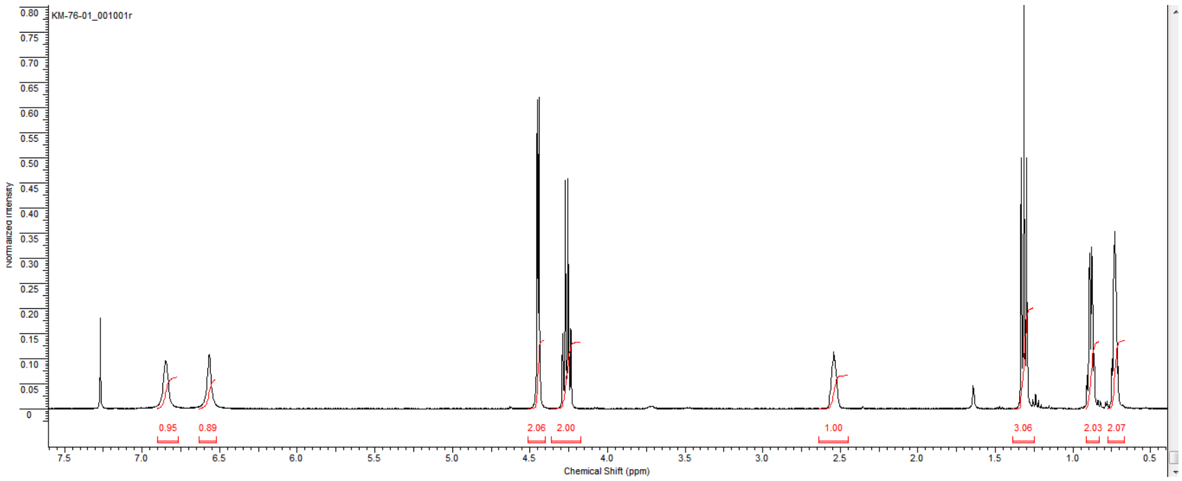

3-(3-Morpholinopropyl)-2-thioxoimidazolidin-4-one (2b)

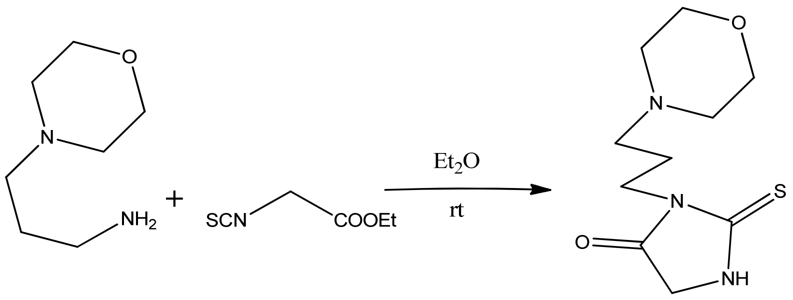

Chemical Formula: $C_{10}H_{17}N_3O_2S$
Molecular Weight: 243,3259

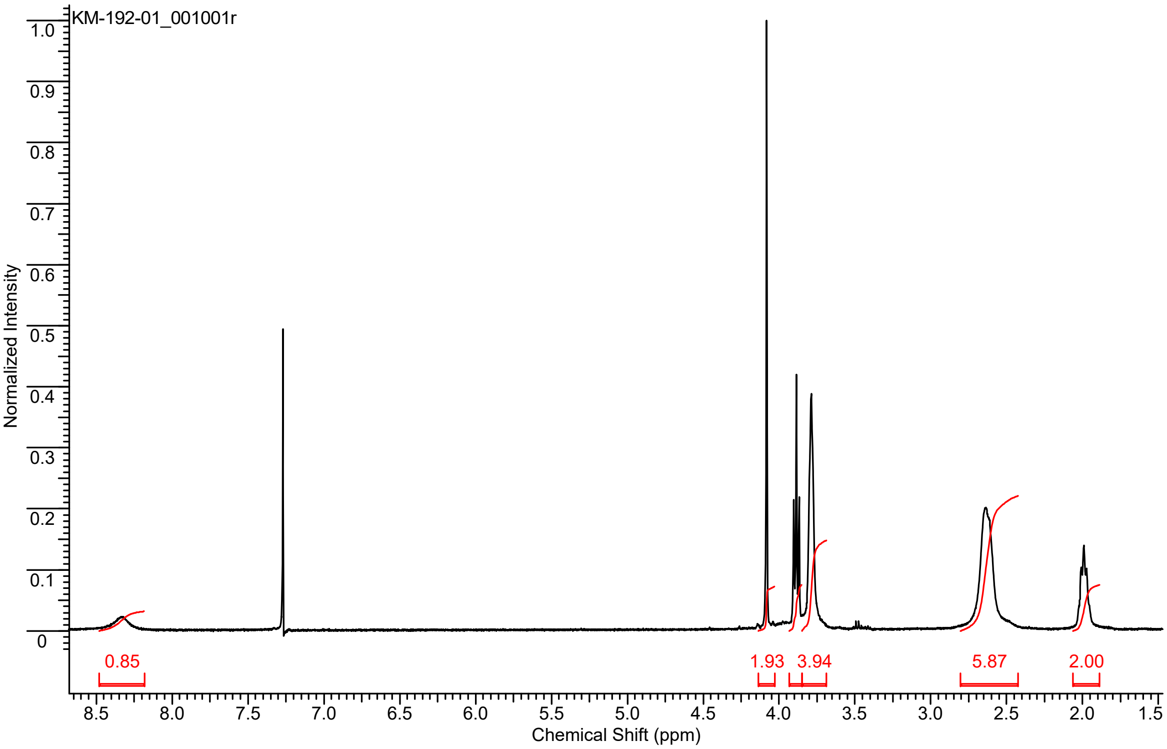

(Z)-3-(1-Benzyl-5-oxo-2-thioxoimidazolidin-4-ylidene)indolin-2-one (3a)

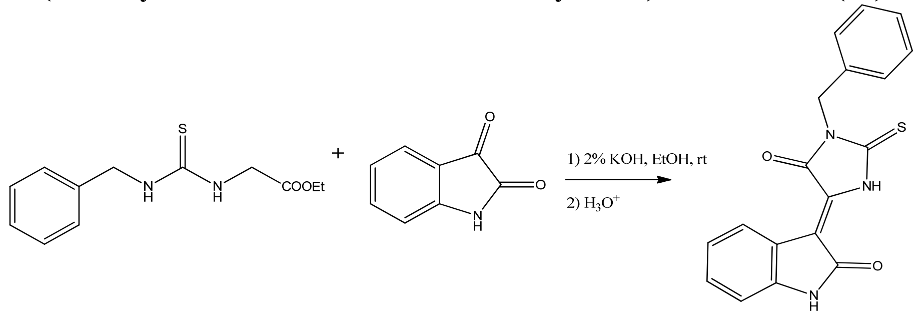

Chemical Formula: $C_{18}H_{13}N_3O_2S$
Molecular Weight: 335,3797

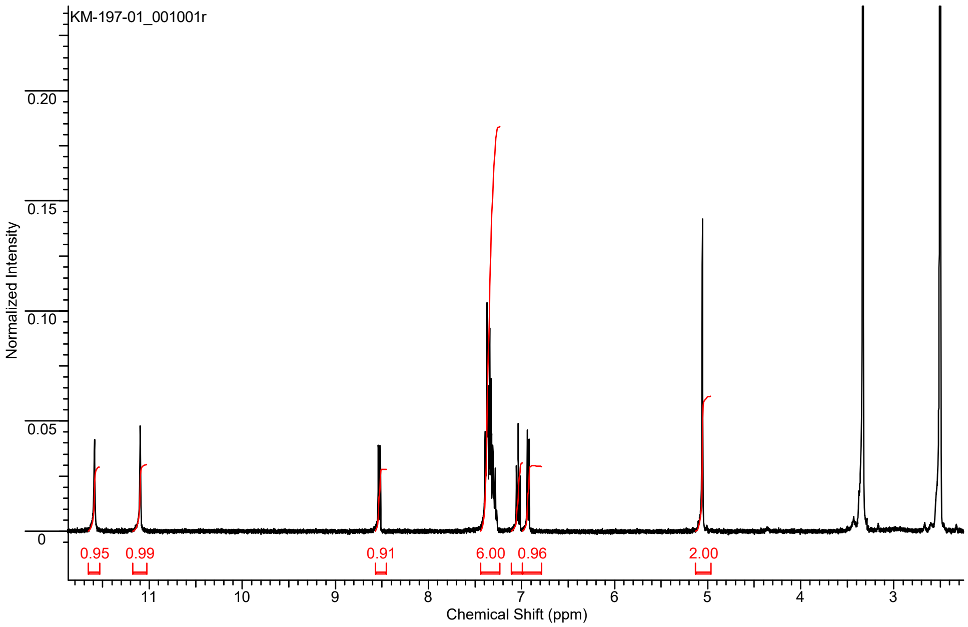

(Z)-3-(1-Benzyl-5-oxo-2-thioxoimidazolidin-4-ylidene)-5-chloroindolin-2-one (3b)

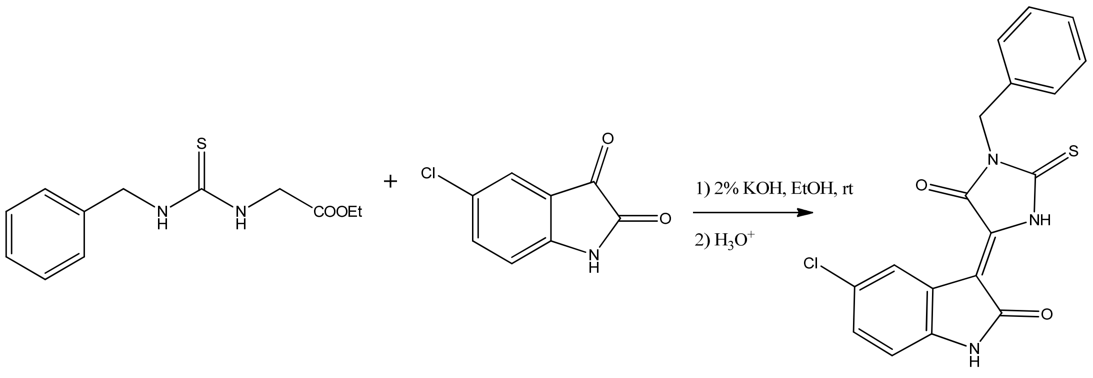

Chemical Formula: $C_{18}H_{12}ClN_3O_2S$
Molecular Weight: 369,8248

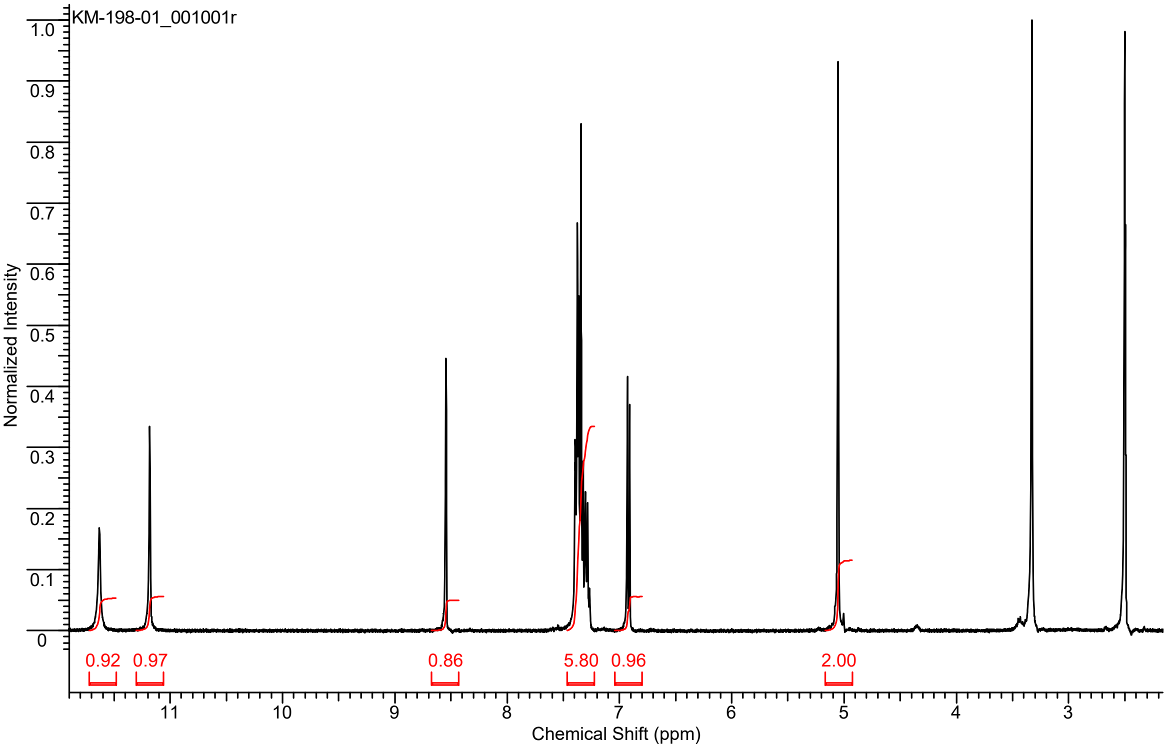

(Z)-3-(1-Allyl-5-oxo-2-thioxoimidazolidin-4-ylidene)indolin-2-one (3c)

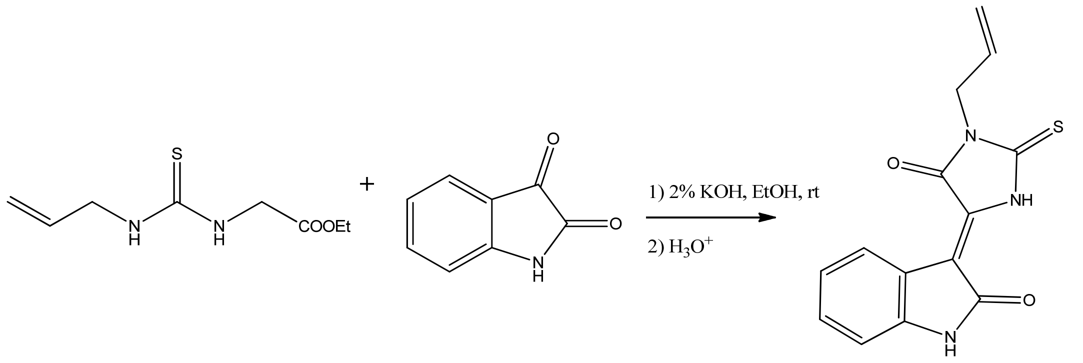

Chemical Formula: $C_{14}H_{11}N_3O_2S$
Molecular Weight: 285,3210

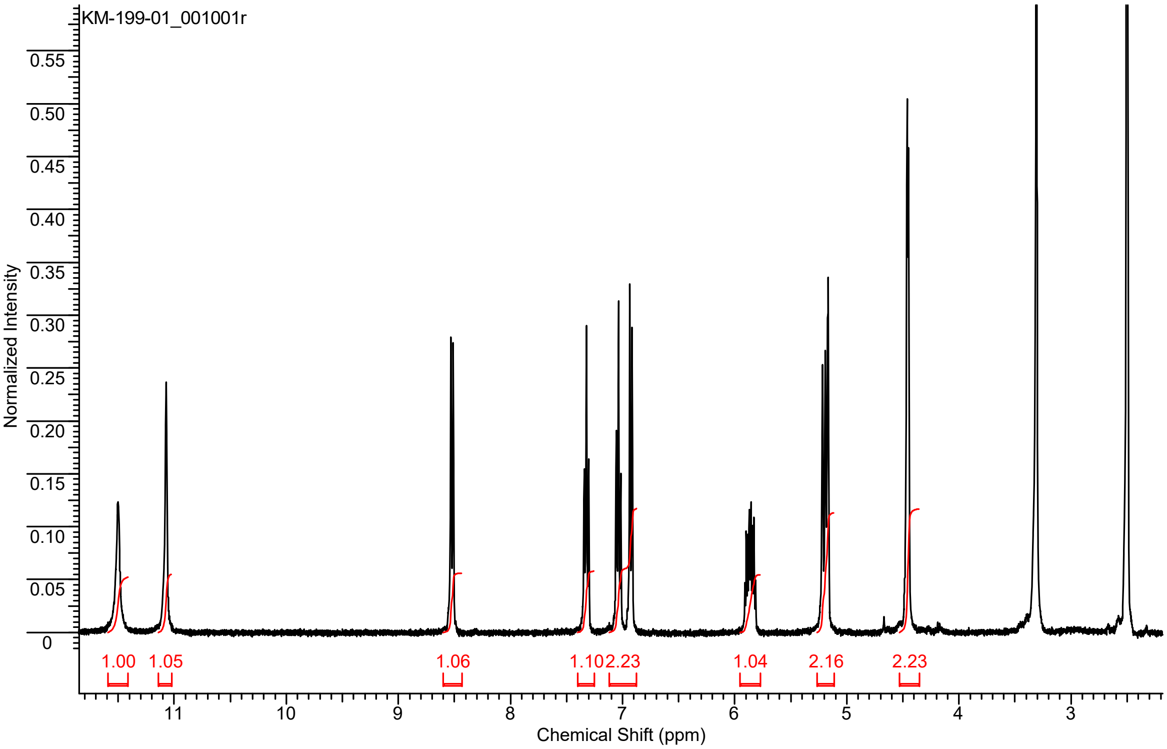

(Z)-3-(1-Allyl-5-oxo-2-thioxoimidazolidin-4-ylidene)-5-chloroindolin-2-one (3d)

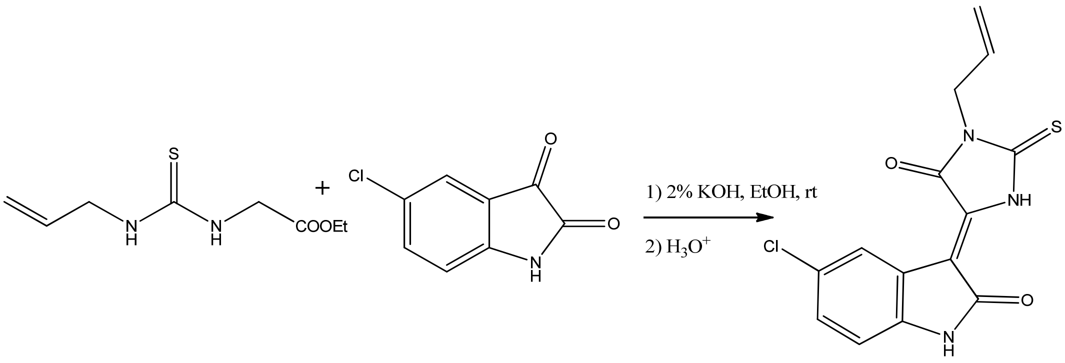

Chemical Formula: $C_{14}H_{10}ClN_3O_2S$
Molecular Weight: 319,7661

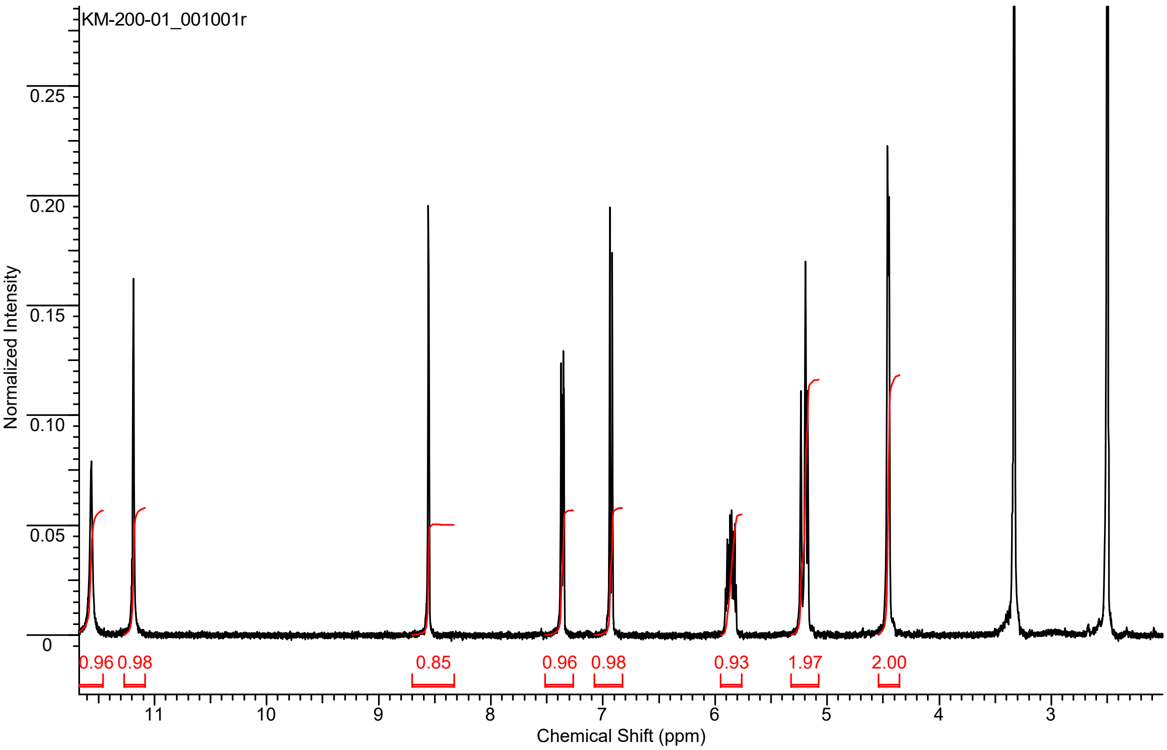

(Z)-3-(1-(4-Methoxyphenyl)-5-oxo-2-thioxoimidazolidin-4-ylidene)indolin-2-one (3e)

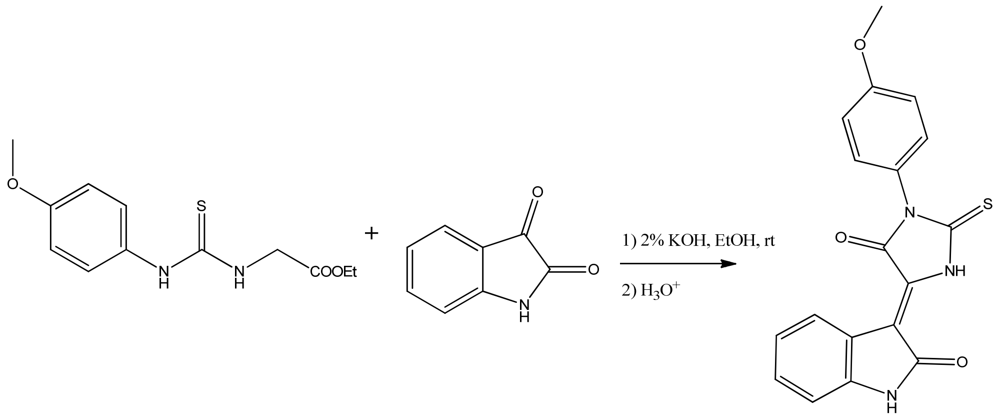

Chemical Formula: $C_{18}H_{13}N_3O_3S$
Molecular Weight: 351,3791

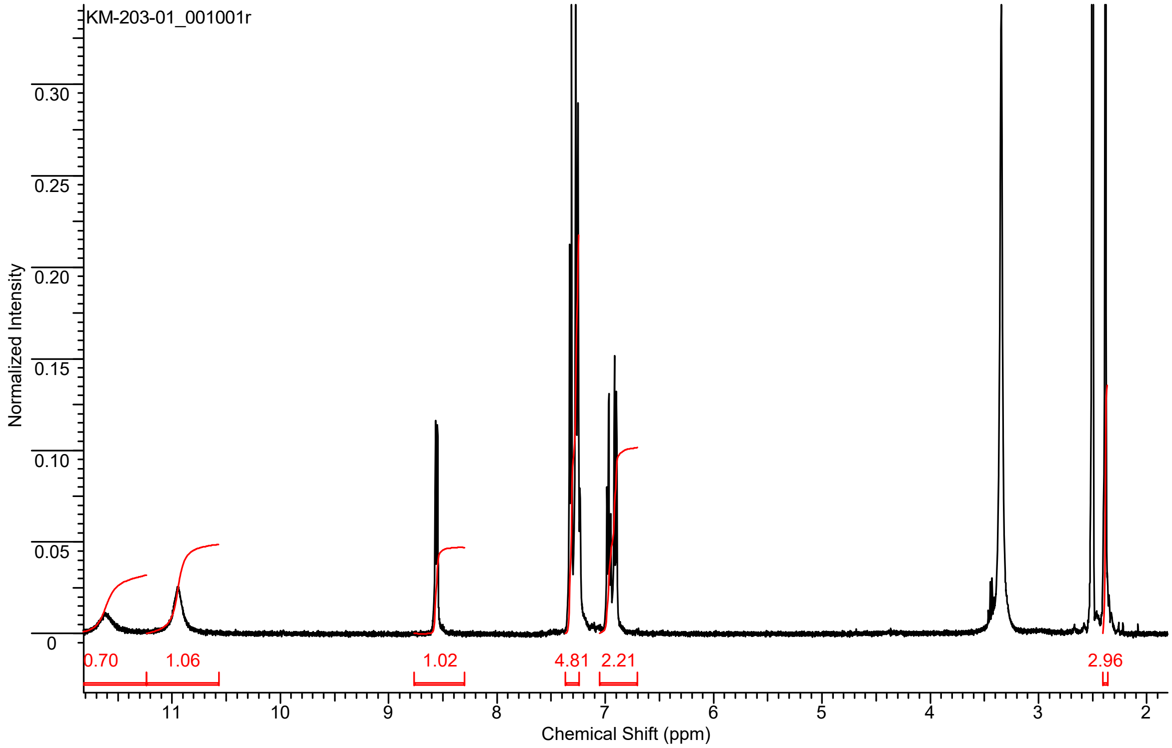

(Z)-5-Chloro-3-(1-(4-methoxyphenyl)-5-oxo-2-thioxoimidazolidin-4-ylidene)indolin-2-one (3f)

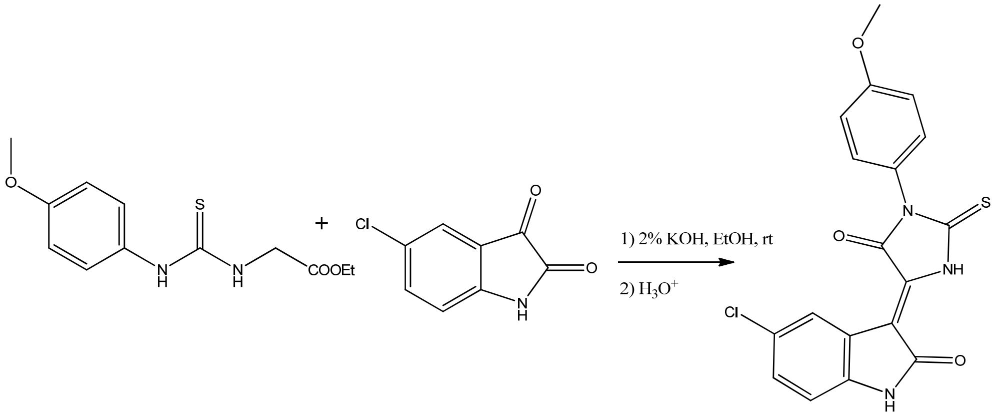

Chemical Formula: $C_{18}H_{12}ClN_3O_3S$
Molecular Weight: 385,8242

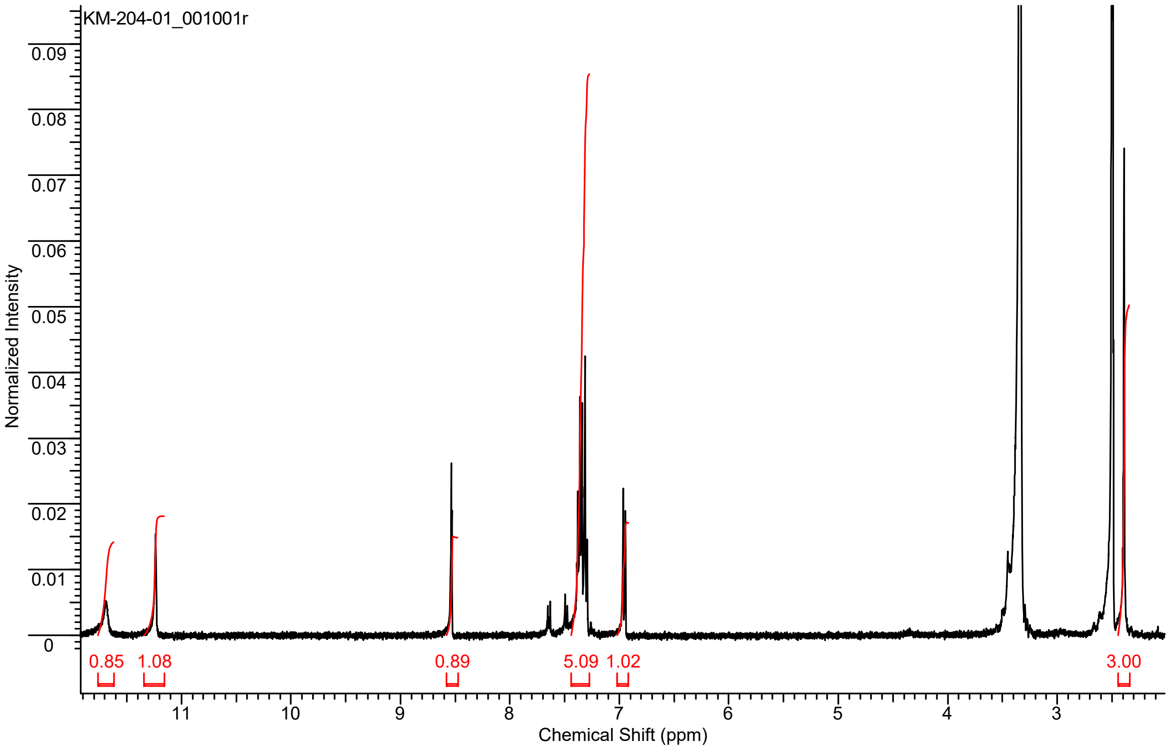

(Z)-3-(1-(4-Ethoxyphenyl)-5-oxo-2-thioxoimidazolidin-4-ylidene)indolin-2-one (3g)

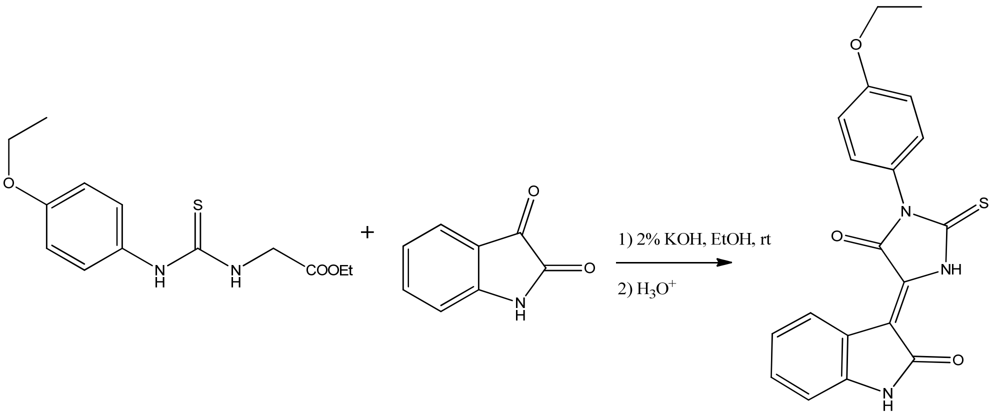

Chemical Formula: $C_{19}H_{15}N_3O_3S$
Molecular Weight: 365,4057

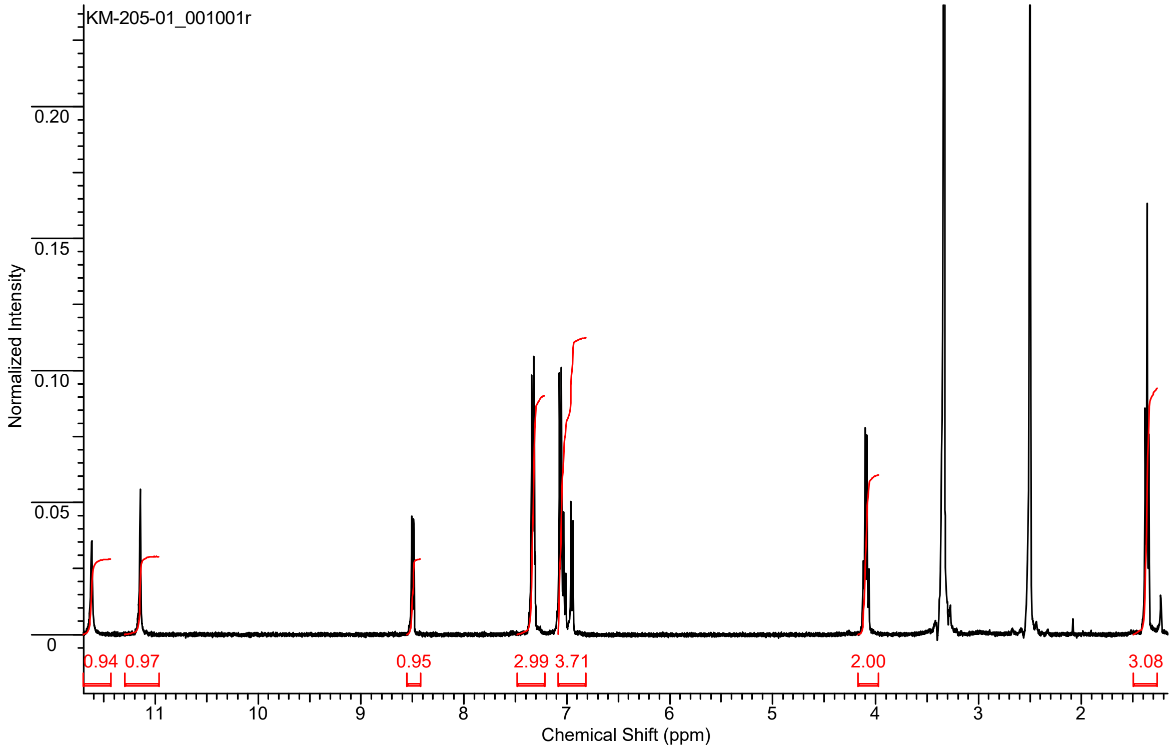

(Z)-5-Chloro-3-(1-(4-ethoxyphenyl)-5-oxo-2-thioxoimidazolidin-4-ylidene)indolin-2-one (3h)

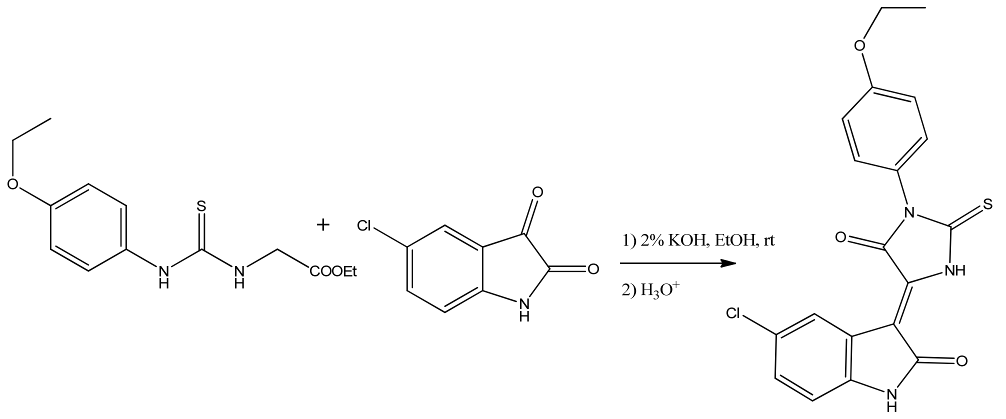

Chemical Formula: $C_{19}H_{14}ClN_3O_3S$
Molecular Weight: 399,8508

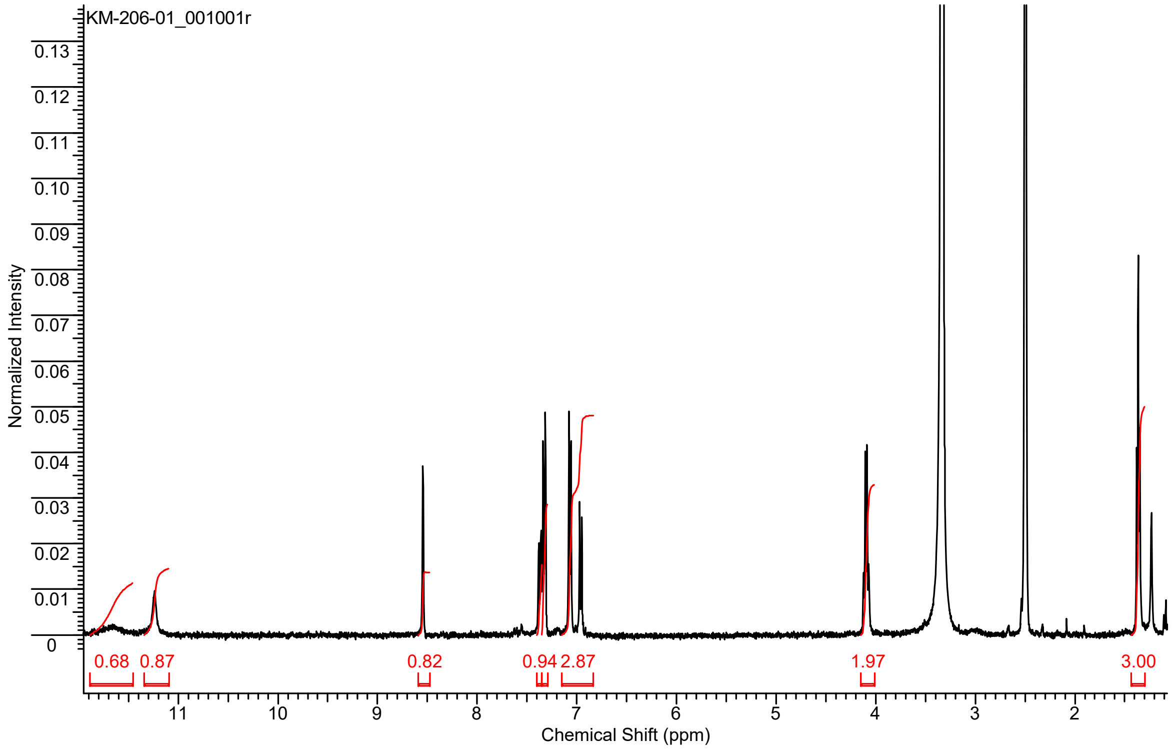

(Z)-3-(5-oxo-2-thioxo-1-(p-tolyl)imidazolidin-4-ylidene)indolin-2-one (3i)

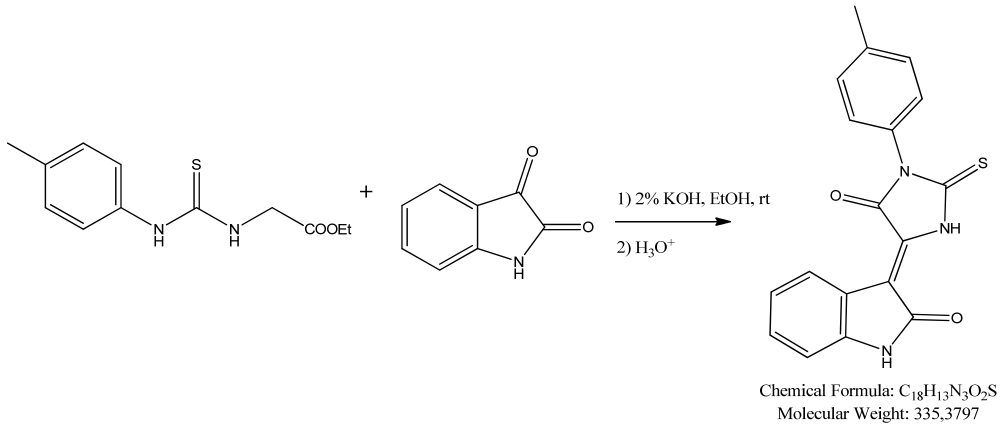

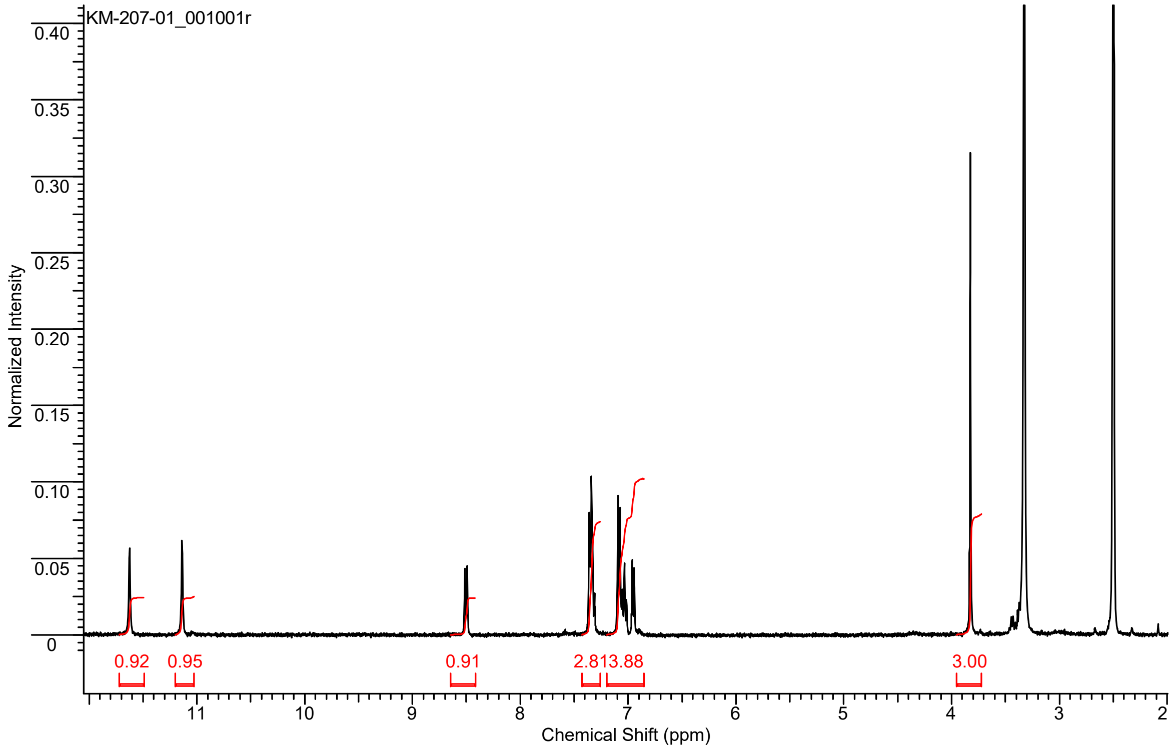

(Z)-5-Chloro-3-(5-oxo-2-thioxo-1-(p-tolyl)imidazolidin-4-ylidene)indolin-2-one (3j)

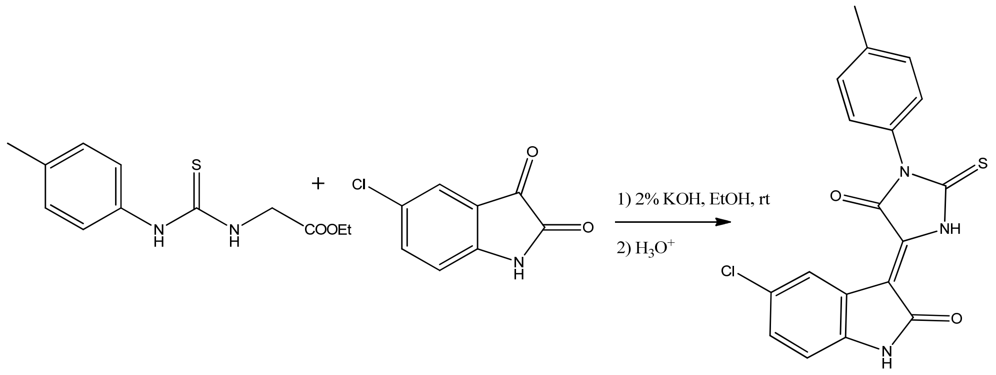

Chemical Formula: $C_{18}H_{12}ClN_3O_2S$
Molecular Weight: 369,8248

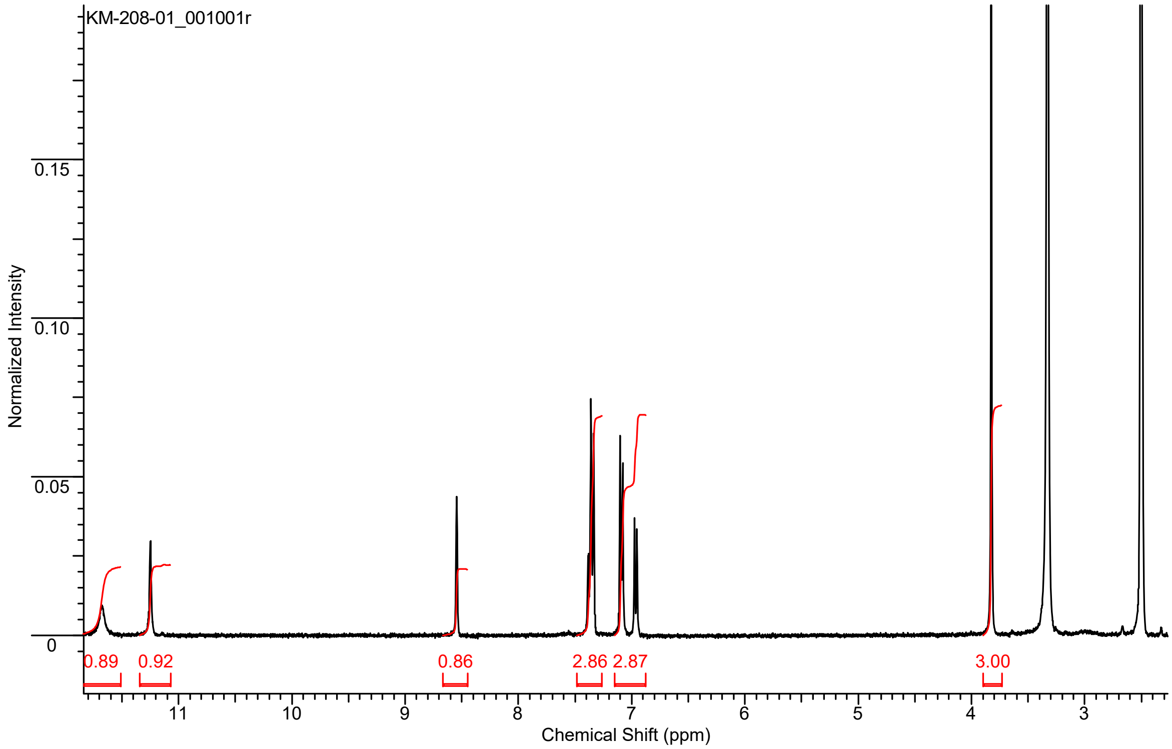

(Z)-3-(1-(4-Chlorophenyl)-5-oxo-2-thioxoimidazolidin-4-ylidene)indolin-2-one (3k)

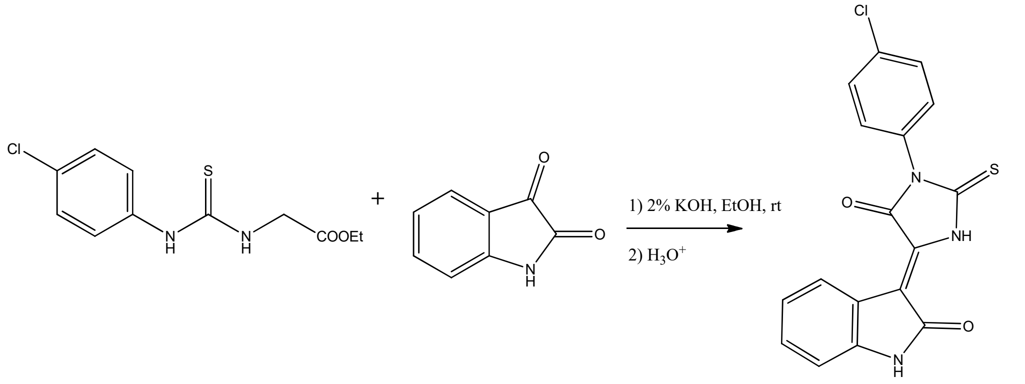

Chemical Formula: $C_{17}H_{10}ClN_3O_2S$
Molecular Weight: 355,7982

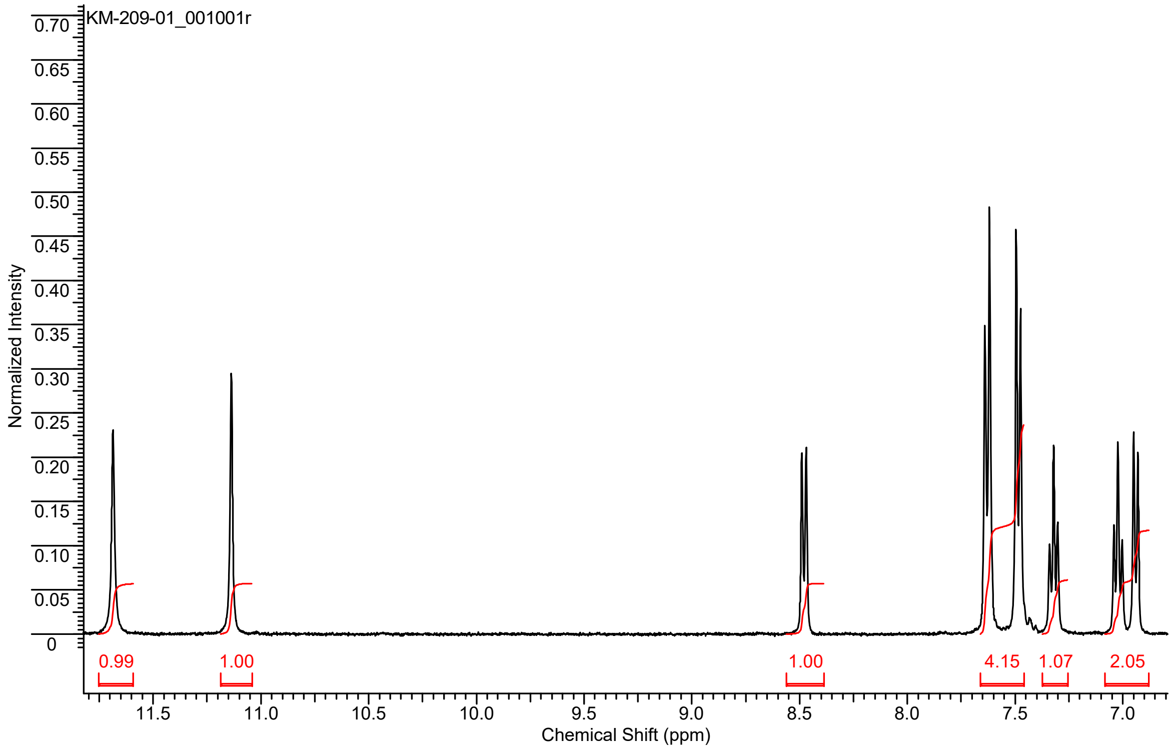

(Z)-5-Chloro-3-(1-(4-chlorophenyl)-5-oxo-2-thioxoimidazolidin-4-ylidene)indolin-2-one (3l)

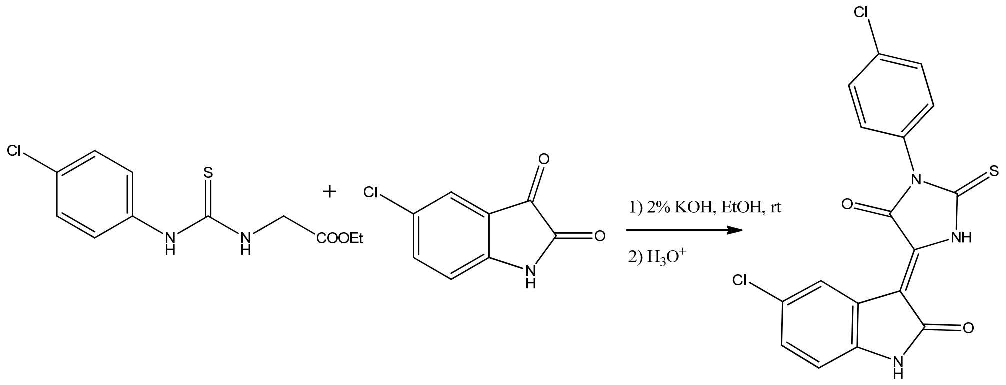

Chemical Formula: $C_{17}H_9Cl_2N_3O_2S$
Molecular Weight: 390,2433

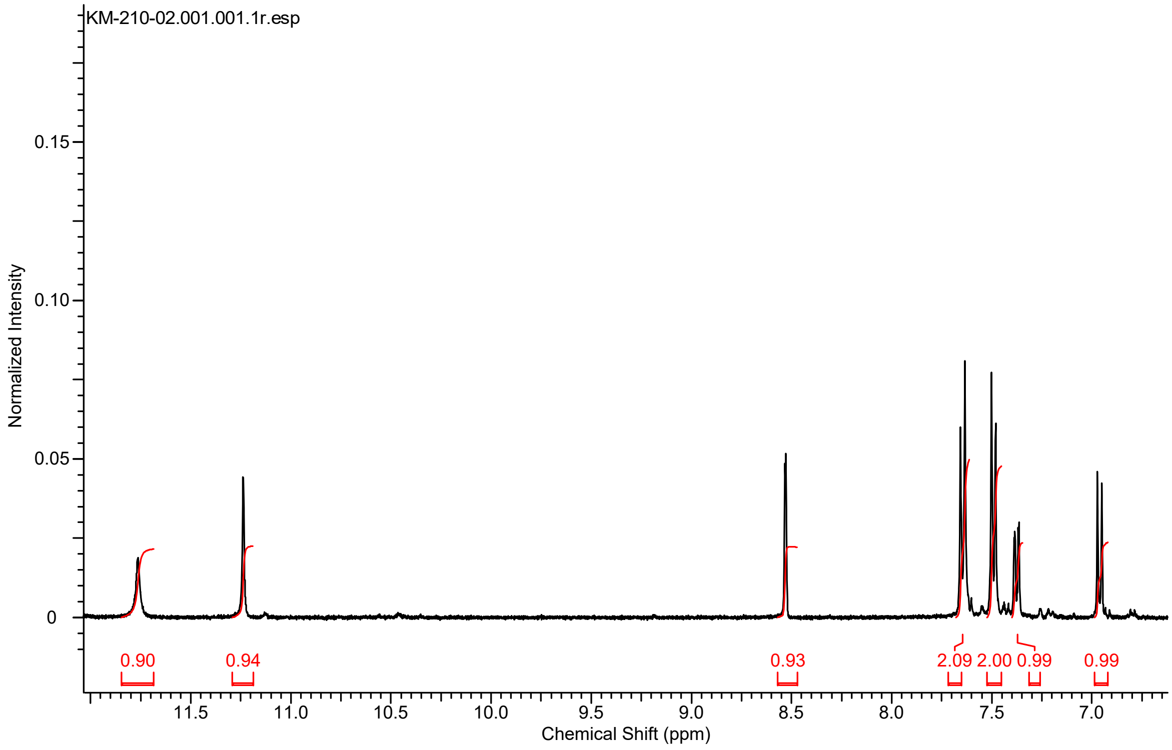

(Z)-3-(1-(4-Fluorophenyl)-5-oxo-2-thioxoimidazolidin-4-ylidene)indolin-2-one (3m)

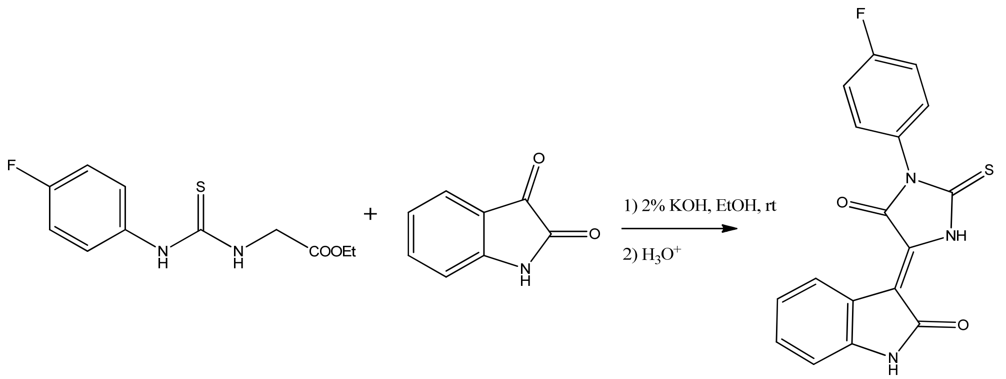

Chemical Formula: $C_{17}H_{10}FN_3O_2S$

Molecular Weight: 339,3436

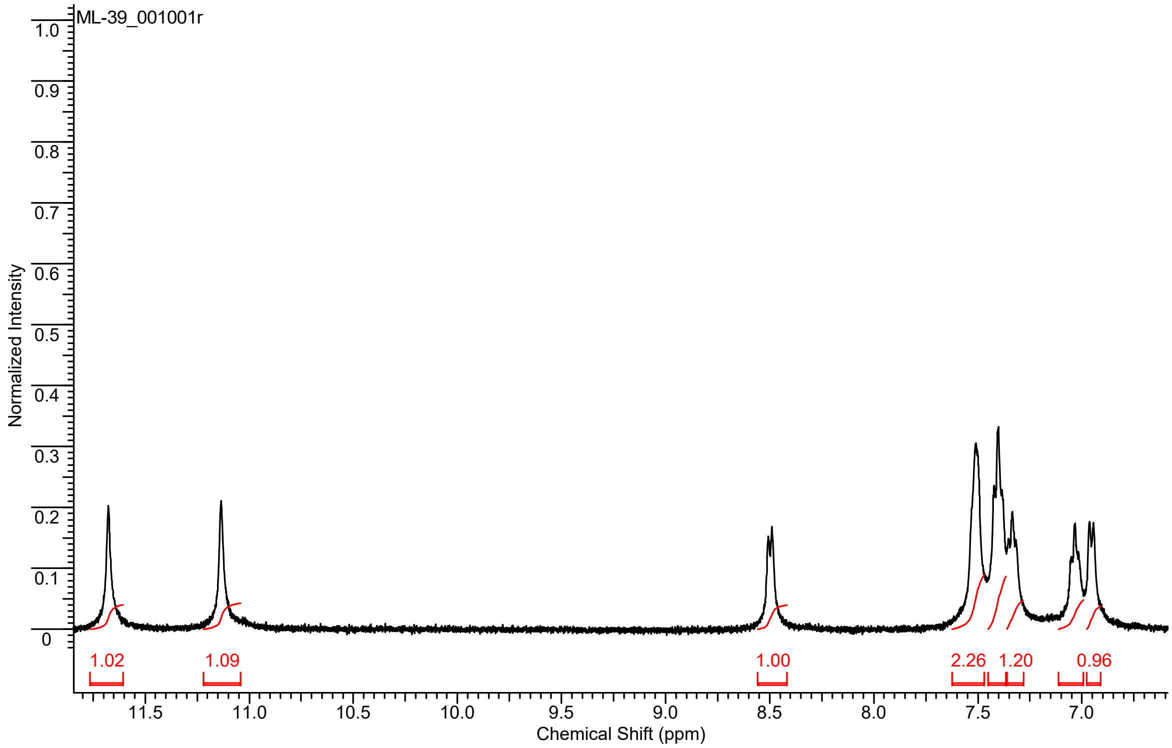

(Z)-5-Chloro-3-(1-(4-fluorophenyl)-5-oxo-2-thioxoimidazolidin-4-ylidene)indolin-2-one (3n)

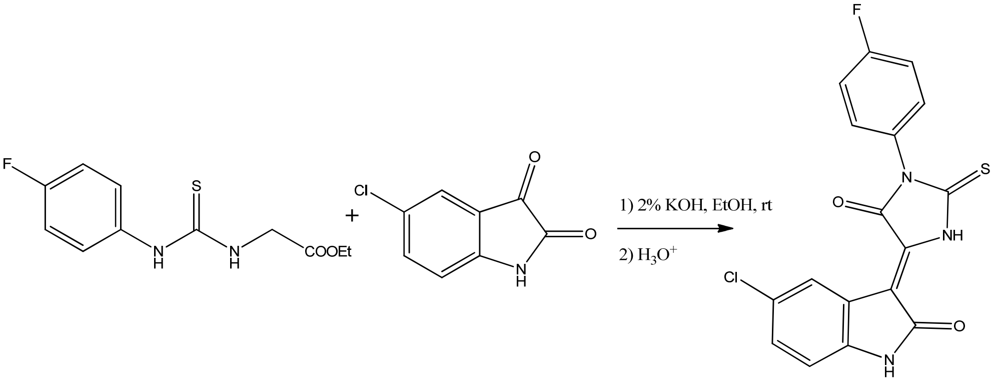

Chemical Formula: $C_{17}H_9ClFN_3O_2S$
Molecular Weight: 373,7887

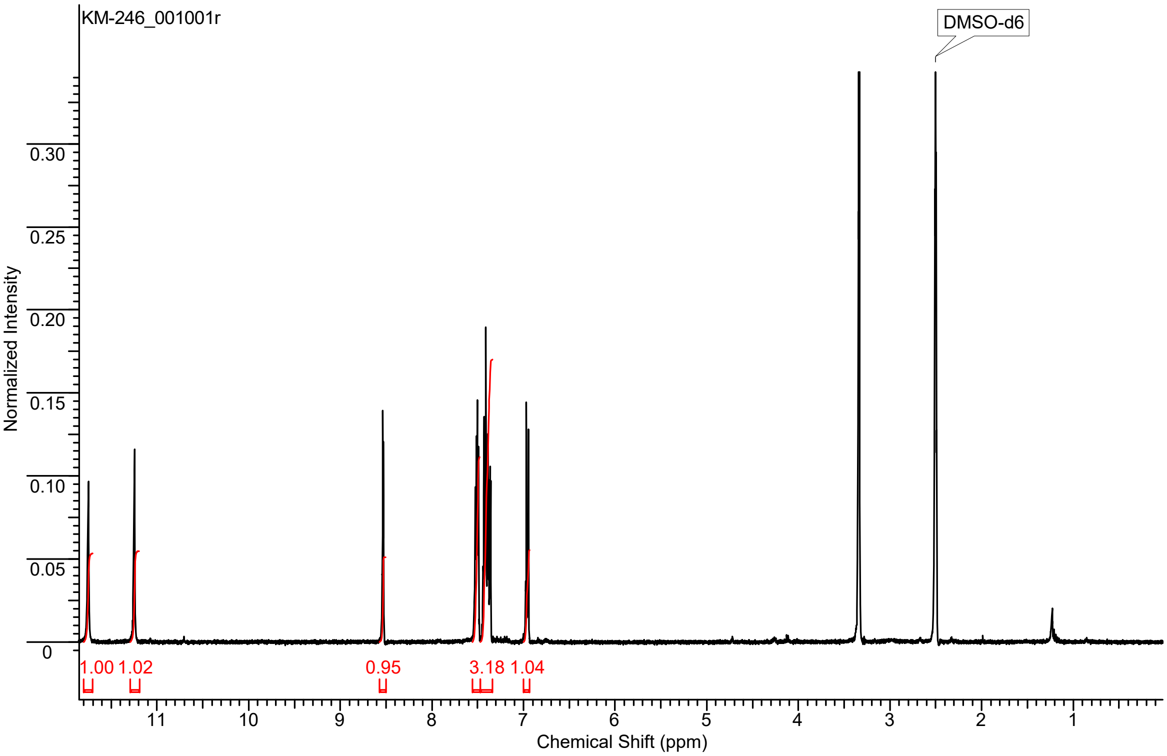

(Z)-3-(1-(3-Chlorobenzyl)-5-oxo-2-thioxoimidazolidin-4-ylidene)indolin-2-one (3o)

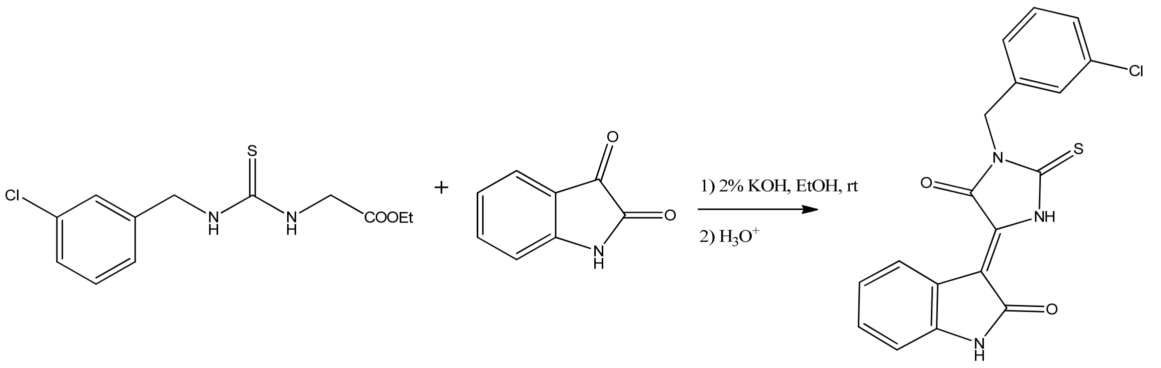

Chemical Formula: $C_{18}H_{12}ClN_3O_2S$

Molecular Weight: 369,82

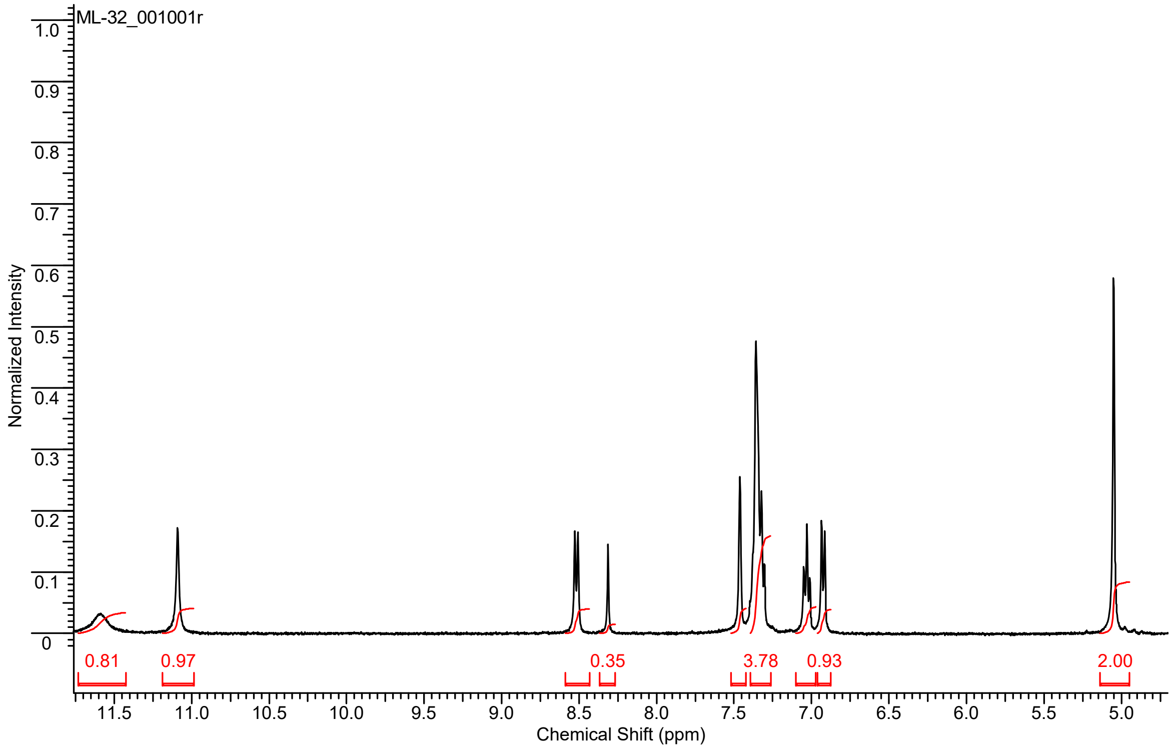

(Z)-5-Chloro-3-(1-(3-chlorobenzyl)-5-oxo-2-thioxoimidazolidin-4-ylidene)indolin-2-one (3p)

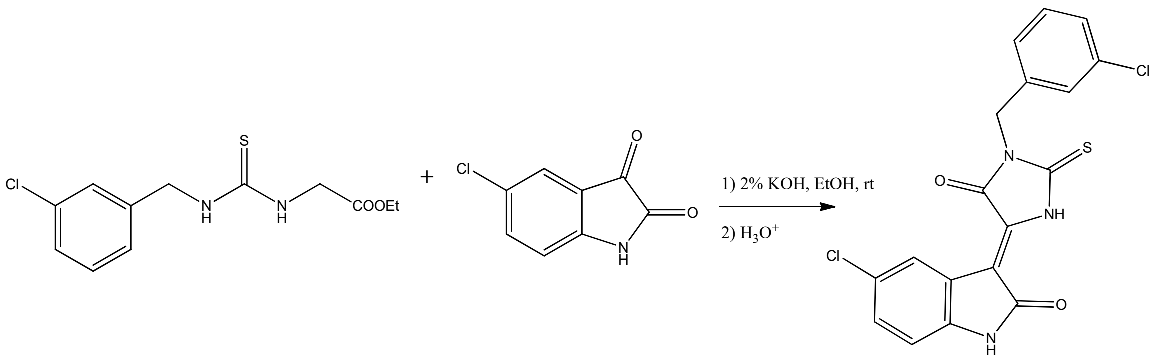

Chemical Formula: $C_{18}H_{11}Cl_2N_3O_2S$

Molecular Weight: 404,2698

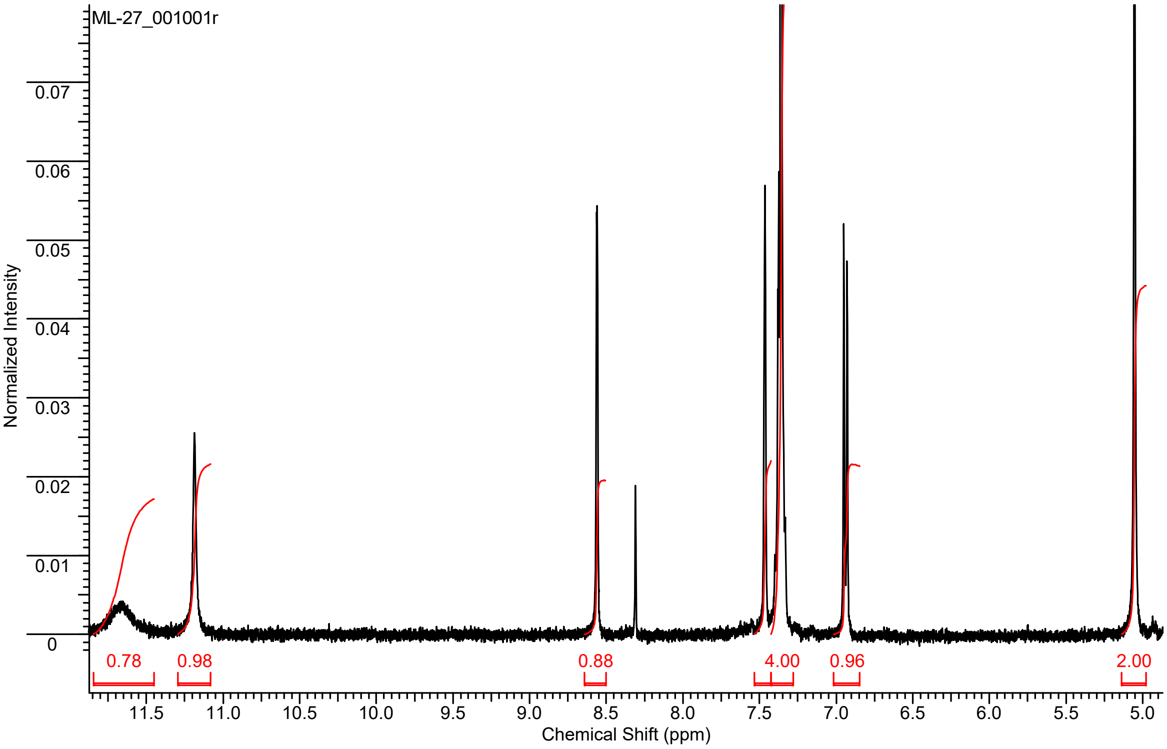

(Z)-3-(1-(3-Chloro-4-fluorophenyl)-5-oxo-2-thioxoimidazolidin-4-ylidene)indolin-2-one (3q)

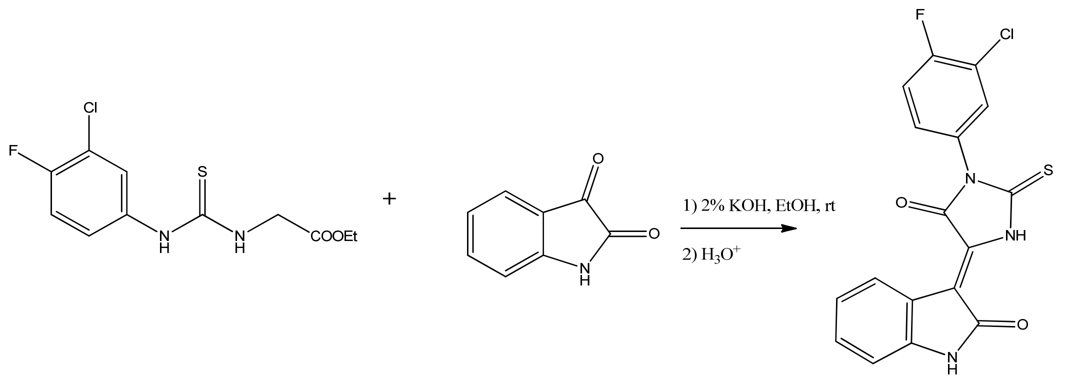

Chemical Formula: $C_{17}H_9ClFN_3O_2S$
Molecular Weight: 373,7887

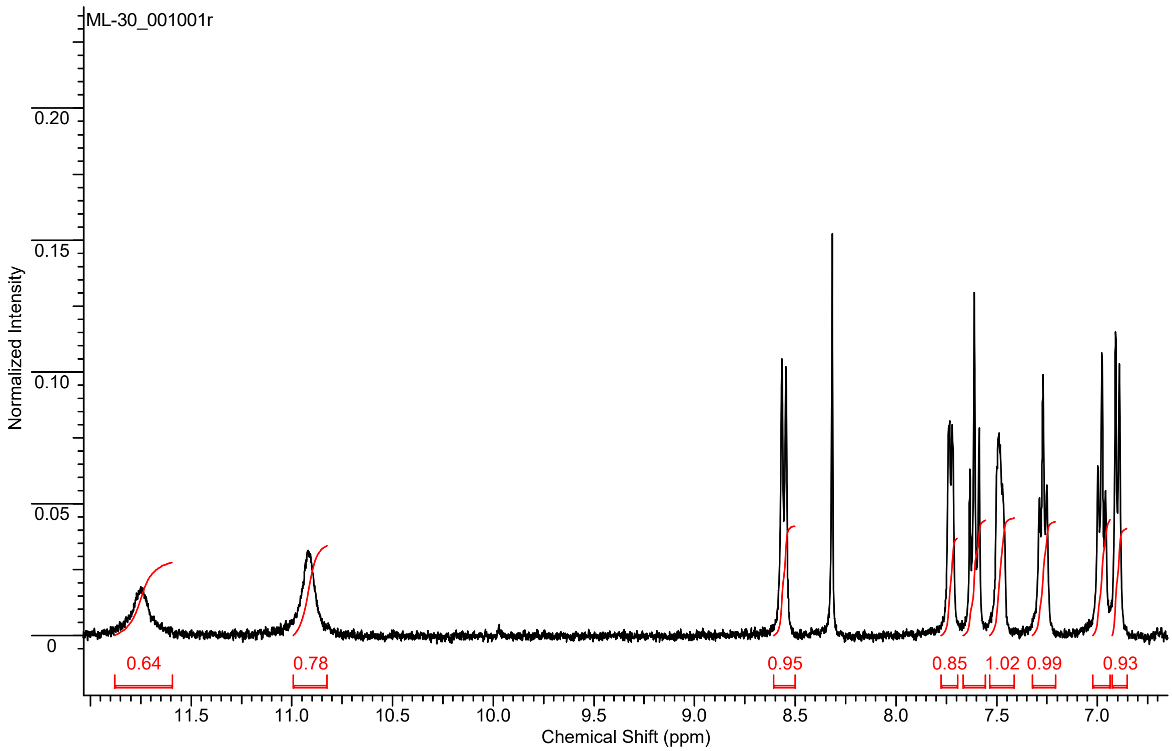

(Z)-5-Chloro-3-(1-(3-chloro-4-fluorophenyl)-5-oxo-2-thioxoimidazolidin-4-ylidene)indolin-2-one (3r)

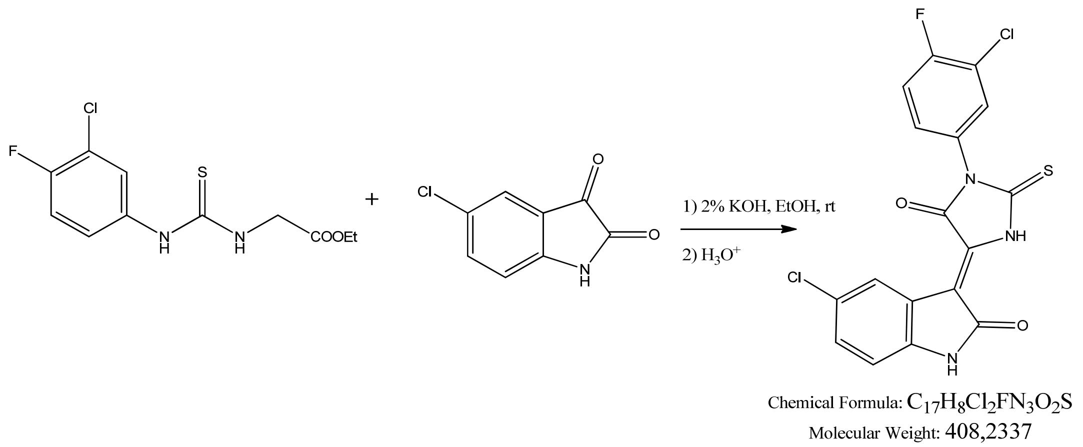

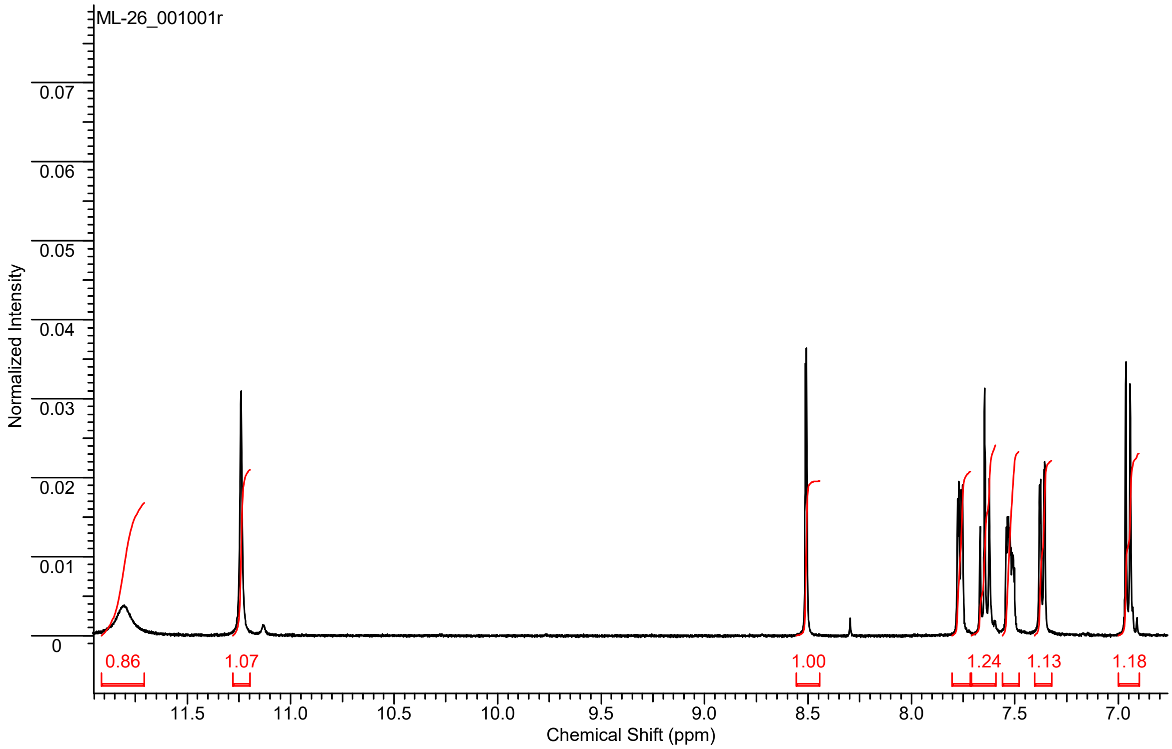

(Z)-3-(1-Cyclopropyl-5-oxo-2-thioxoimidazolidin-4-ylidene)indolin-2-one (3s)

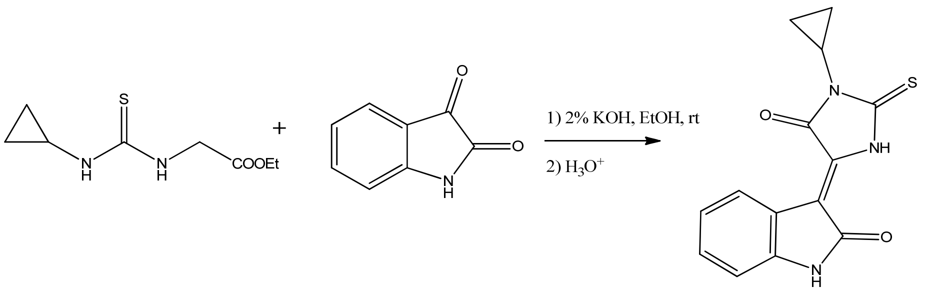

Chemical Formula: $C_{14}H_{11}N_3O_2S$
Molecular Weight: 285,3210

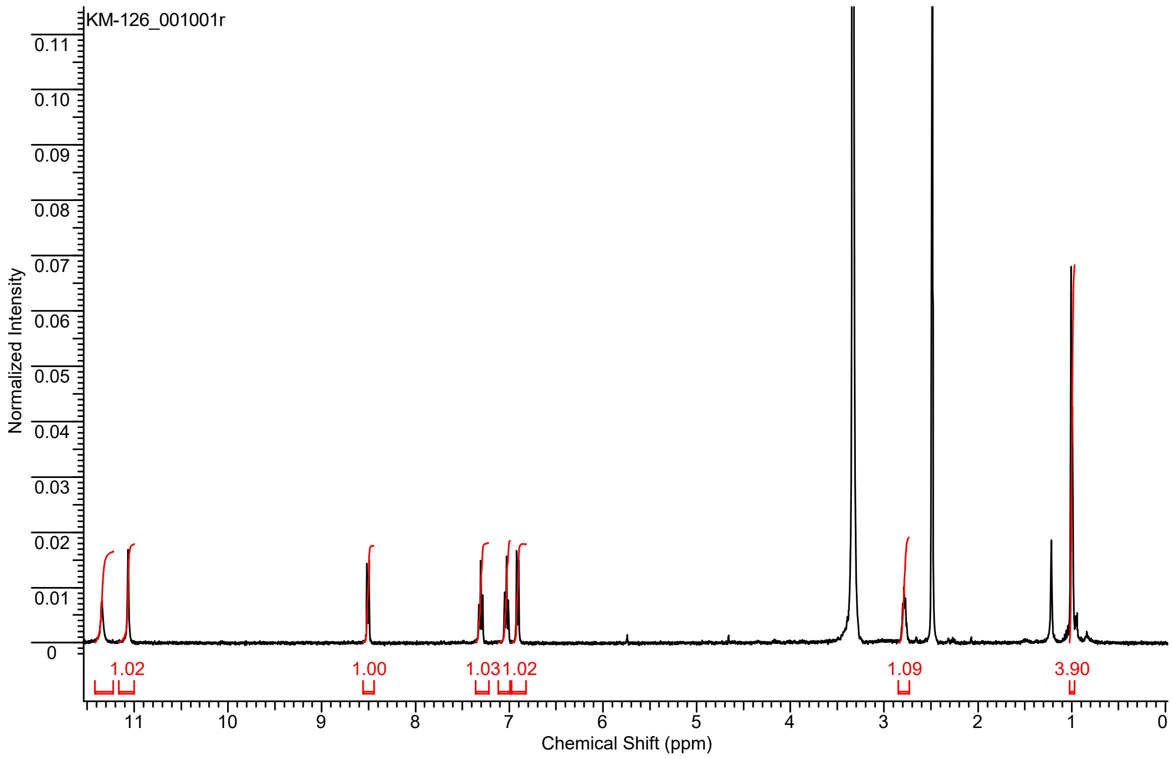

(Z)-5-Chloro-3-(1-cyclopropyl-5-oxo-2-thioxoimidazolidin-4-ylidene)indolin-2-one (3t)

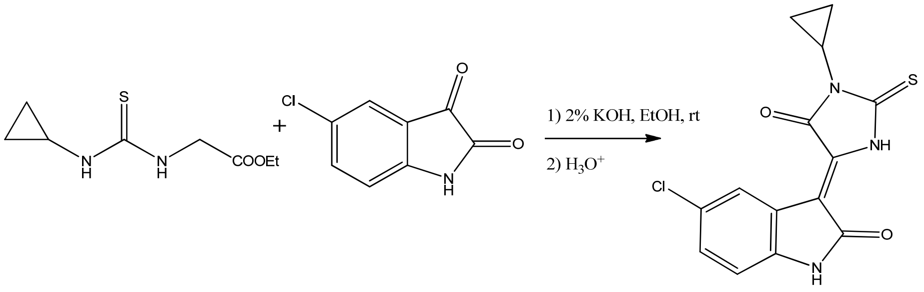

Chemical Formula: $C_{14}H_{10}ClN_3O_2S$
Molecular Weight: 319,7661

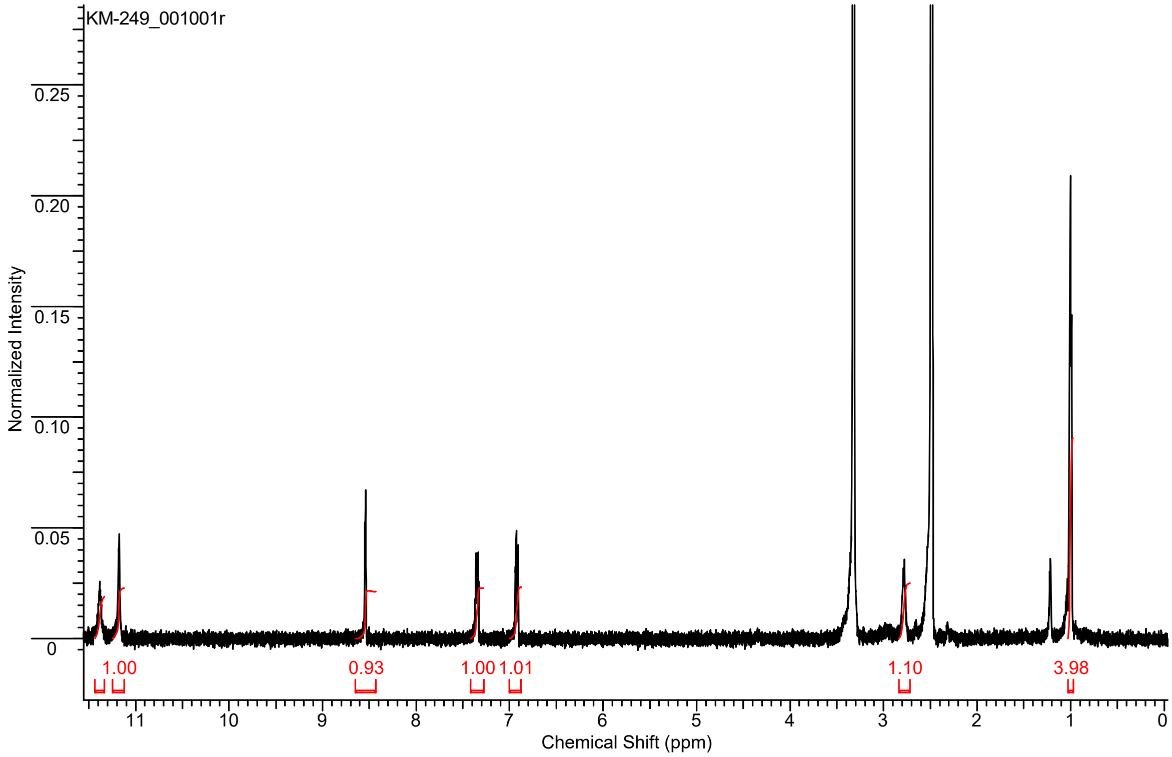

(Z)-3-(1-(3-Morpholinopropyl)-5-oxo-2-thioxoimidazolidin-4-ylidene)indolin-2-one hydrochloride (3u)

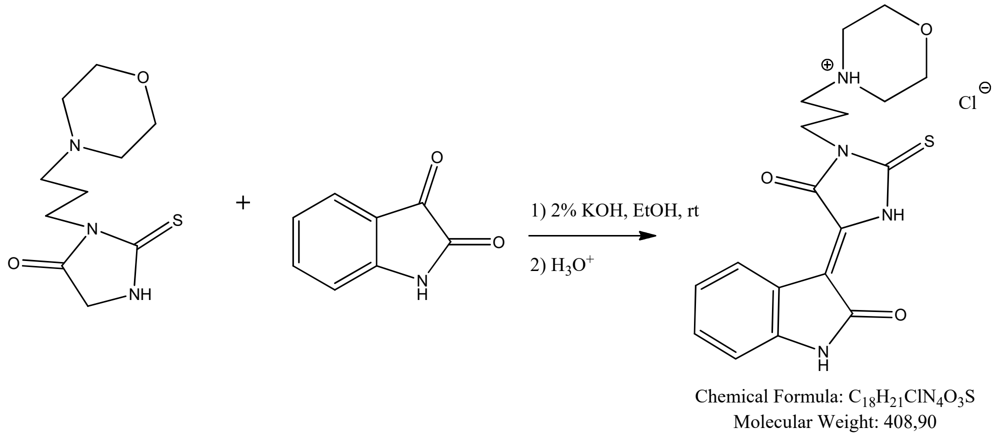

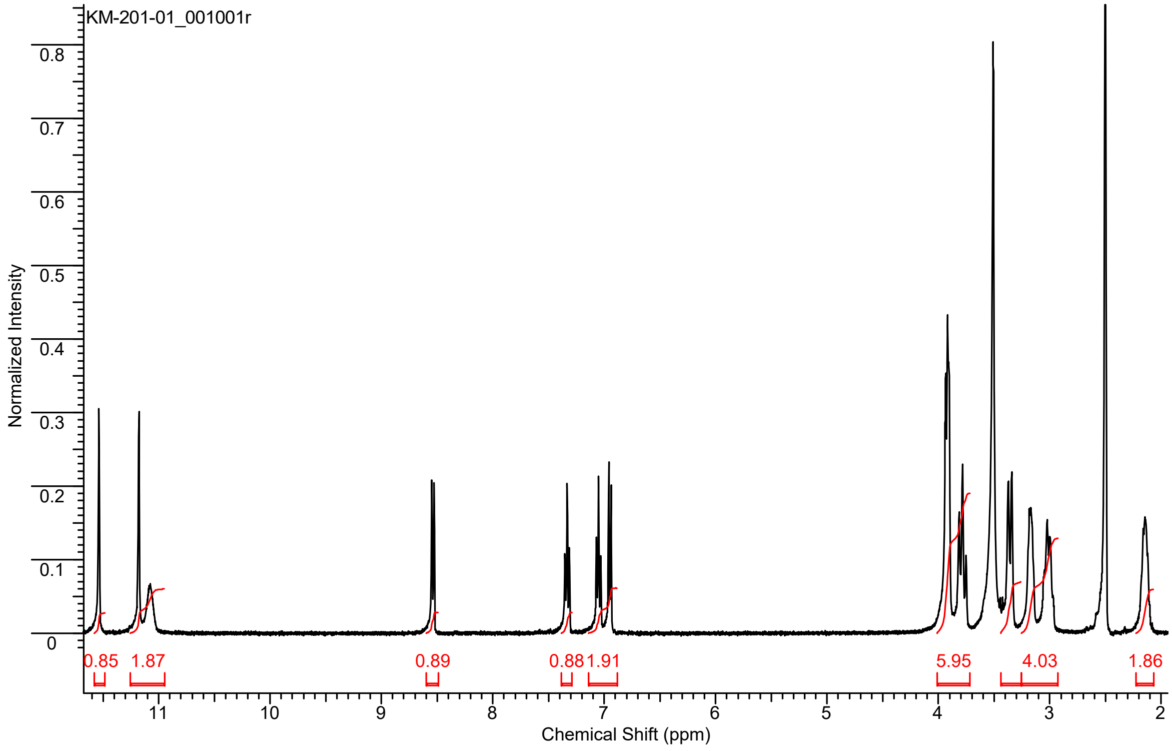

(Z)-5-Chloro -3-(1-(3-morpholinopropyl)-5-oxo-2-thioxoimidazolidin-4-ylidene)indolin-2-one hydrochloride (3v)

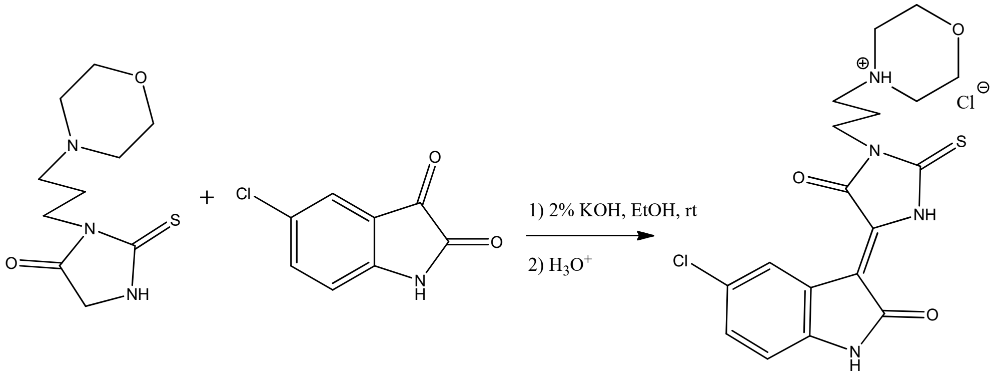

Chemical Formula: $C_{18}H_{20}Cl_2N_4O_3S$
Molecular Weight: 443,3474

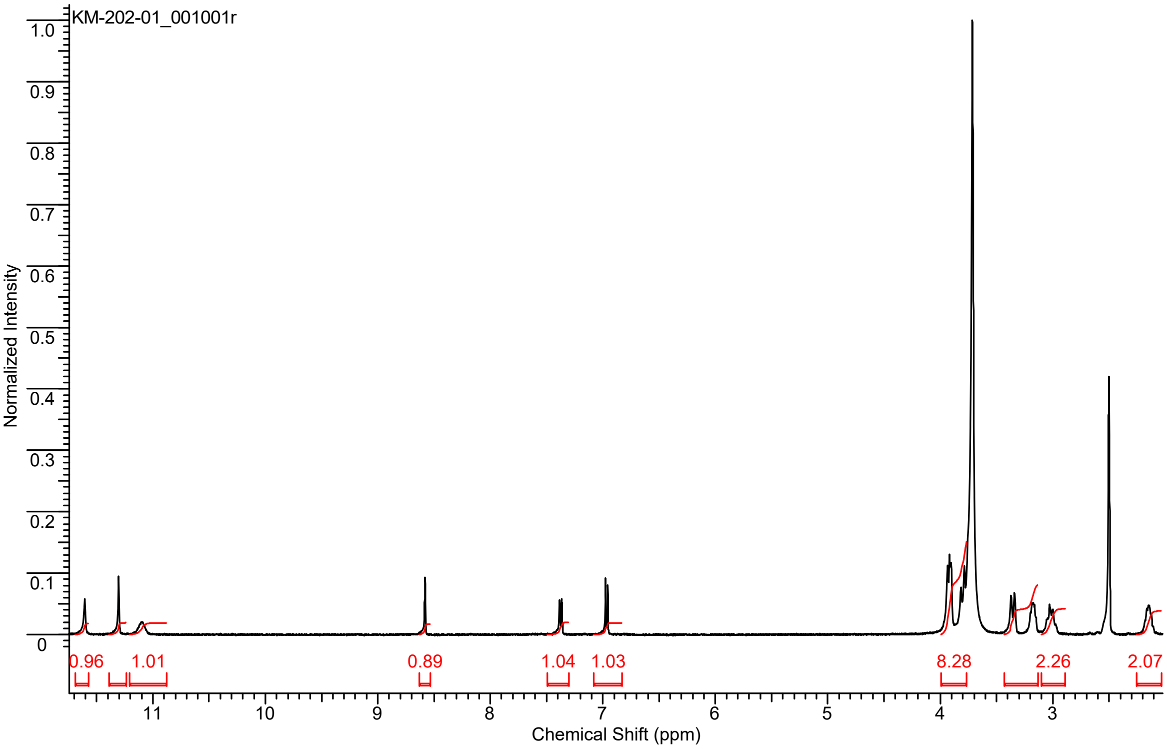

(Z)-3-(5-Oxo-1-phenyl-2-thioxoimidazolidin-4-ylidene)indolin-2-one (3w)

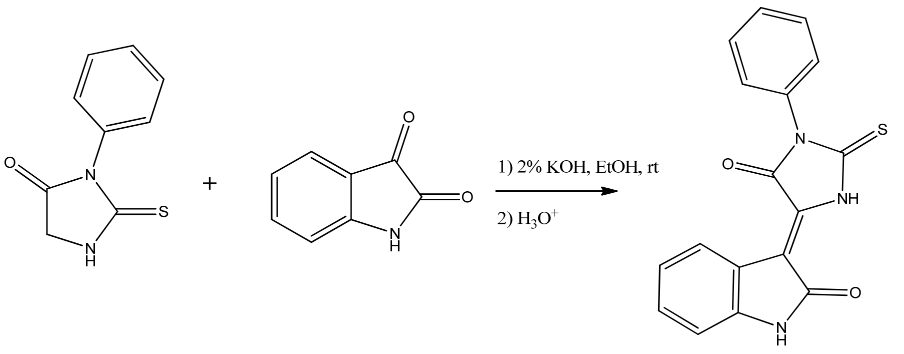

Chemical Formula: $C_{17}H_{11}N_3O_2S$
Molecular Weight: 321,3531

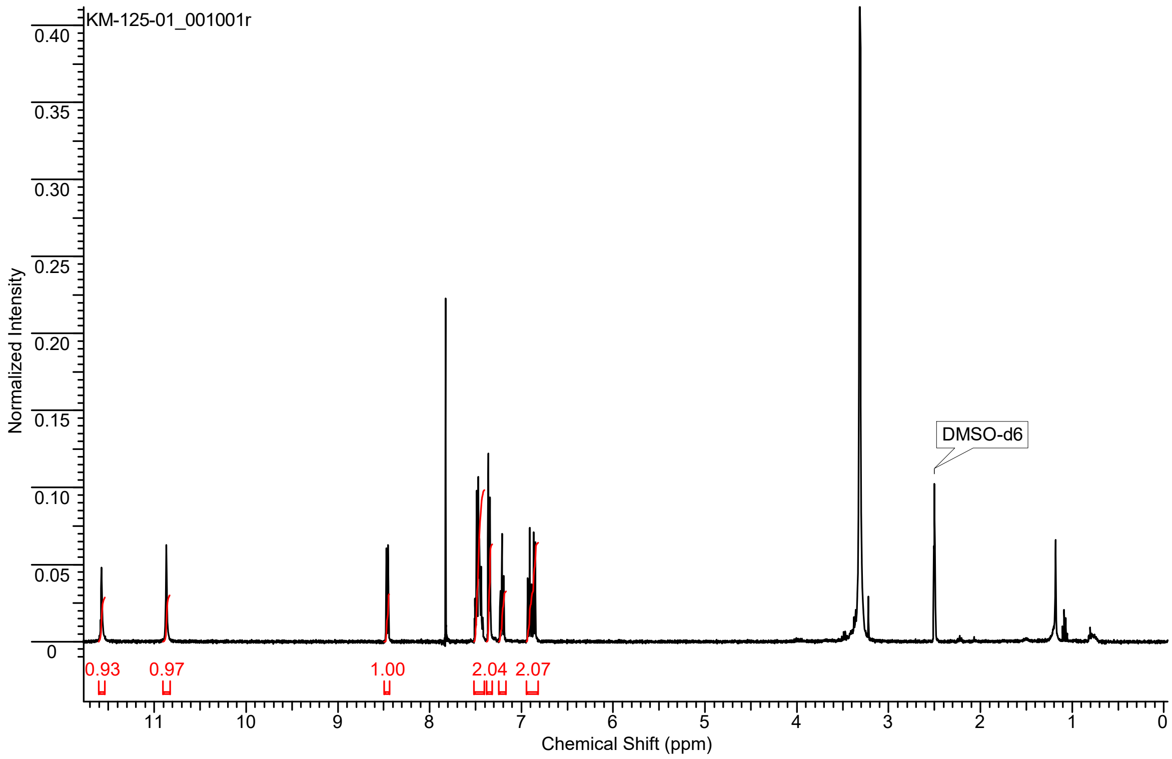

(Z)-5-Chloro-3-(5-oxo-1-phenyl-2-thioxoimidazolidin-4-ylidene)indolin-2-one (3x)

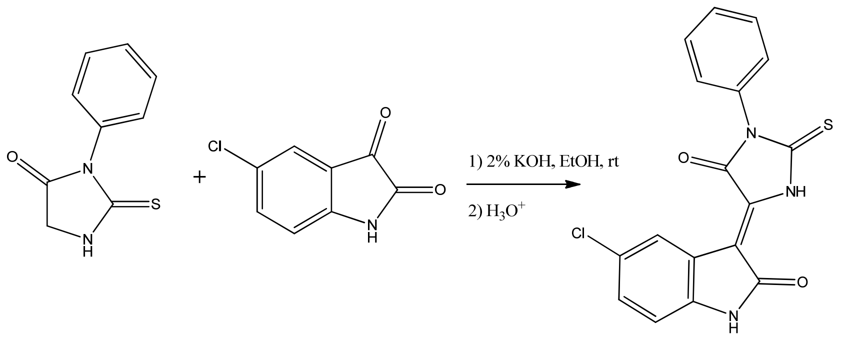

Chemical Formula: $C_{17}H_{10}ClN_3O_2S$
Molecular Weight: 355,7982

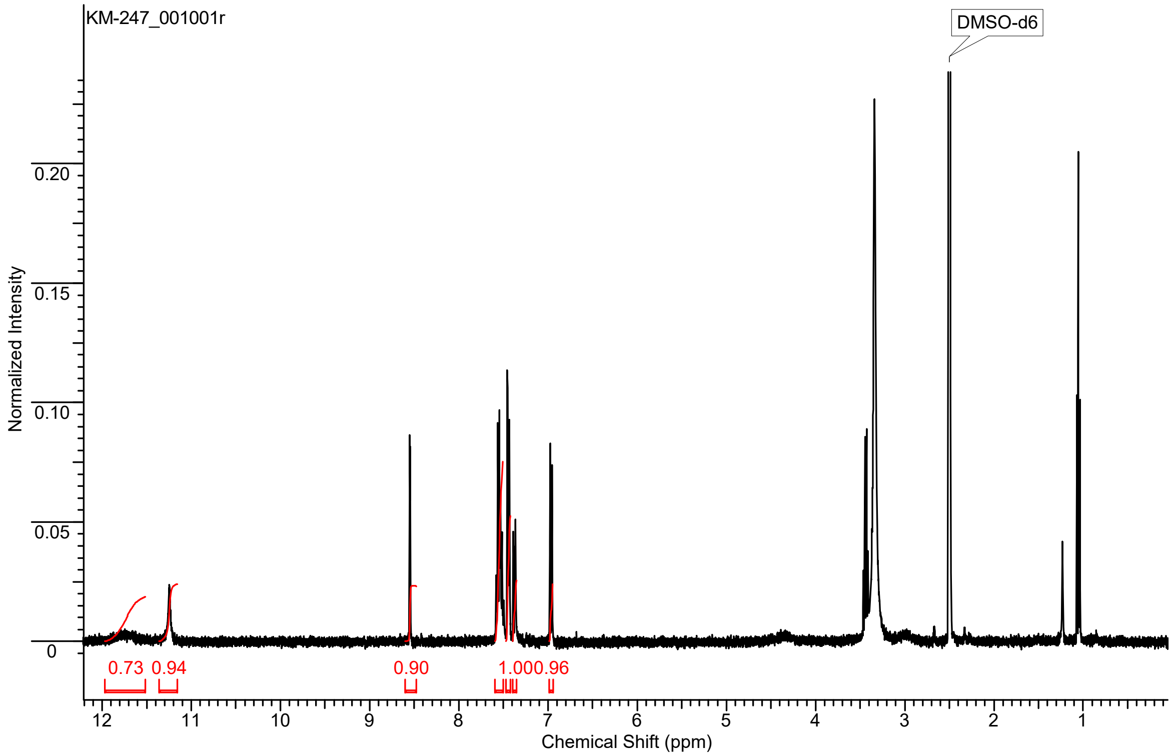

1'-Methyl-1-benzyl-2-thioxodispiro[imidazolidine-4,3'-pyrrolidine-4',3''-indoline]-2'',5-dione (4a)

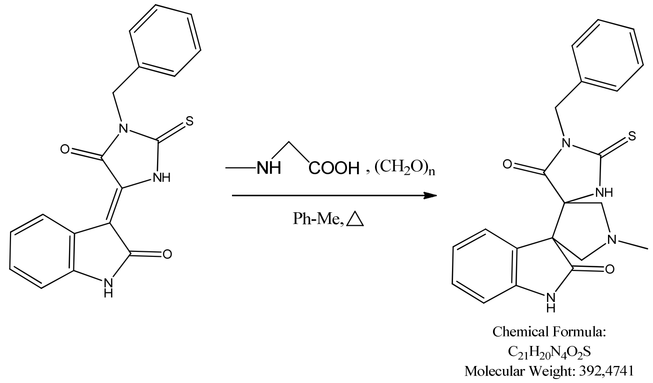

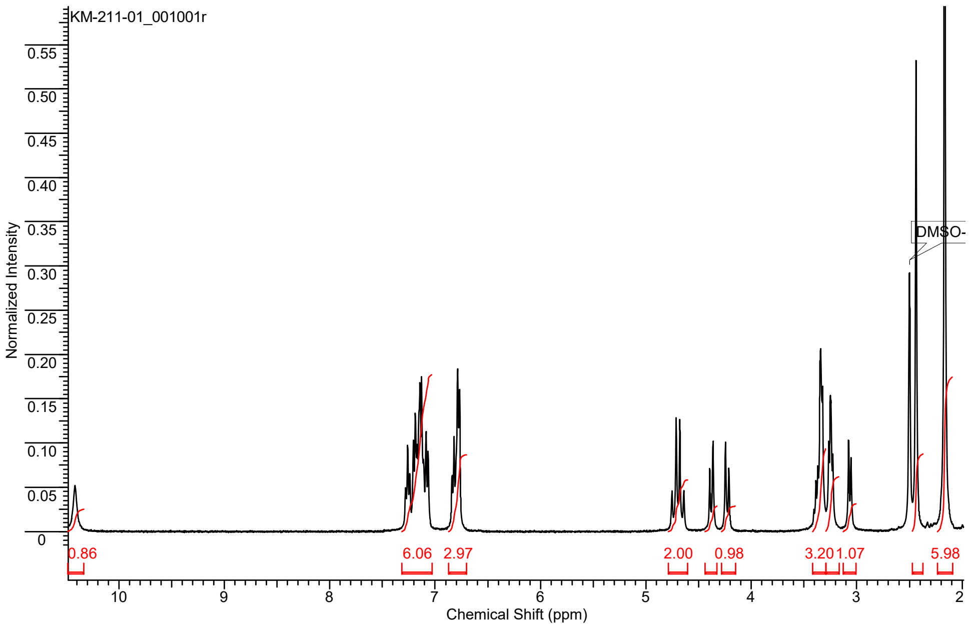

5''-Chloro-1'-methyl-1-benzyl-2-thioxodispiro[imidazolidine-4,3'-pyrrolidine-4',3''-indoline]-2'',5-dione (4b)

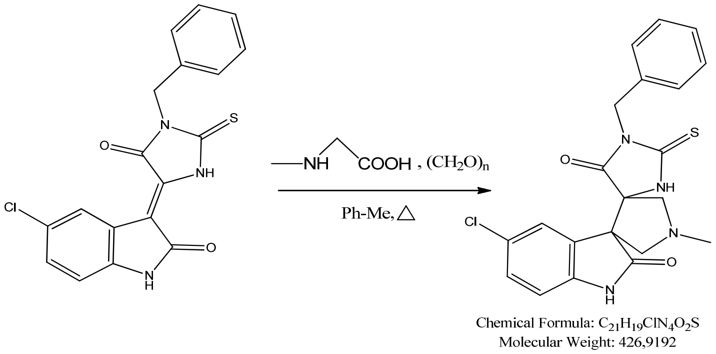

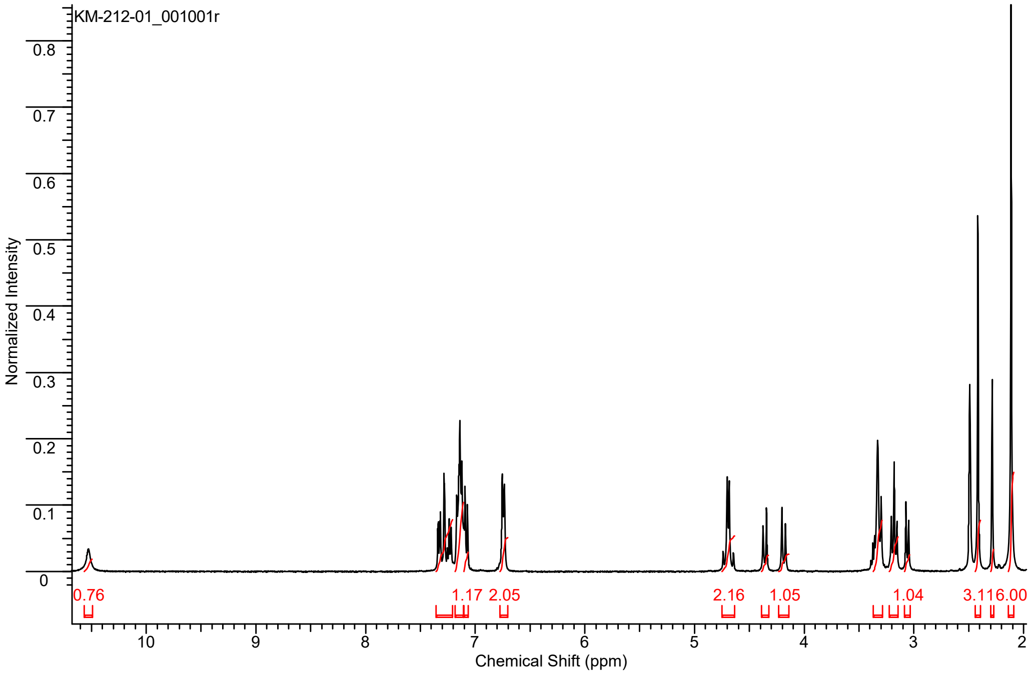

1'-Methyl-1-allyl-2-thioxodispiro[imidazolidine-4,3'-pyrrolidine-4',3''-indoline]-2'',5-dione (4c)

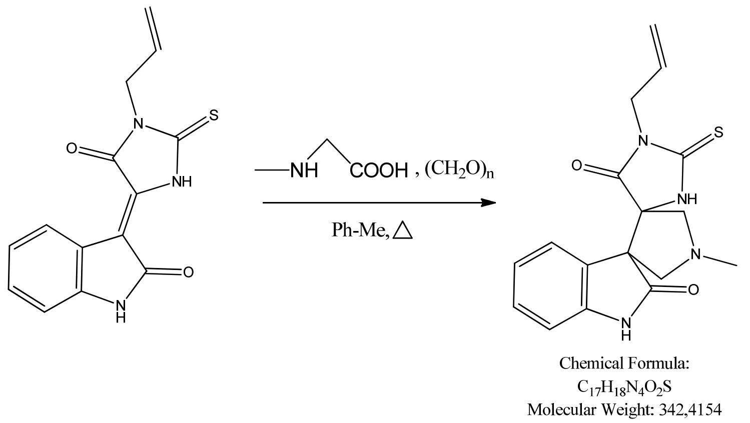

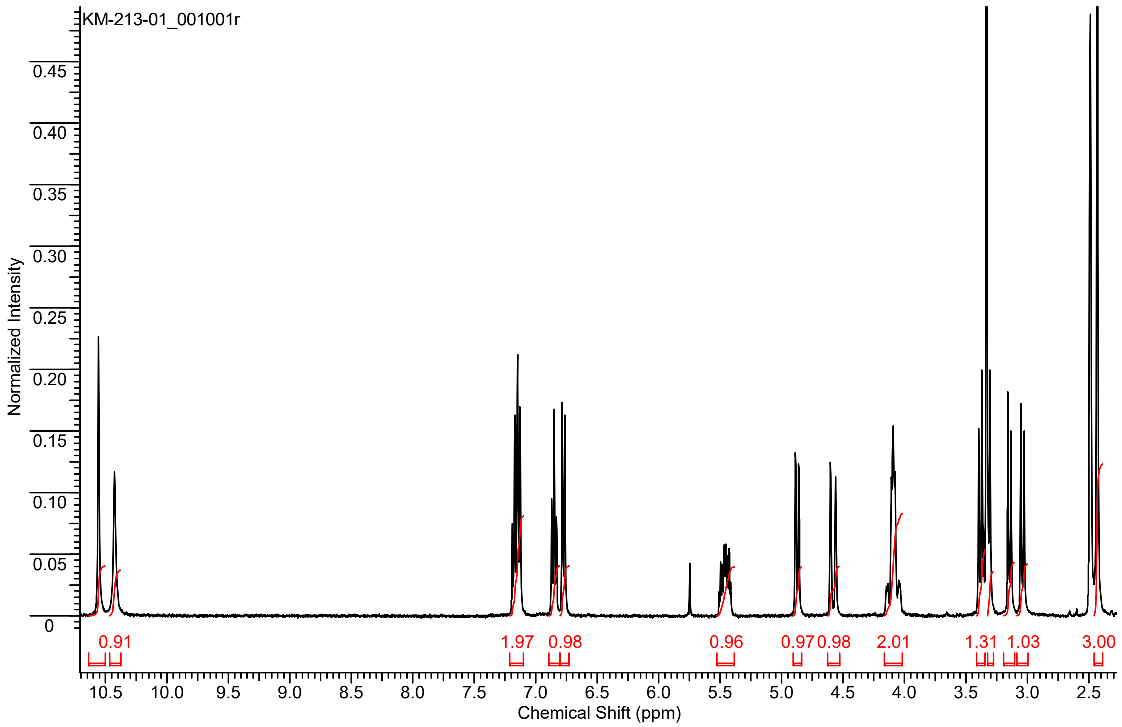

5''-Chloro-1'-methyl-1-allyl-2-thioxodispiro[imidazolidine-4,3'-pyrrolidine-4',3''-indoline]-2'',5-dione (4d)

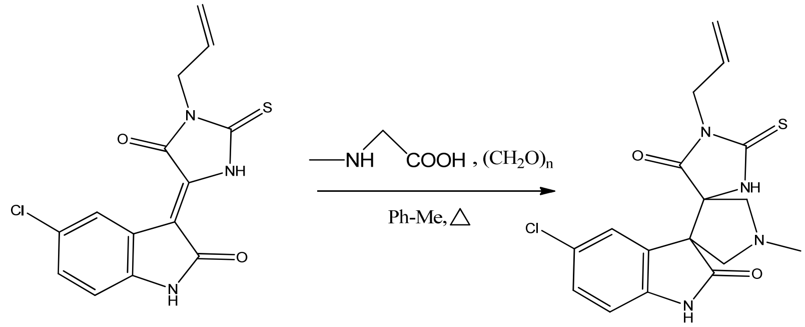

Chemical Formula: $C_{17}H_{17}ClN_4O_2S$
Molecular Weight: 376,8605

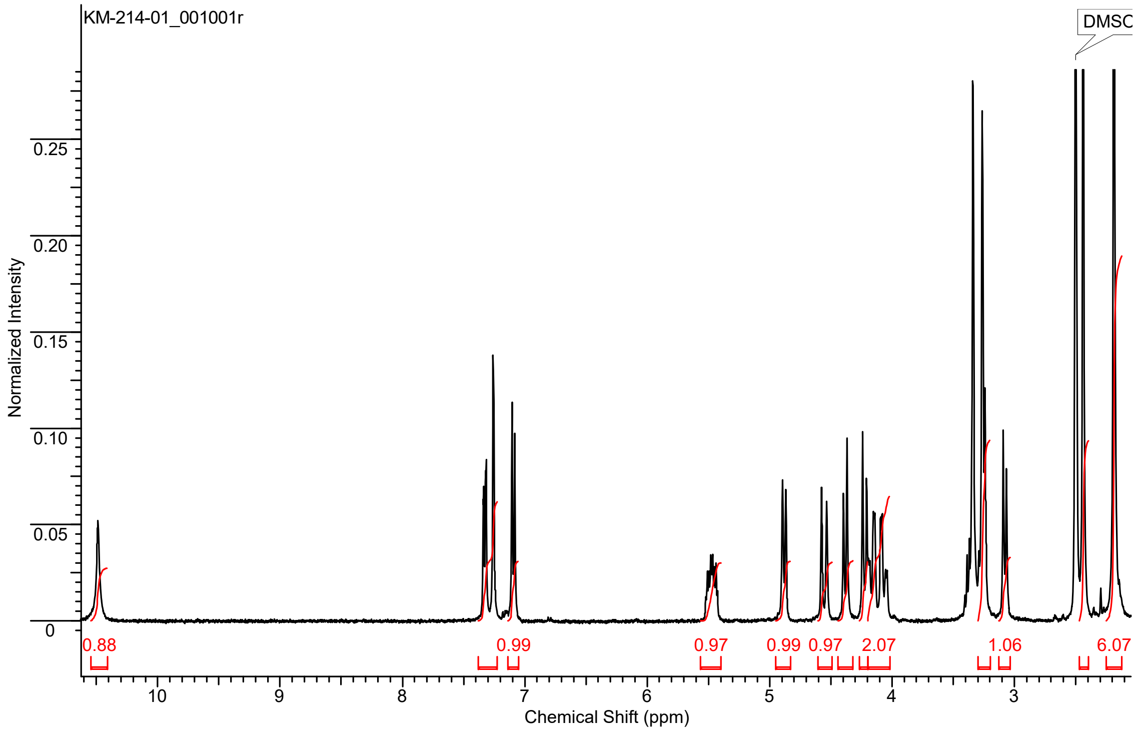

1'-Methyl-1-(4-methoxyphenyl)-2-thioxodispiro[imidazolidine-4,3'-pyrrolidine-4',3''-indoline]-2'',5-dione (4e)

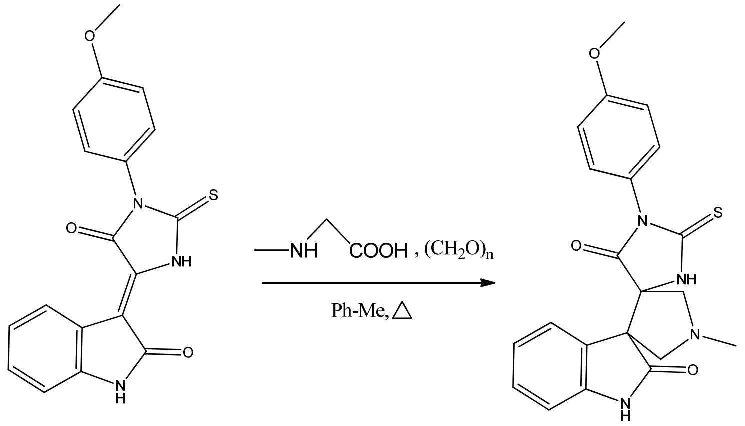

Chemical Formula: $C_{21}H_{20}N_4O_3S$
Molecular Weight: 408,4735

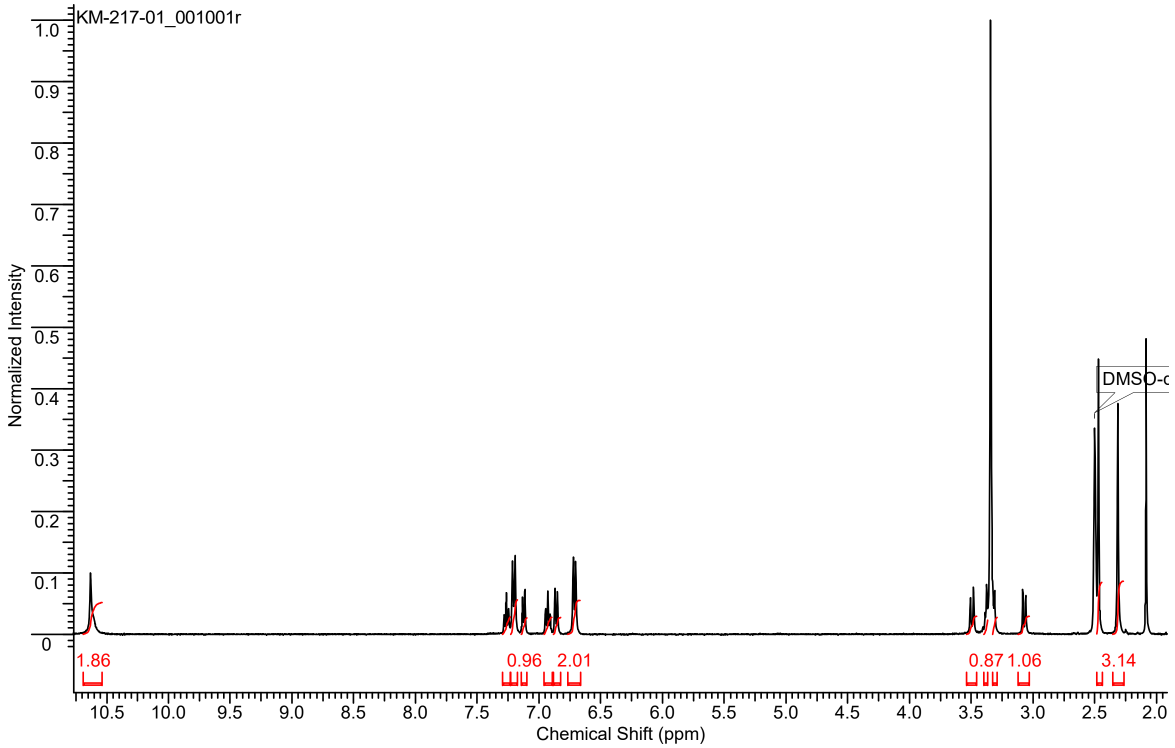

5''-Cchloro-1'-methyl-1-(4-methoxyphenyl)-2-thioxodispiro[imidazolidine-4,3'-pyrrolidine-4',3''-indoline]-2'',5-dione (4f)

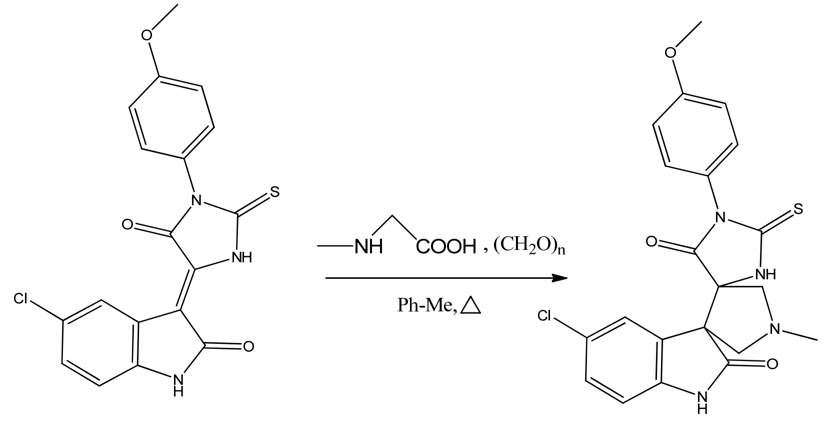

Chemical Formula: $C_{21}H_{19}ClN_4O_3S$
Molecular Weight: 442,9186

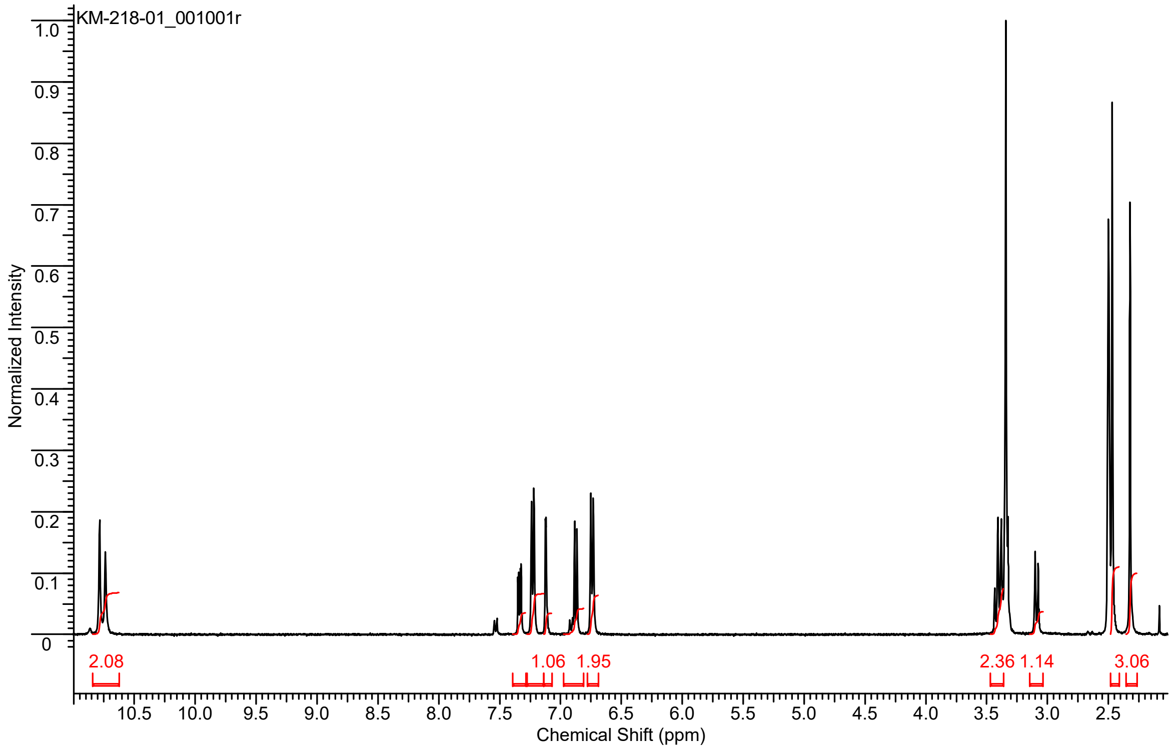

1'-Methyl-1-(4-ethoxyphenyl)-2-thioxodispiro[imidazolidine-4,3'-pyrrolidine-4',3''-indoline]-2'',5-dione (4g)

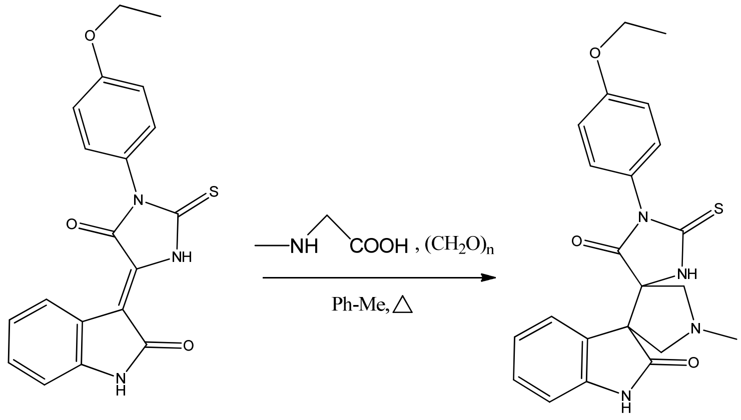

Chemical Formula: $C_{22}H_{22}N_4O_3S$
Molecular Weight: 422,5001

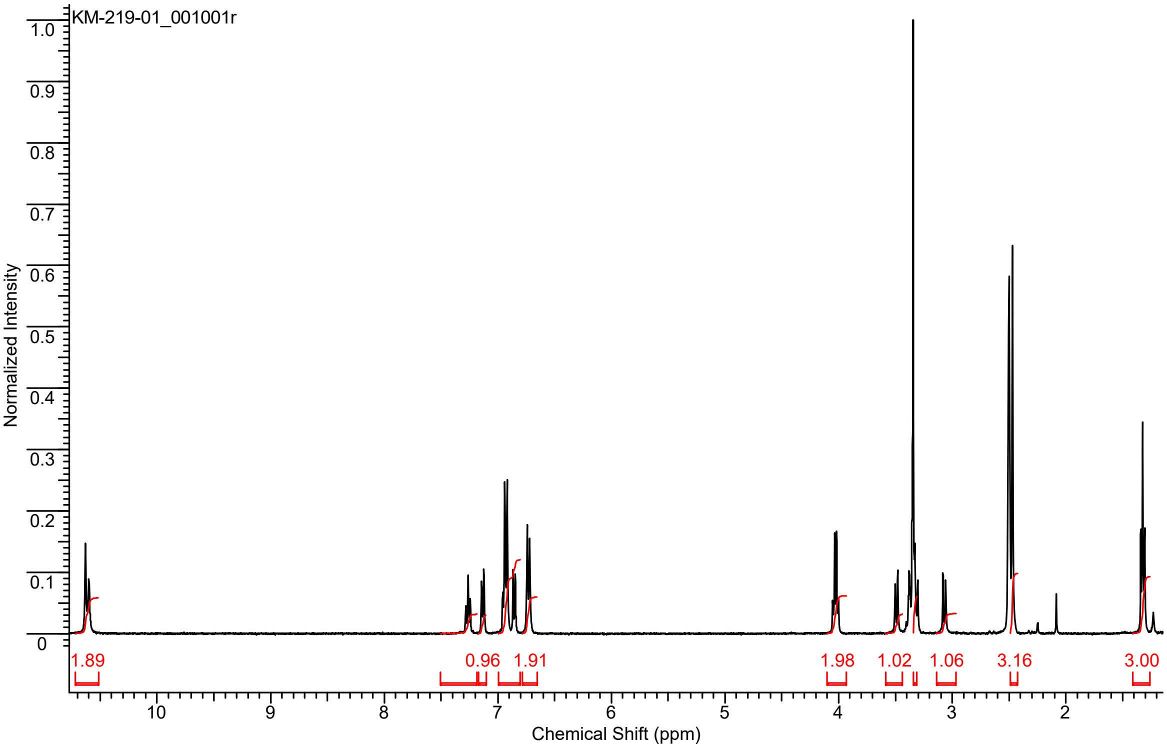

5''-Chloro-1'-methyl-1-(4-ethoxyphenyl)-2-thioxodispiro[imidazolidine-4,3'-pyrrolidine-4',3''-indoline]-2'',5-dione (4h)

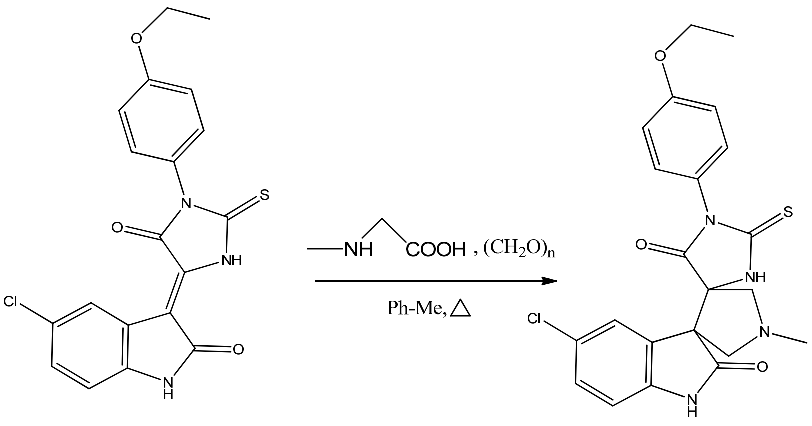

Chemical Formula: $C_{22}H_{21}ClN_4O_3S$
Molecular Weight: 456,9451

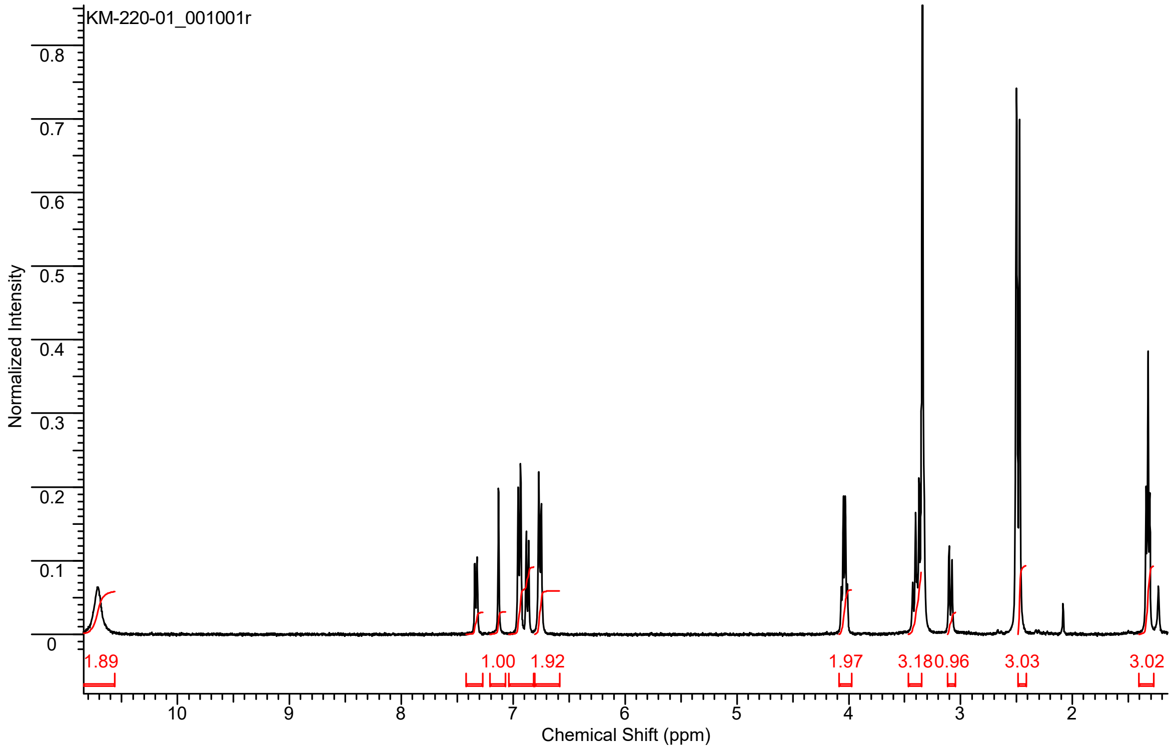

1'-Methyl-1-(p-tolyl)-2-thioxodispiro[imidazolidine-4,3'-pyrrolidine-4',3''-indoline]-2'',5-dione (4i)

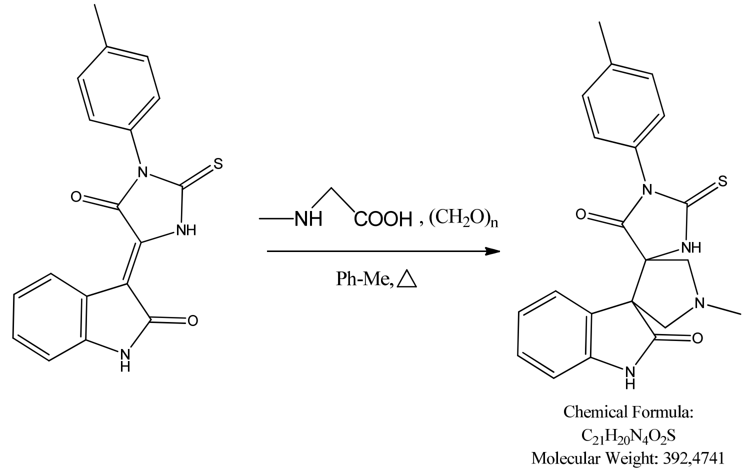

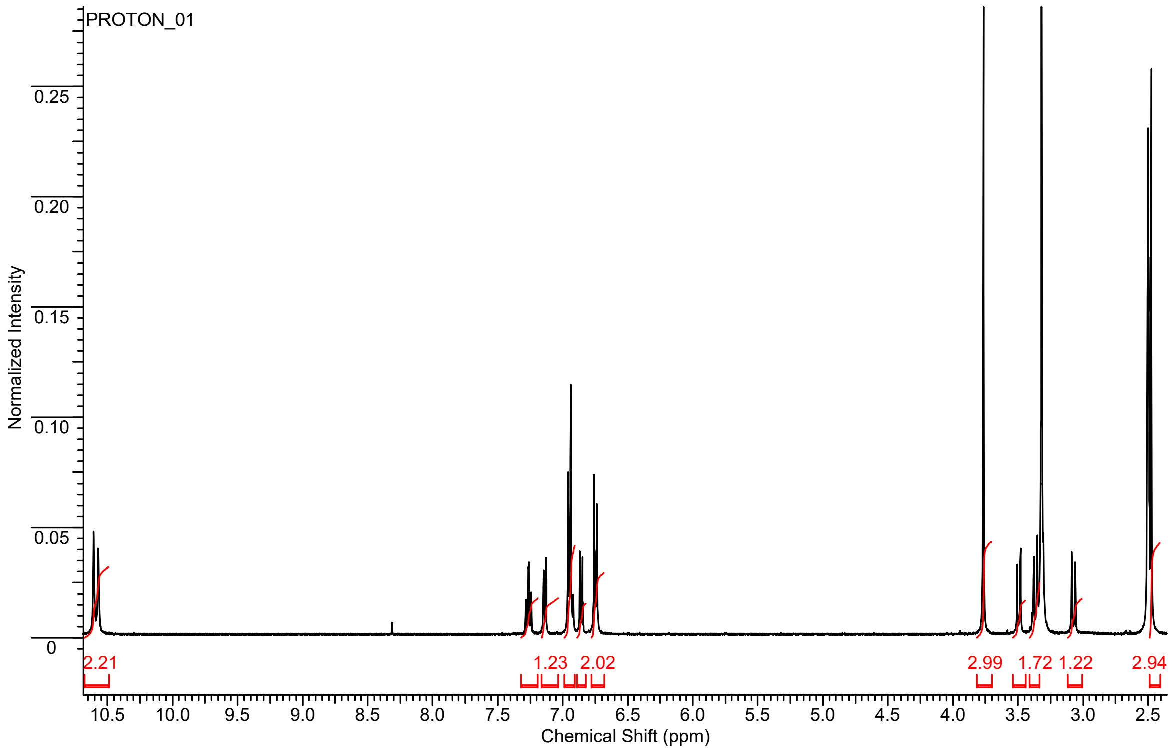

5''-Chloro-1'-methyl-1-(p-tolyl)-2-thioxodispiro[imidazolidine-4,3'-pyrrolidine-4',3''-indoline]-2'',5-dione (4j)

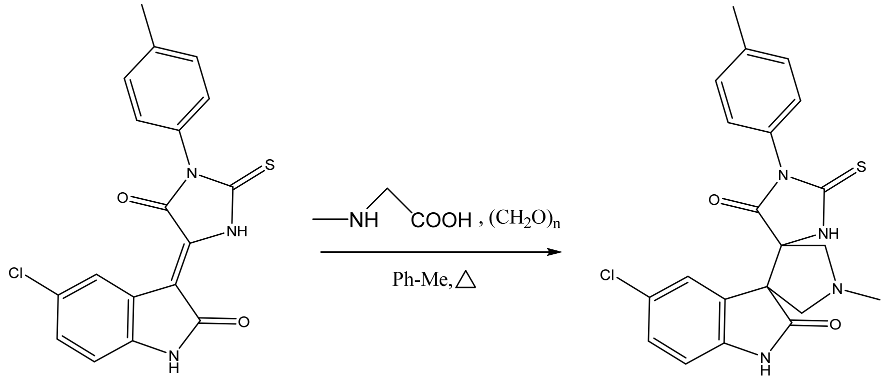

Chemical Formula: $C_{21}H_{19}ClN_4O_2S$
Molecular Weight: 426,9190

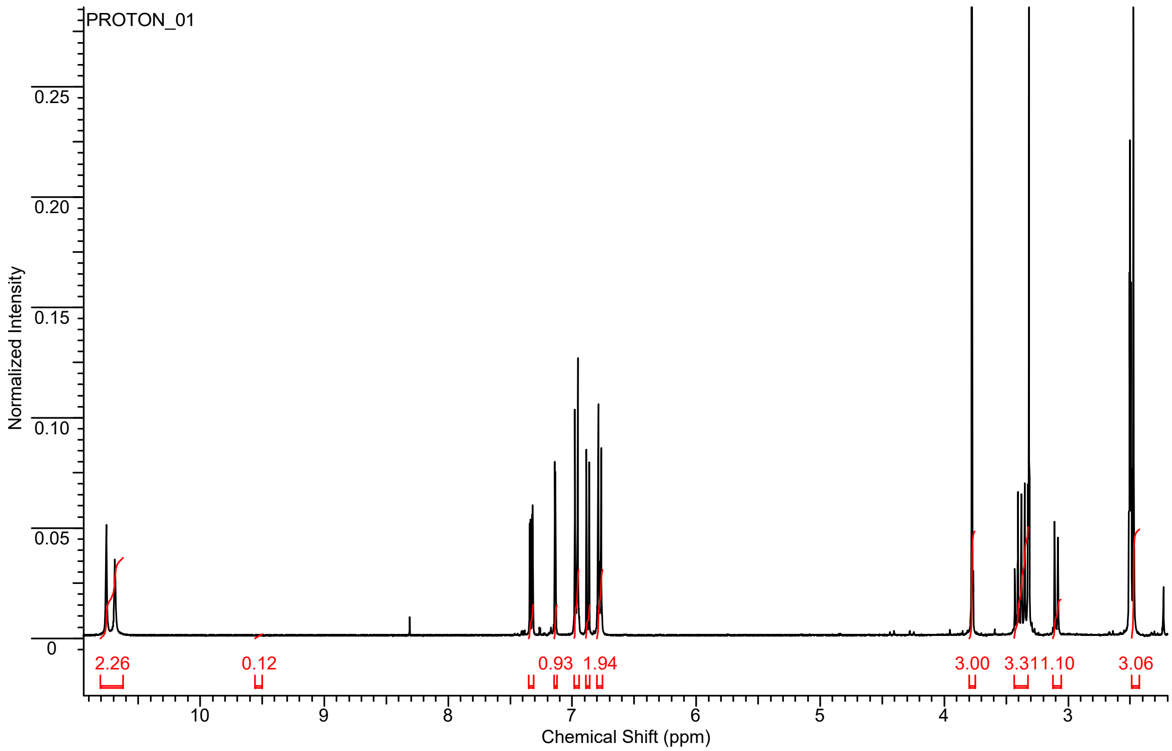

5''-Chloro-1'-methyl-1-(4-chlorophenyl)-2-thioxodispiro[imidazolidine-4,3'-pyrrolidine-4',3''-indoline]-2'',5-dione (4k)

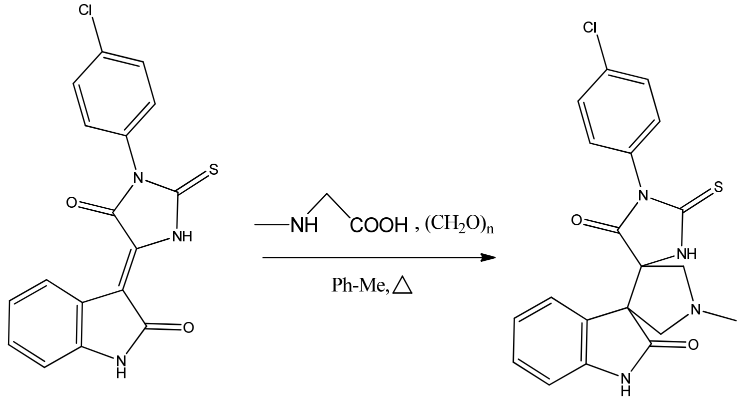

Chemical Formula: $C_{20}H_{17}ClN_4O_2S$

Molecular Weight: 412,8926

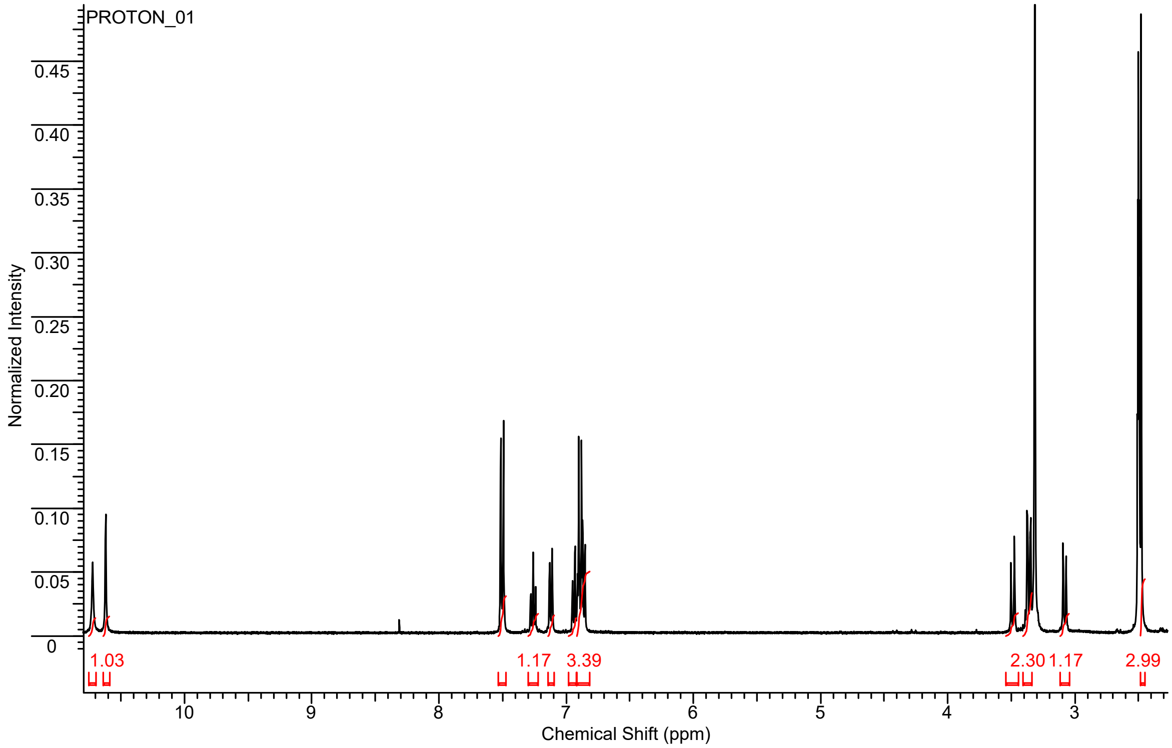

5''-Chloro-1'-methyl-1-(4-chlorophenyl)-2-thioxodispiro[imidazolidine-4,3'-pyrrolidine-4',3''-indoline]-2'',5-dione (4l)

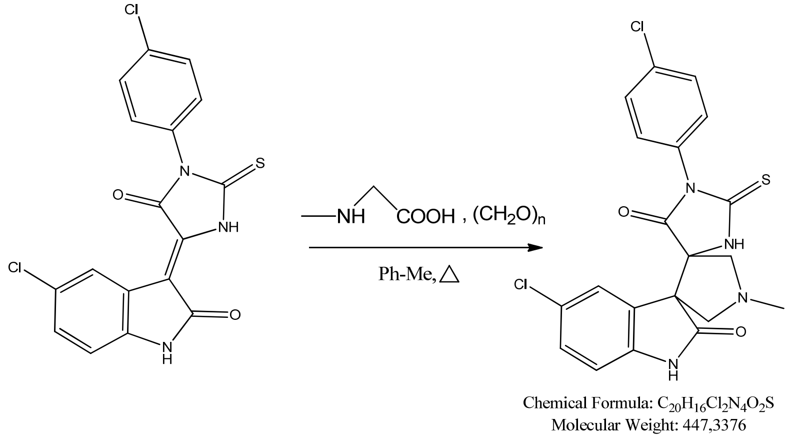

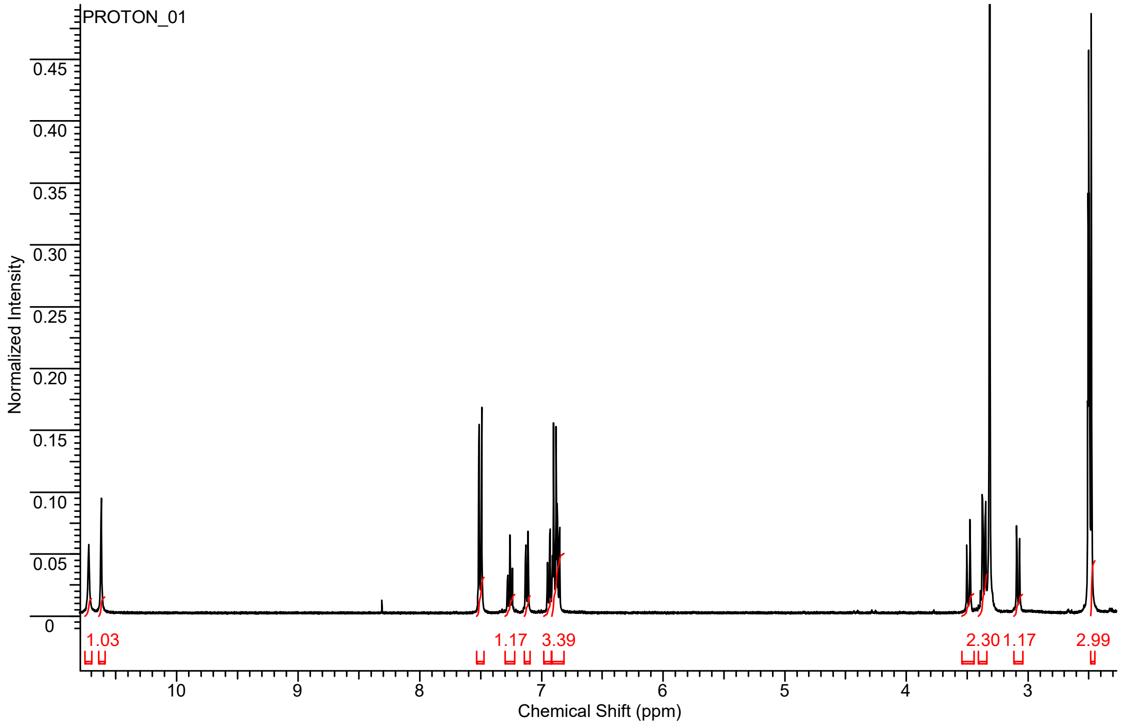

1'-Methyl-1-(4-fluorophenyl)-2-thioxodispiro[imidazolidine-4,3'-pyrrolidine-4',3''-indoline]-2'',5-dione (4m)

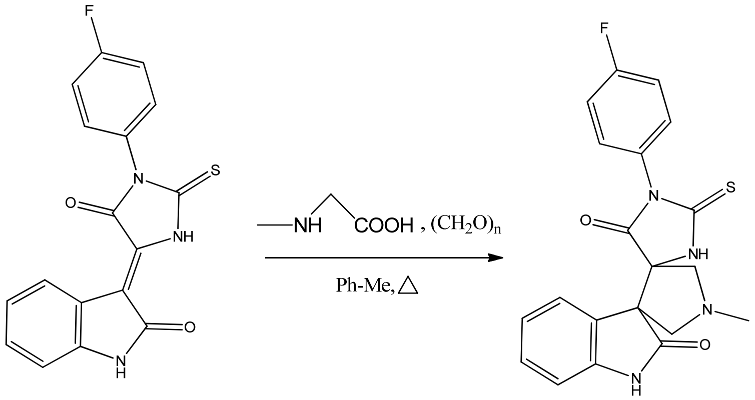

Chemical Formula: $C_{20}H_{17}FN_4O_2S$
Molecular Weight: 396,44

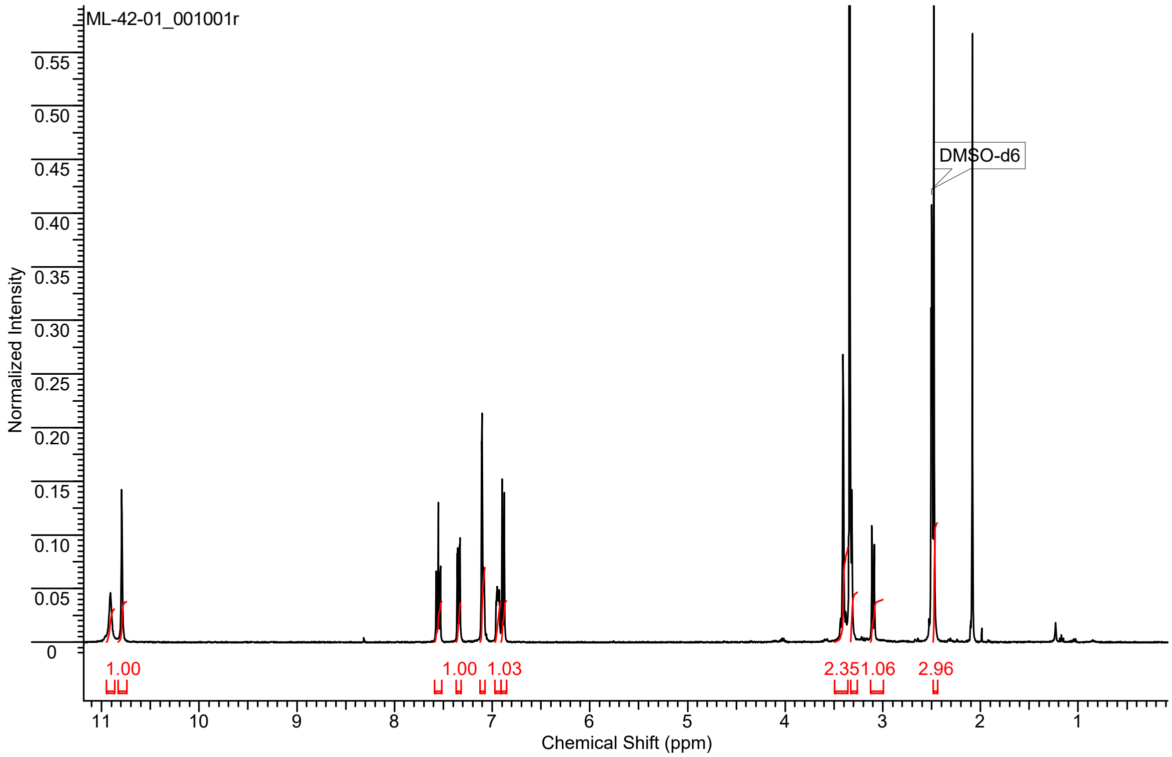

5''-Chloro-1'-methyl-1-(4-fluorophenyl)-2-thioxodispiro[imidazolidine-4,3'-pyrrolidine-4',3''-indoline]-2'',5-dione (4n)

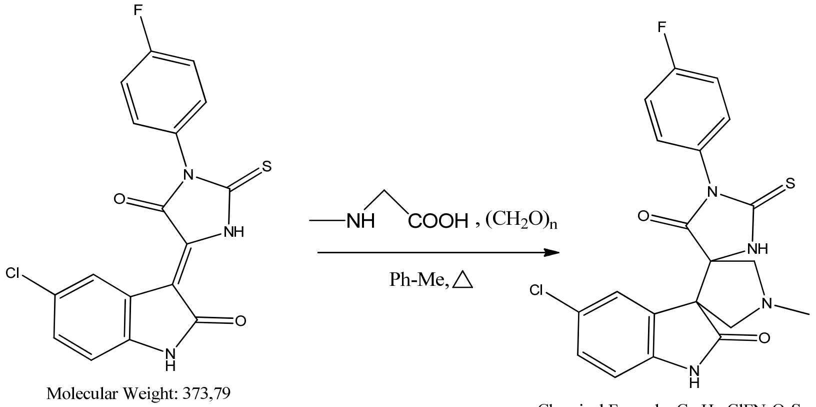

Molecular Weight: 373,79

Chemical Formula: $C_{20}H_{16}ClFN_4O_2S$
Molecular Weight: 430,88

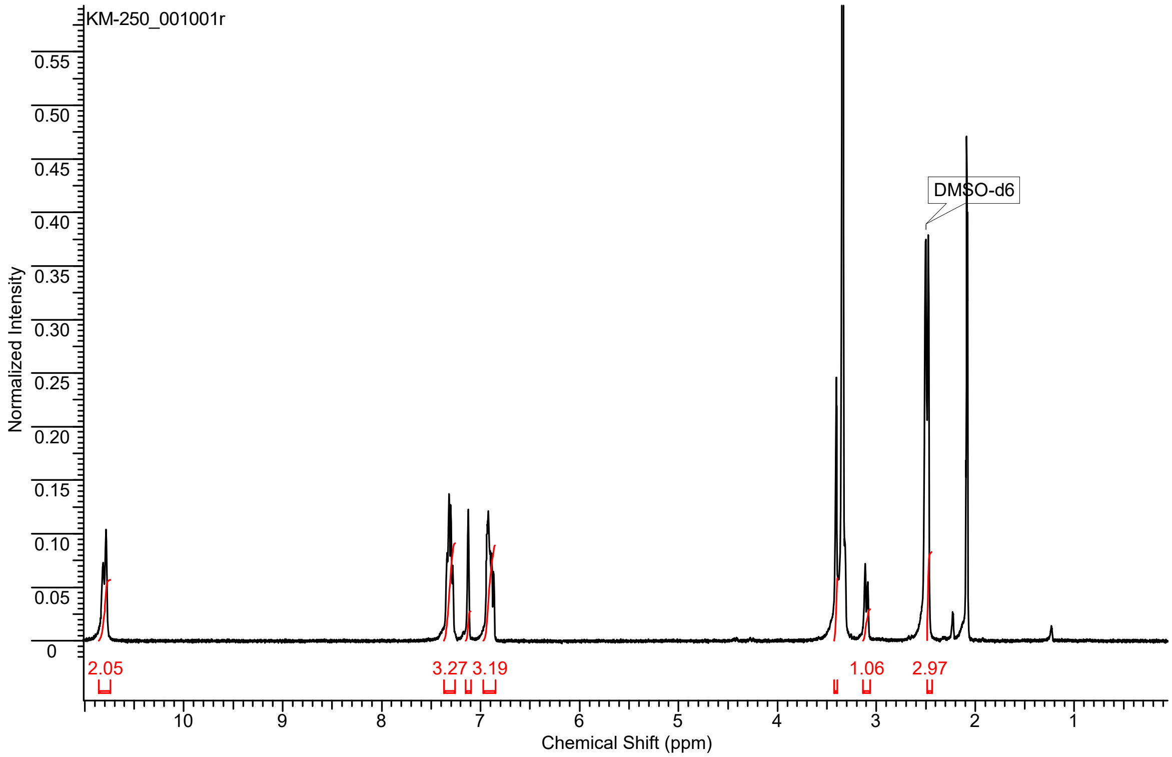

1'-Methyl-1-(3-chlorobenzyl)-2-thioxodispiro[imidazolidine-4,3'-pyrrolidine-4',3''-indoline]-2'',5-dione (4o)

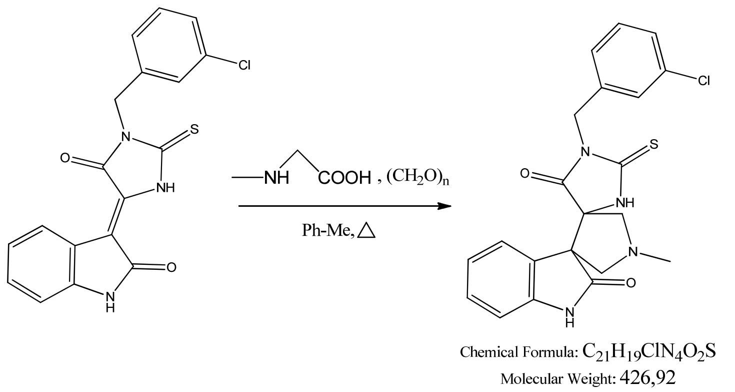

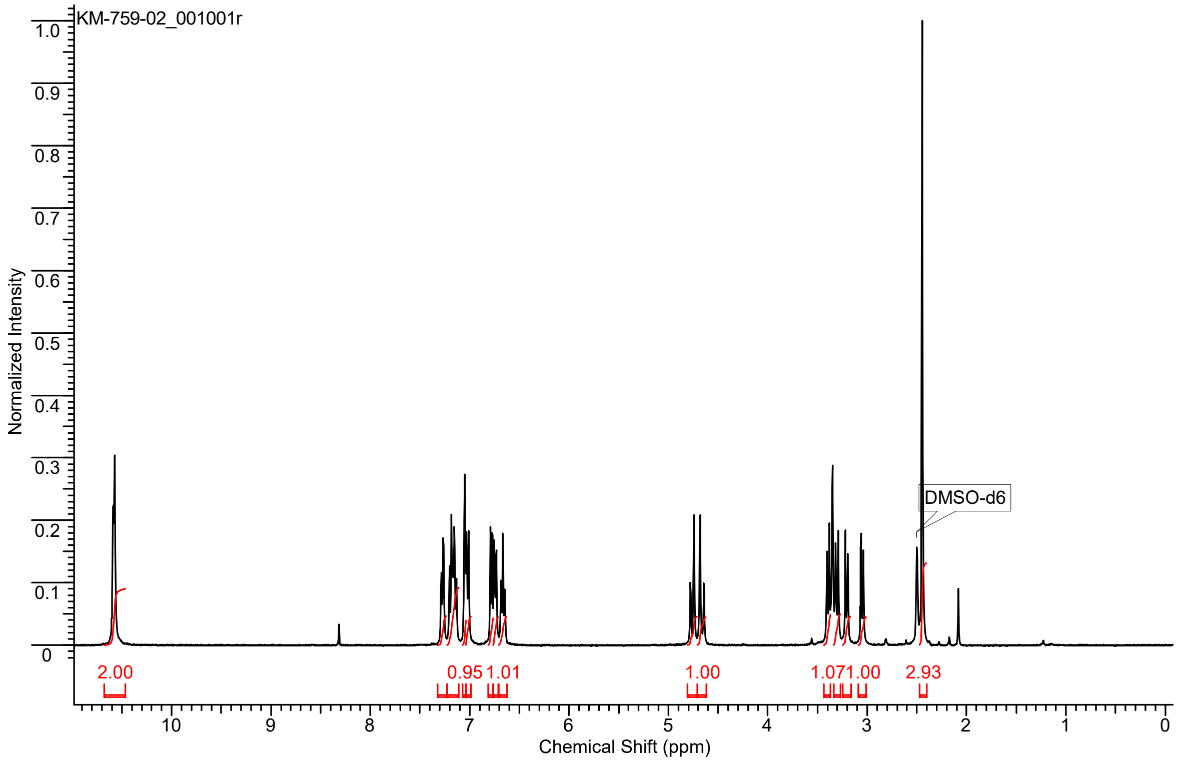

5''-Chloro-1'-methyl-1-(3-chlorobenzyl)-2-thioxodispiro[imidazolidine-4,3'-pyrrolidine-4',3''-indoline]-2'',5-dione (4p)

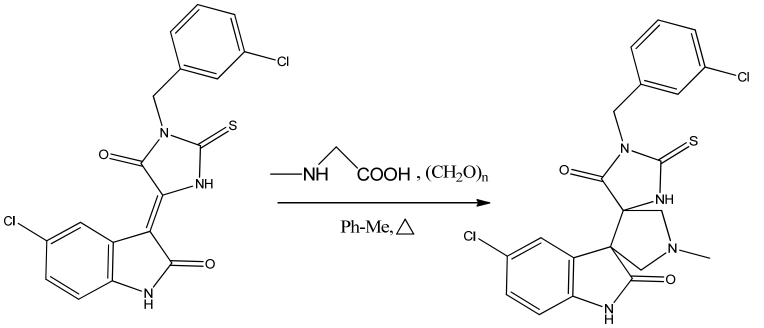

Chemical Formula: $C_{21}H_{18}Cl_2N_4O_2S$
Molecular Weight: 461,36

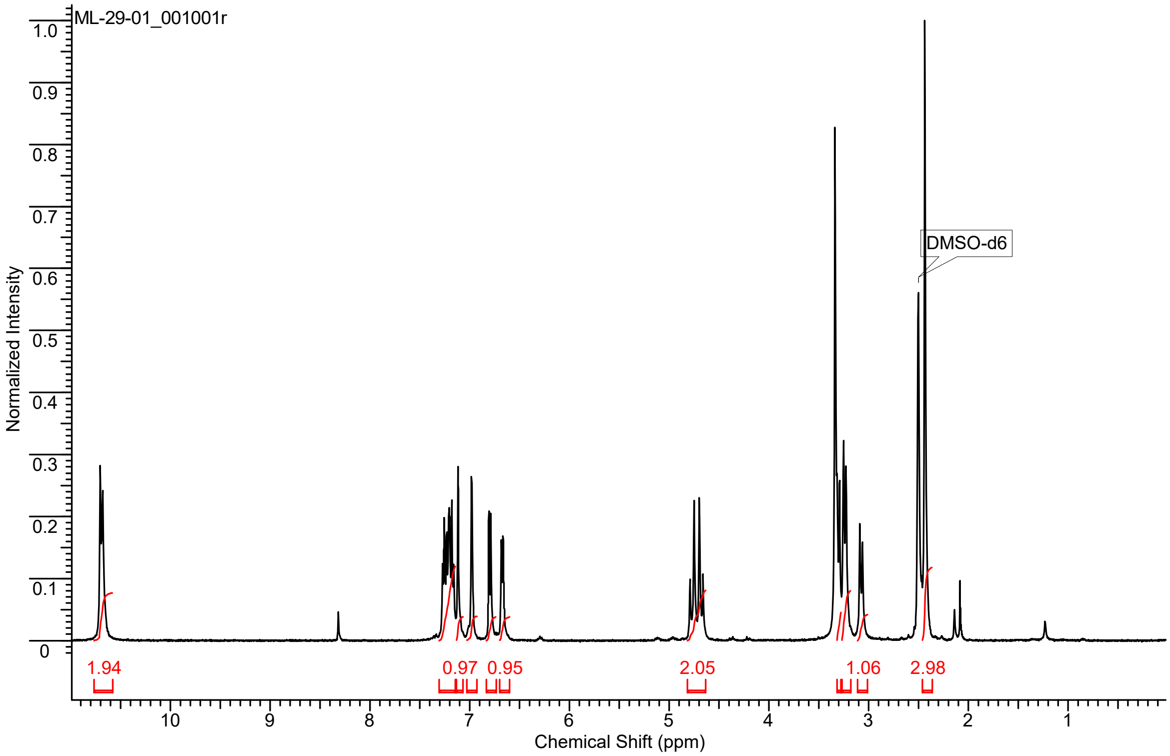

1'-Methyl-1-(3-chloro-4-fluorophenyl)-2-thioxodispiro[imidazolidine-4,3'-pyrrolidine-4',3''-indoline]-2'',5-dione(4q)

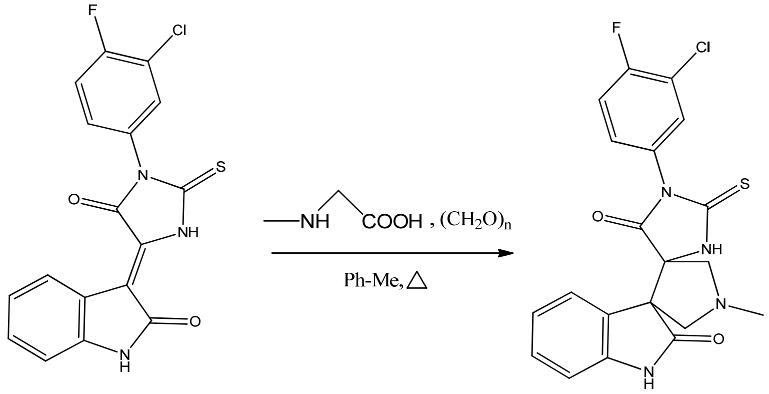

Chemical Formula: $C_{20}H_{16}ClFN_4O_2S$
Molecular Weight: 430,88

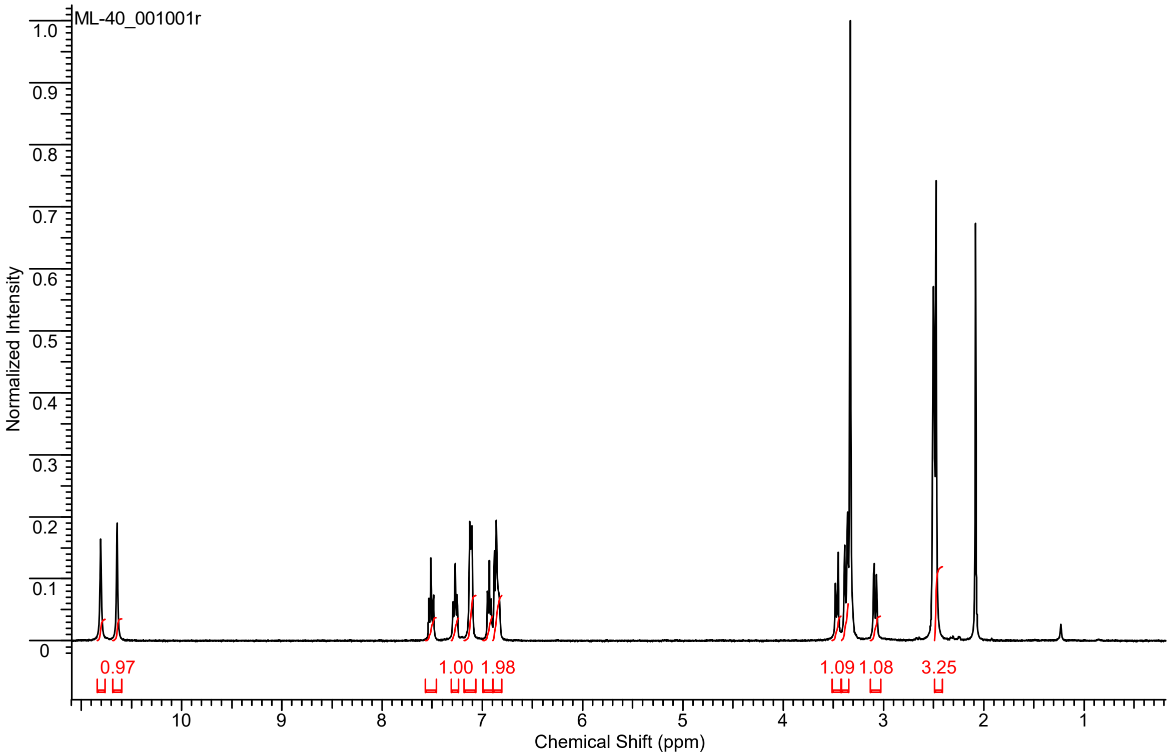

5''-Chloro-1'-methyl-1-(3-chloro-4-fluorophenyl)-2-thioxodispiro[imidazolidine-4,3'-pyrrolidine-4',3''-indoline]-2'',5-dione (4r)

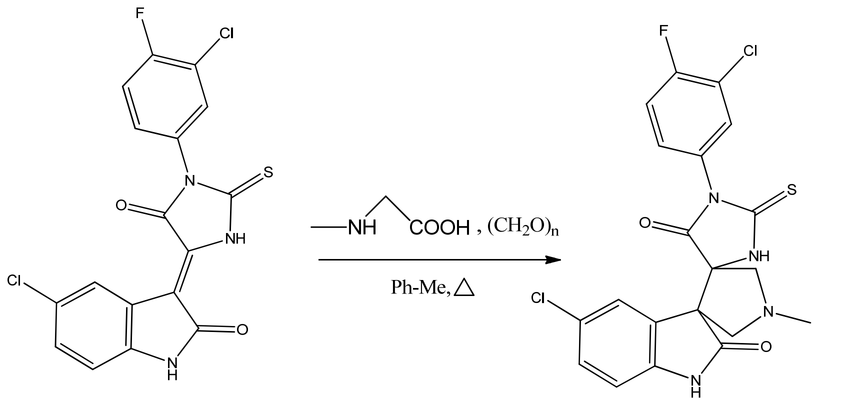

Chemical Formula: $C_{20}H_{15}Cl_2FN_4O_2S$
Molecular Weight: 465,33

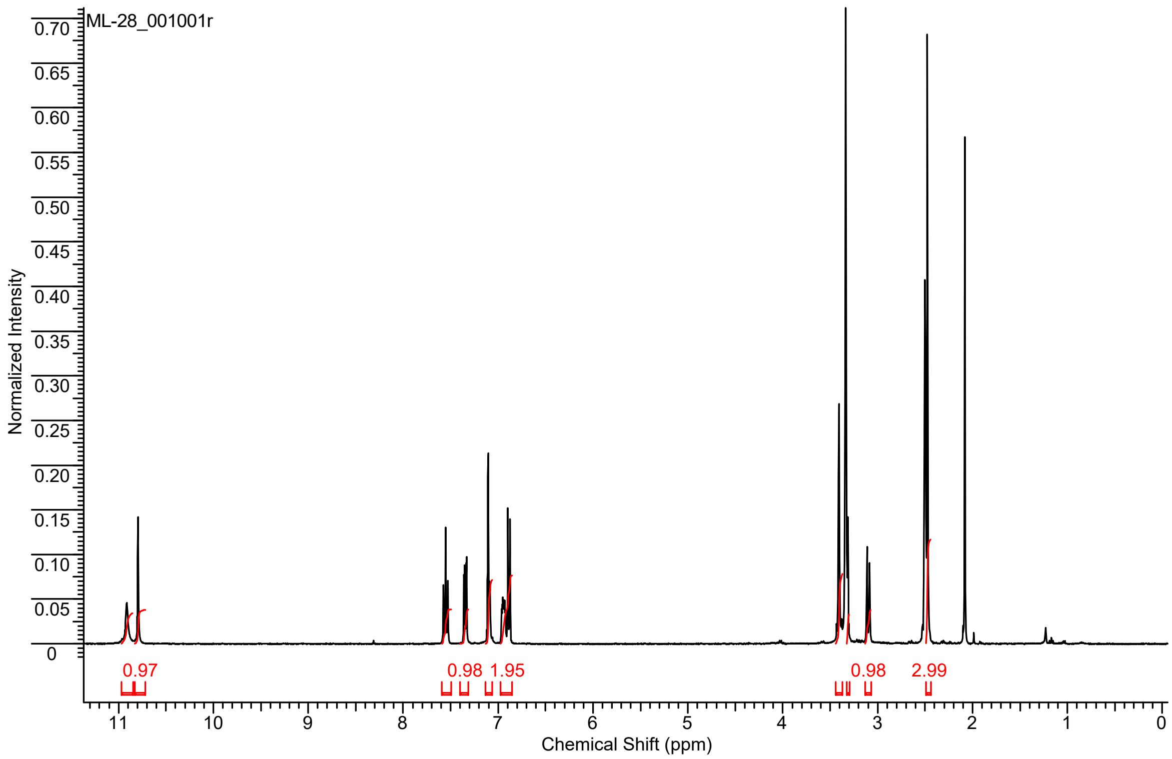

1'-Methyl-1-cyclopropyl-2-thioxodispiro[imidazolidine-4,3'-pyrrolidine-4',3''-indoline]-2'',5-dione (4s)

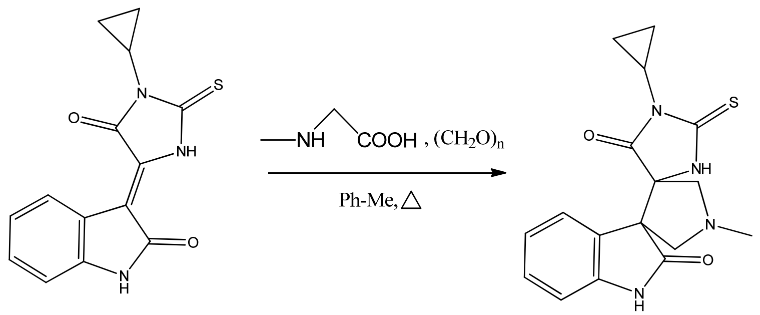

Chemical Formula: $C_{17}H_{18}N_4O_2S$
Molecular Weight: 342,4154

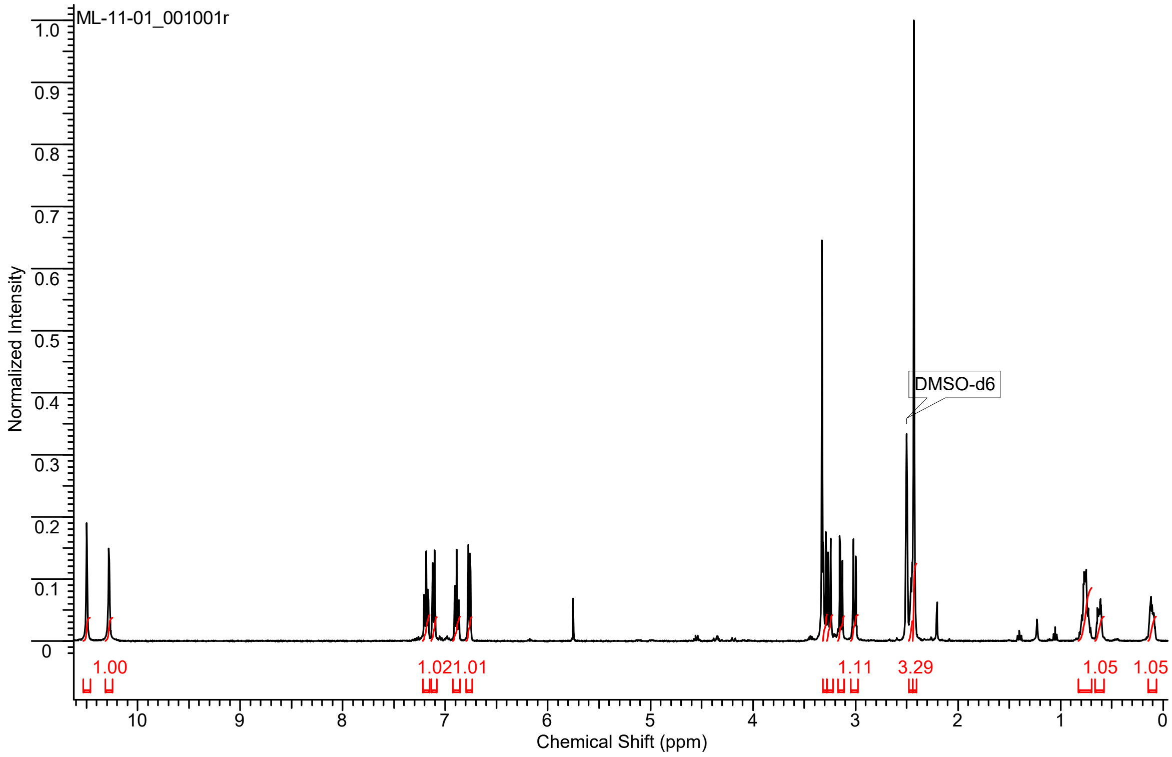

5''-Chloro-1'-methyl-1-cyclopropyl-2-thioxodispiro[imidazolidine-4,3'-pyrrolidine-4',3''-indoline]-2'',5-dione (4t)

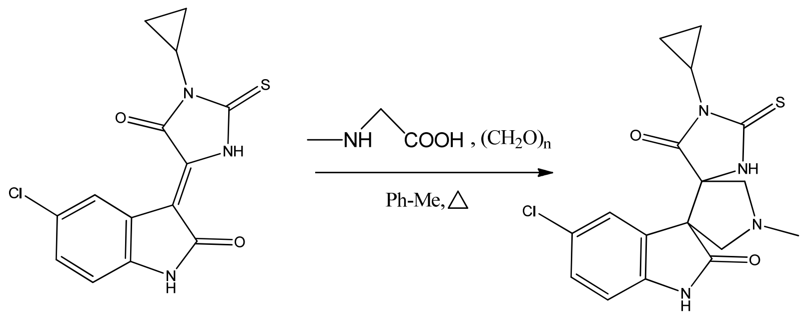

Chemical Formula: $C_{17}H_{17}ClN_4O_2S$
Molecular Weight: 376,8605

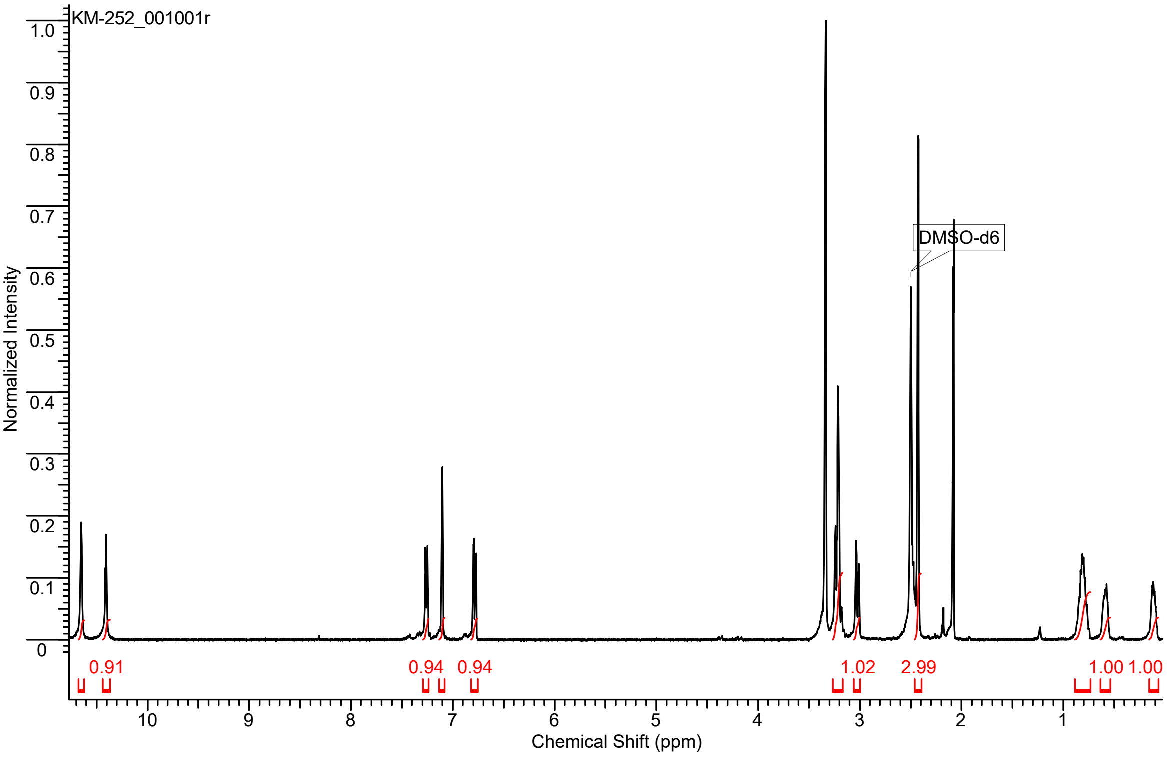

1'-Methyl-1-(3-morpholinopropyl)-2-thioxodispiro[imidazolidine-4,3'-pyrrolidine-4',3''-indoline]-2'',5-dione (4u)

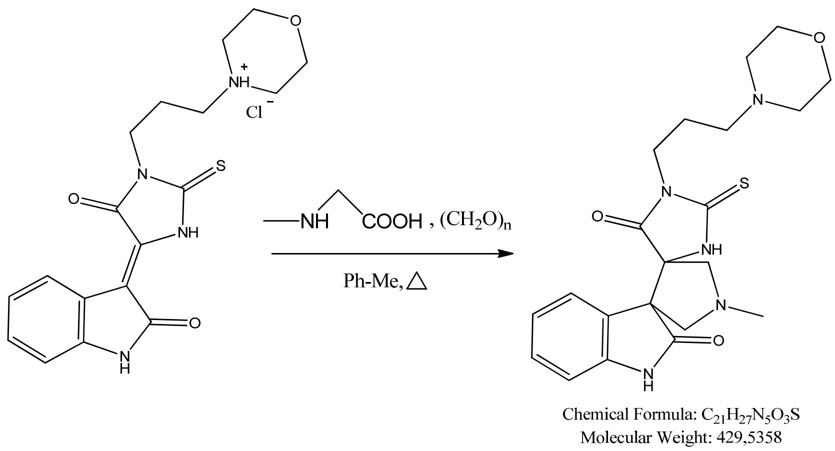

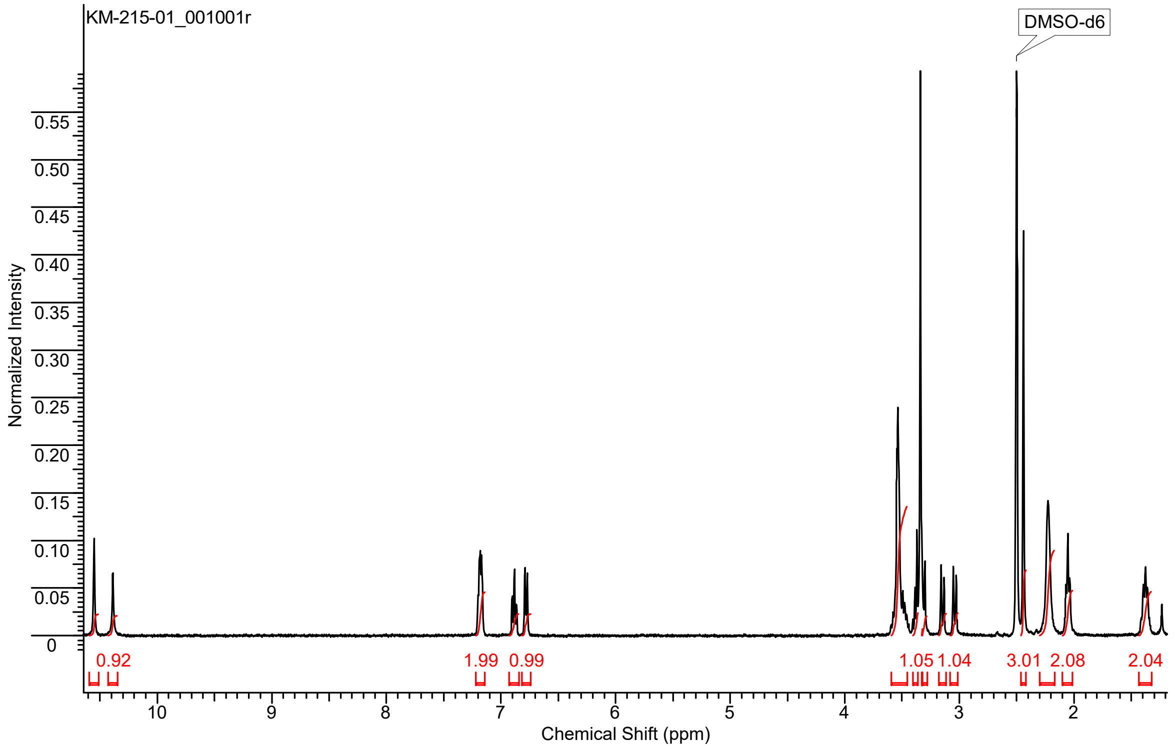

5''-Chloro-1'-methyl-1-(3-morpholinopropyl)-2-thioxodispiro[imidazolidine-4,3'-pyrrolidine-4',3''-indoline]-2'',5-dione (4v)

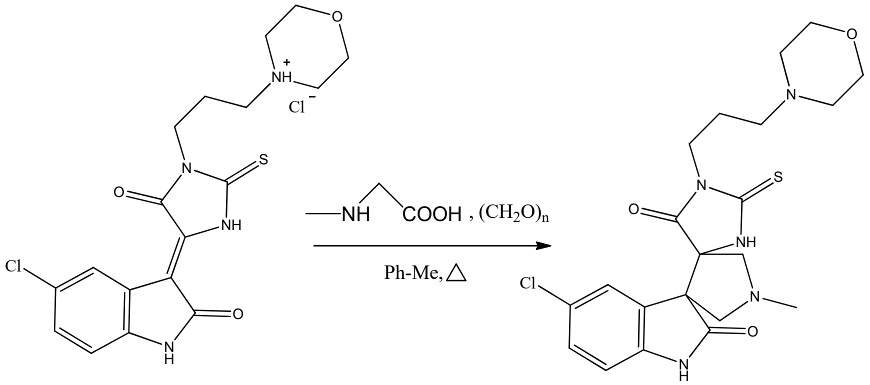

Chemical Formula: $C_{21}H_{26}ClN_5O_3S$
Molecular Weight: 463,9808

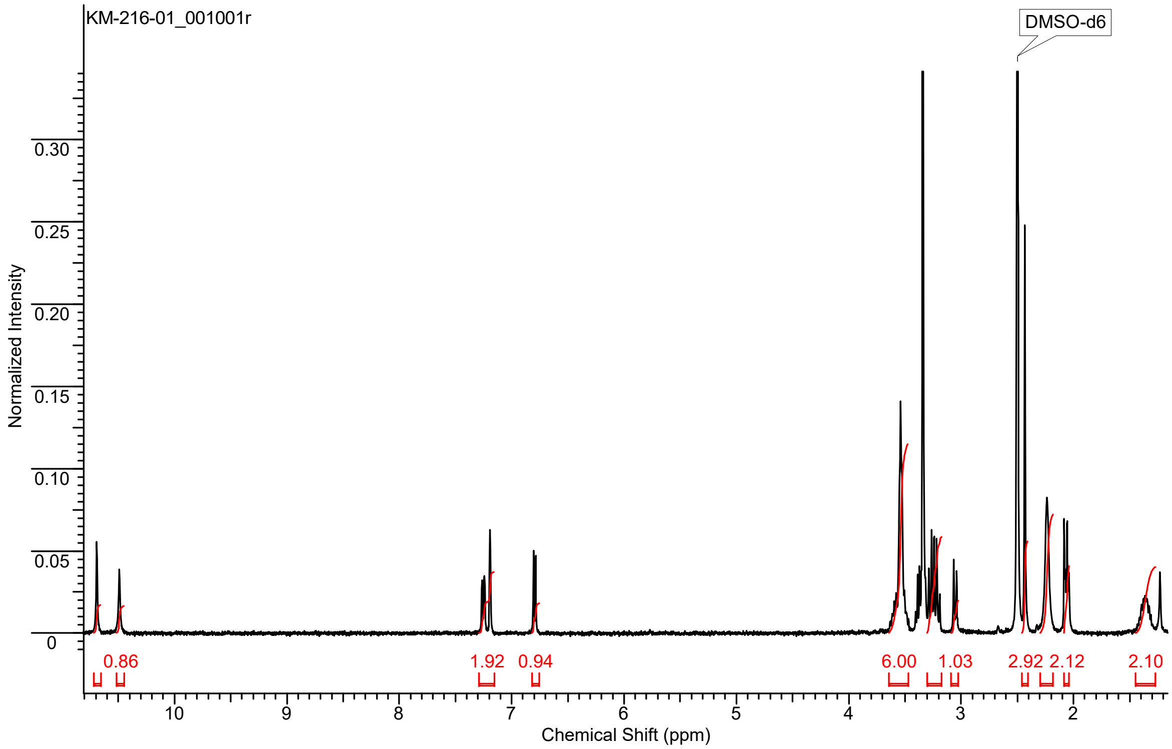

1'-Methyl-1-phenyl-2-thioxodispiro[imidazolidine-4,3'-pyrrolidine-4',3''-indoline]-2'',5-dione(4w)

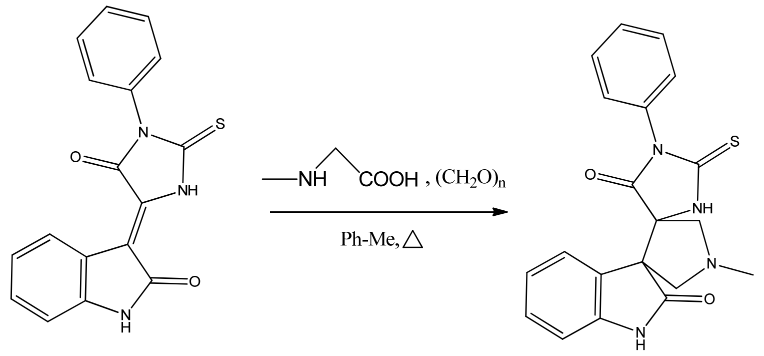

Chemical Formula: $C_{20}H_{18}N_4O_2S$
Molecular Weight: 378,4475

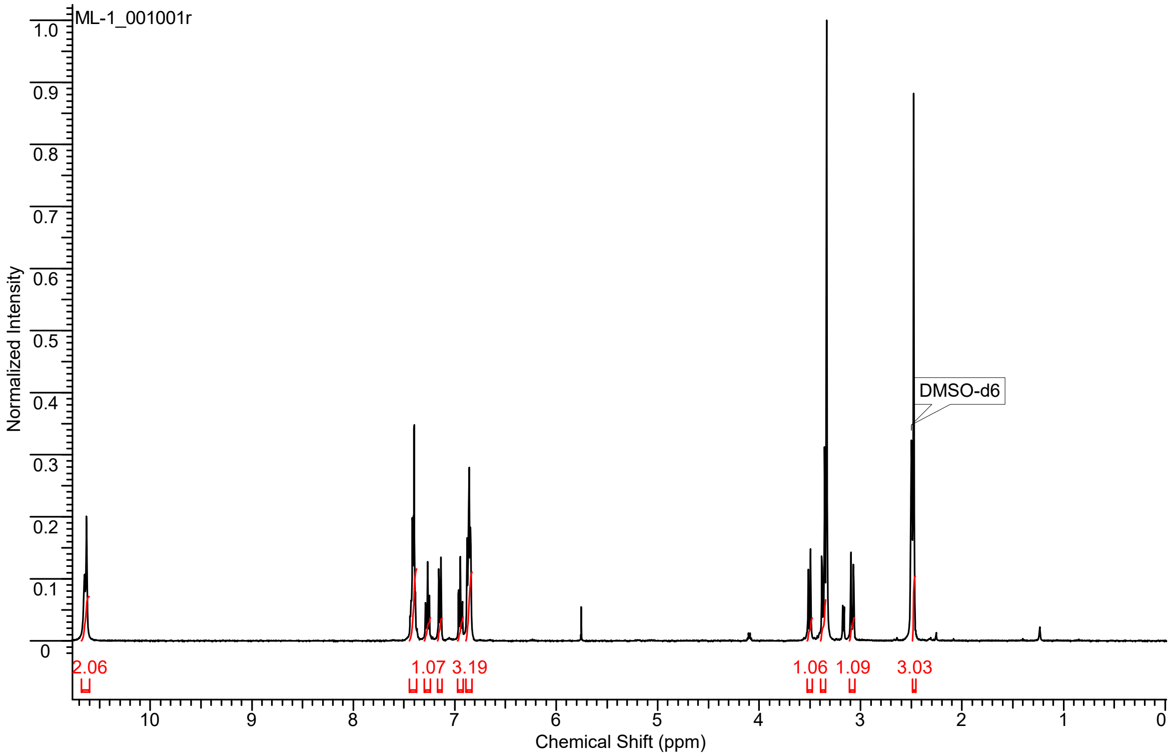

5''-Chloro-1'-methyl-1-phenyl-2-thioxodispiro[imidazolidine-4,3'-pyrrolidine-4',3''-indoline]-2'',5-dione (4x)

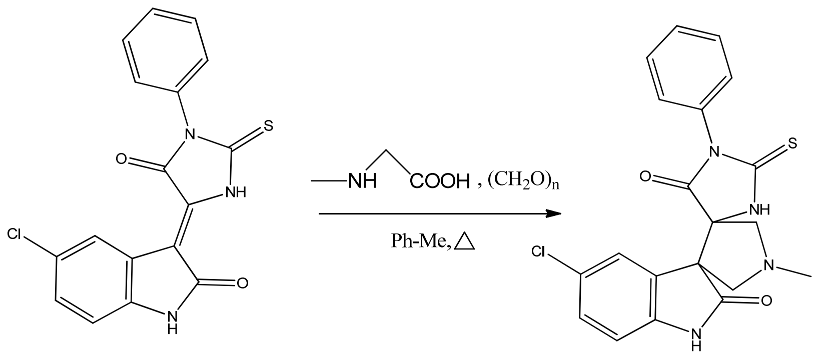

Chemical Formula: $C_{20}H_{17}ClN_4O_2S$
Molecular Weight: 412,8926

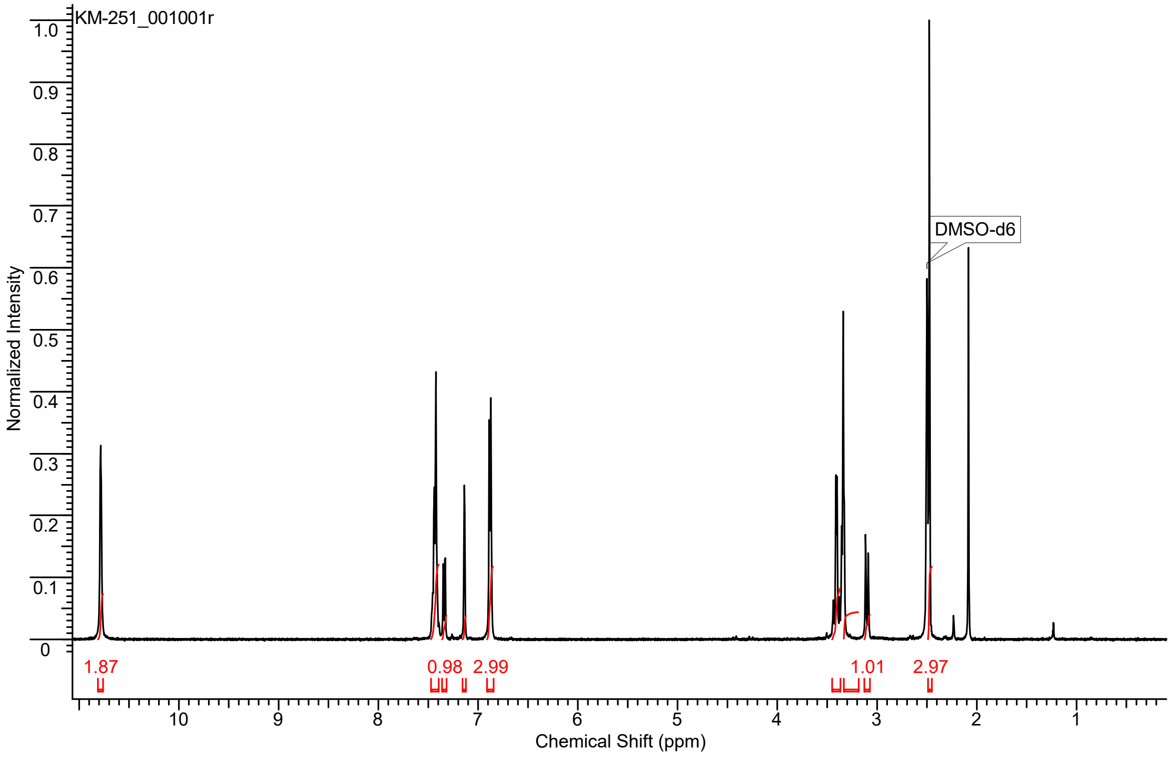

(Z)-5-Chloro-3-(1-(4-methoxyphenyl)-5-oxo-2-selenoxoimidazolidin-4-ylidene)indolin-2-one (5f)

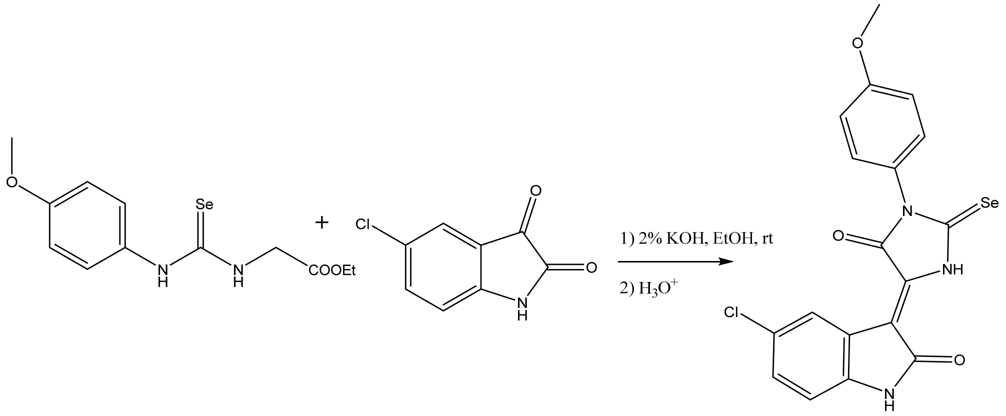

Chemical Formula: $C_{18}H_{12}ClN_3O_3S$
Molecular Weight: 385,8242

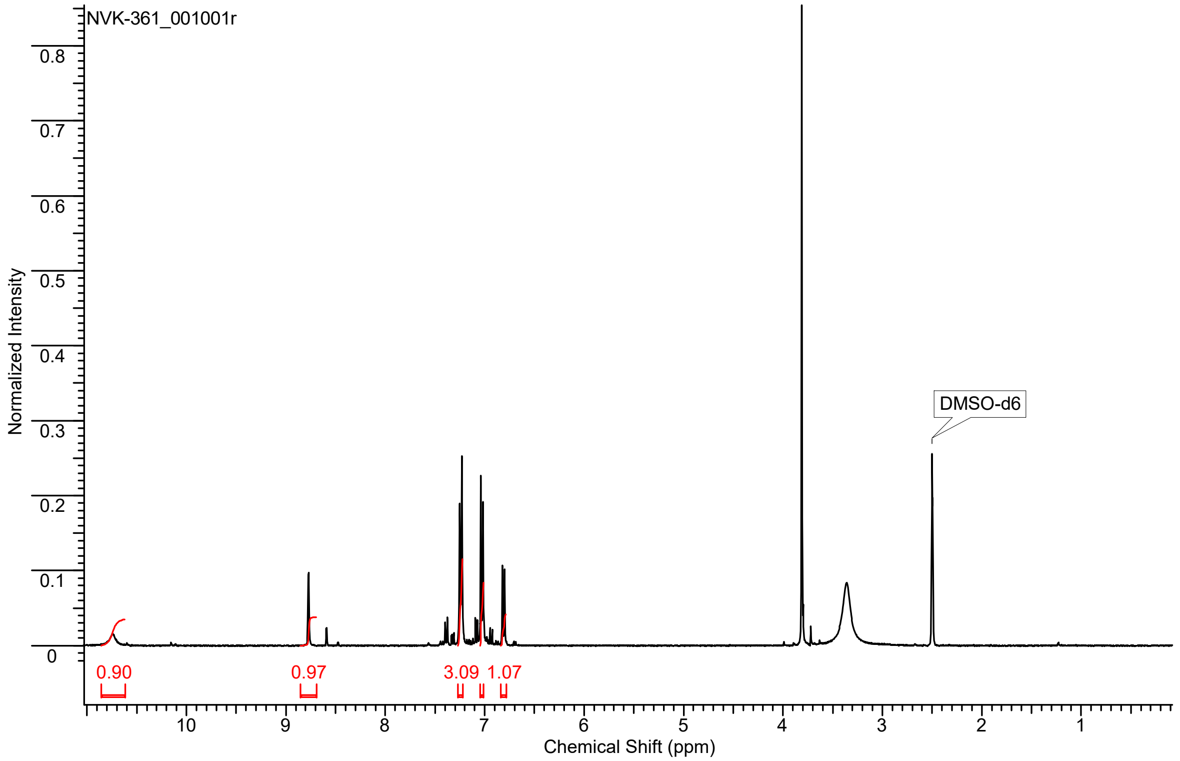

5''-Chloro-1-(3-chloro-4-fluorophenyl)-1'-methyl-2-selenoxodispiro[imidazolidine-4,3'-pyrrolidine-4',3''-indoline]-2'',5-dione (6r)

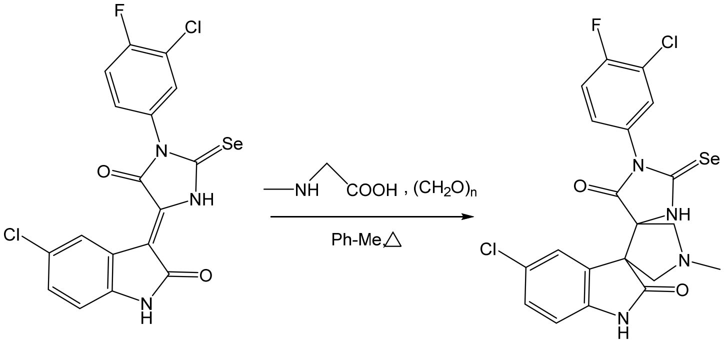

Chemical Formula: $C_{20}H_{15}Cl_2FN_4O_2Se$
Molecular Weight: 512,2244

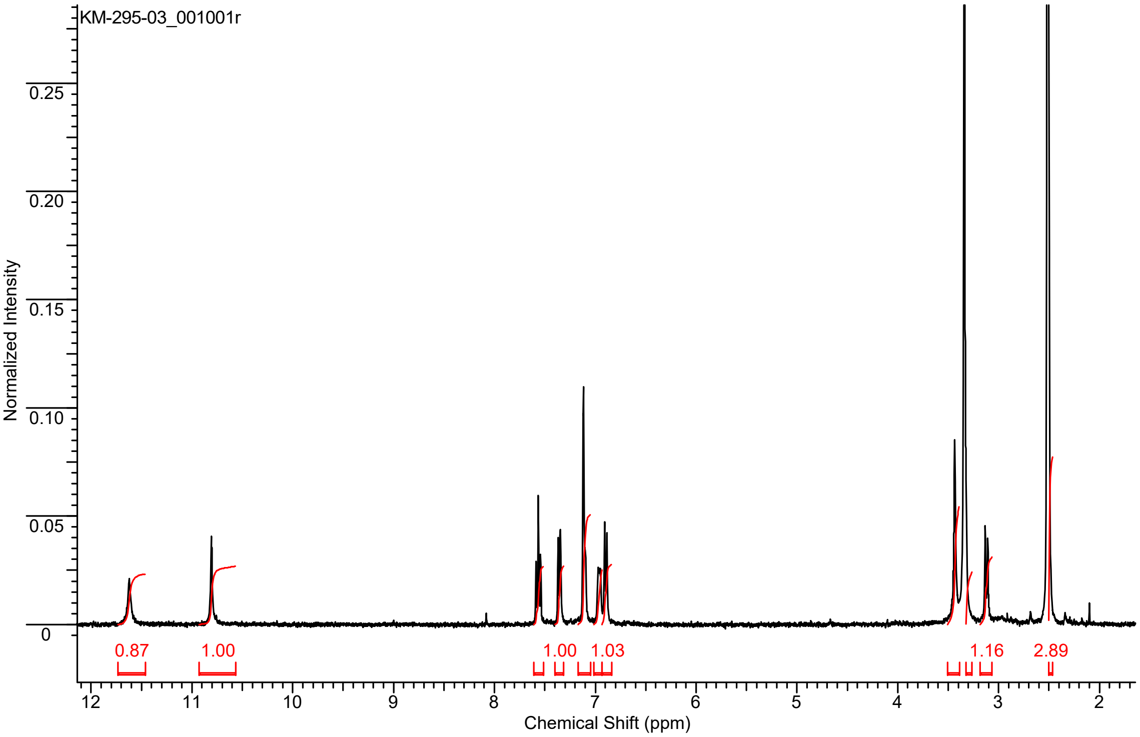

5''-Chloro-1-(4-methoxyphenyl)-1'-methyl-2-selenoxodispiro[imidazolidine-4,3'-pyrrolidine-4',3''-indoline]-2'',5-dione (6f)

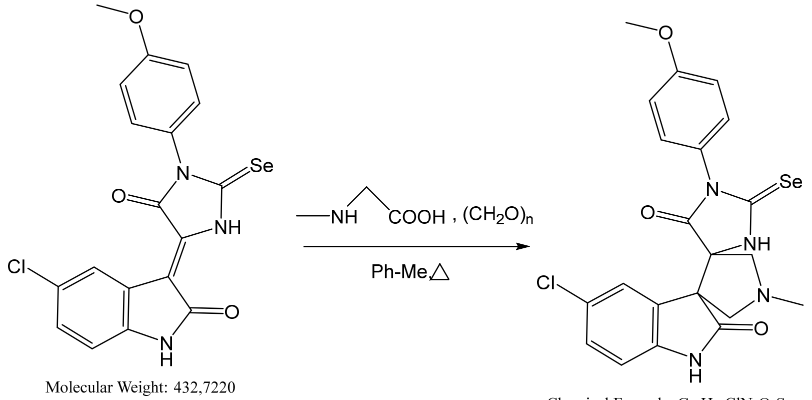

Molecular Weight: 432,7220

Chemical Formula: $C_{21}H_{19}ClN_4O_3Se$
Molecular Weight: 489,8180

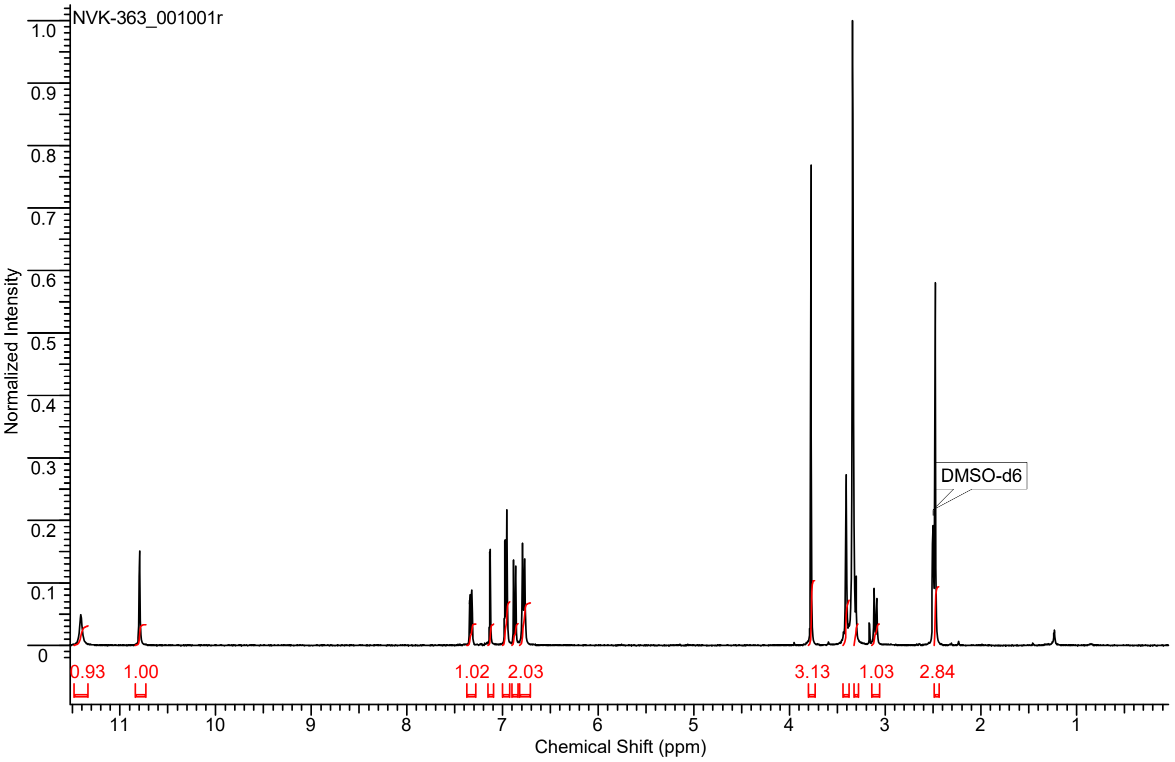

(Z)-5-Chloro-3-(1-(4-methoxyphenyl)-5-oxo-2-thioxoimidazolidin-4-ylidene)indolin-2-one (8f)

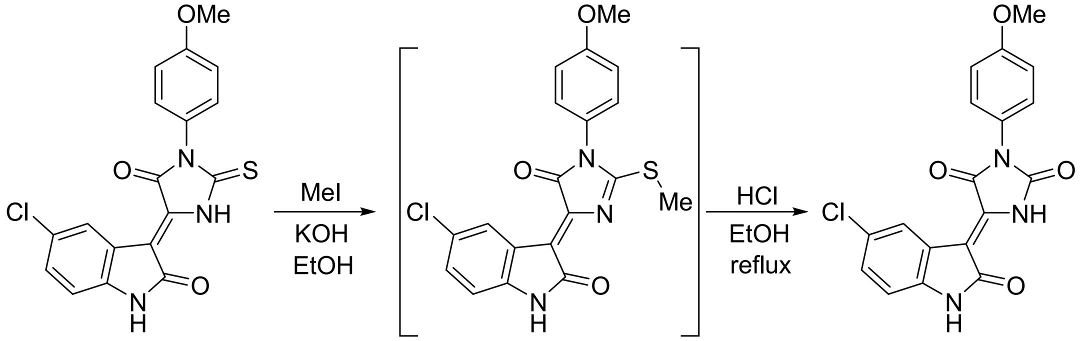

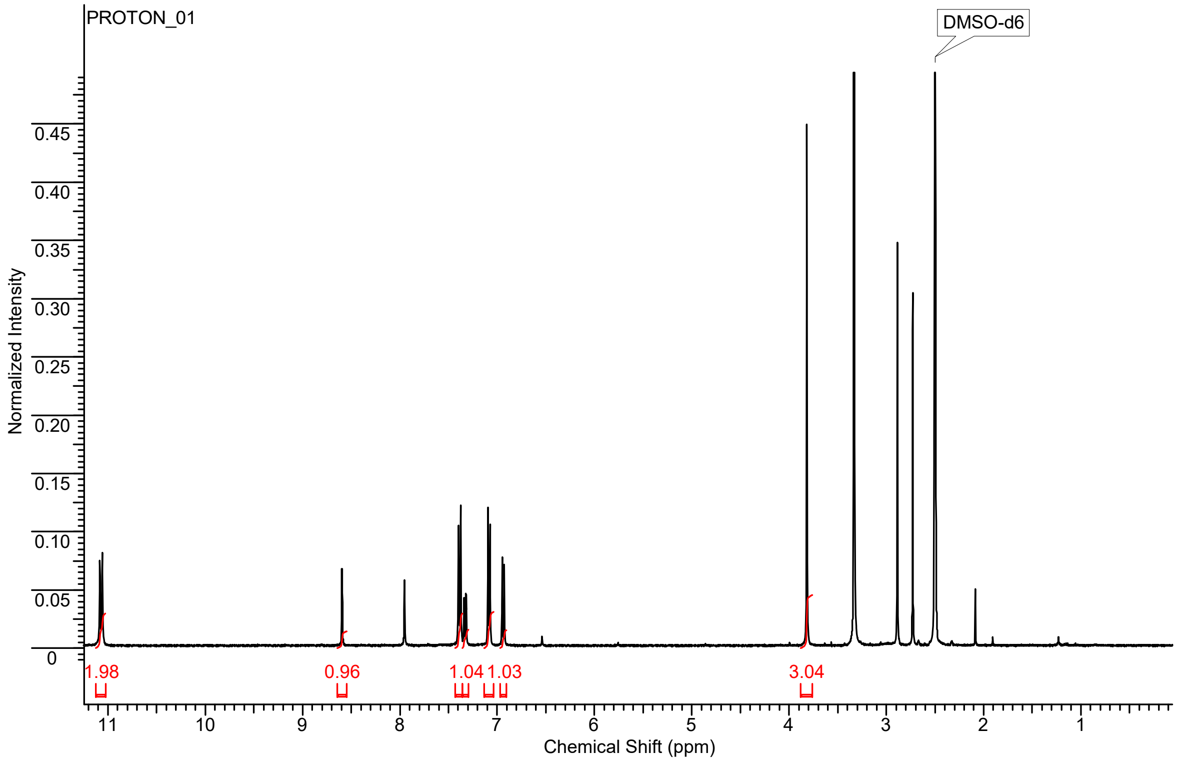

(Z)-5-Chloro-3-(1-(4-ethoxyphenyl)-5-oxo-2-thioxoimidazolidin-4-ylidene)indolin-2-one (8h)

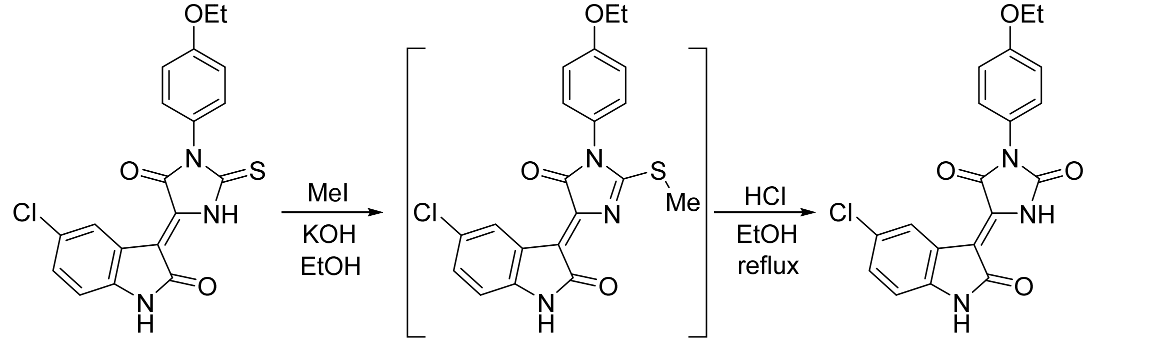

Chemical Formula: $C_{19}H_{14}ClN_3O_4$
Molecular Weight: 383,7880

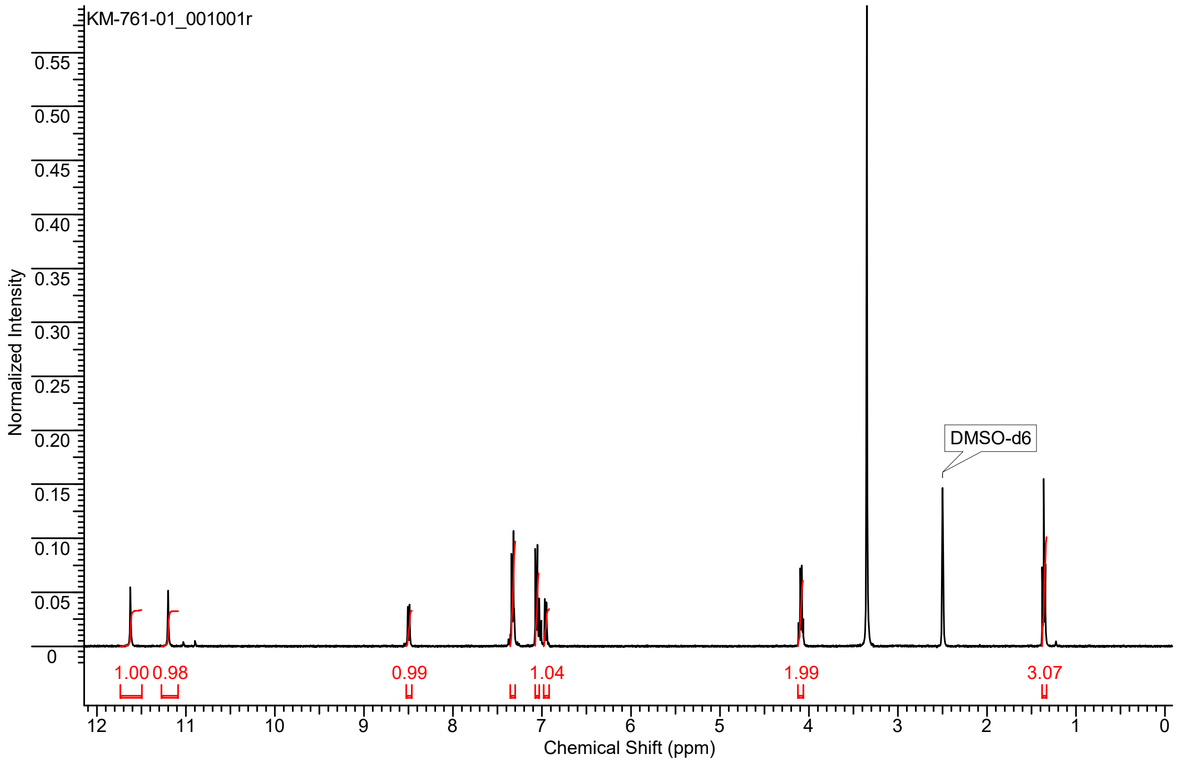

(Z)-5-Chloro-3-(1-(3-chloro-4-fluorophenyl)-5-oxo-2-thioxoimidazolidin-4-ylidene)indolin-2-one (8r)

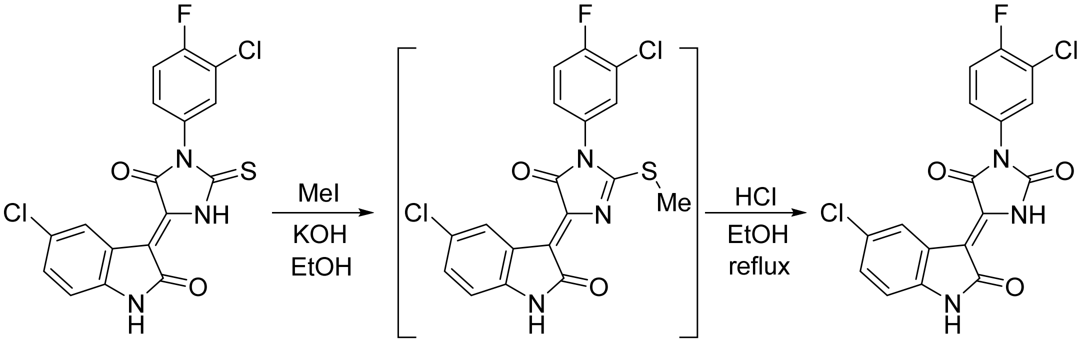

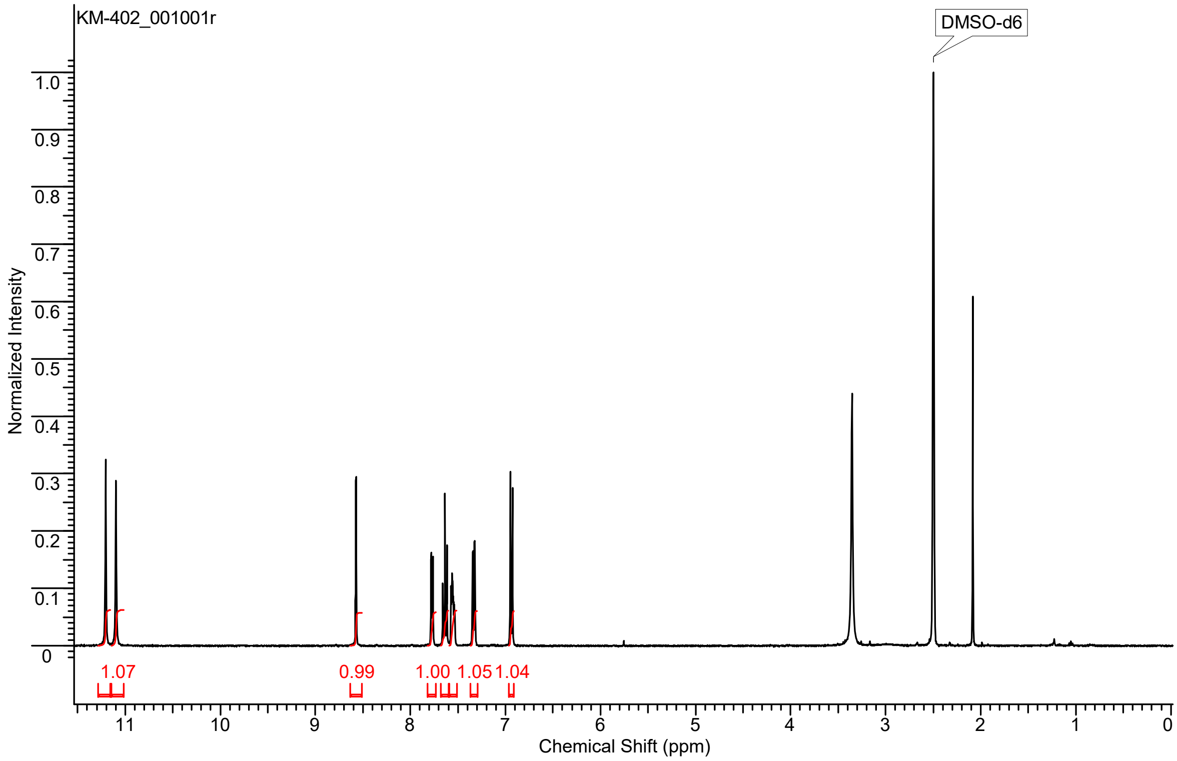

5''-Chloro-1-(4-methoxyphenyl)-1'-methyldispiro[imidazolidine-4,3'-pyrrolidine-4',3''-indoline]-2,2'',5-trione (9f)

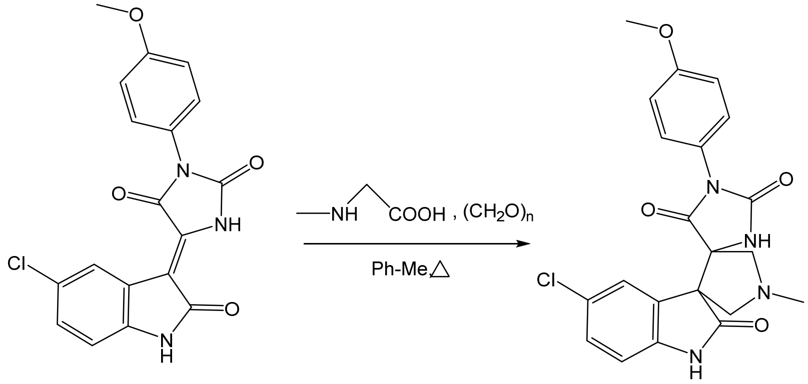

Chemical Formula: $C_{21}H_{19}ClN_4O_4$
Molecular Weight: 426,8570

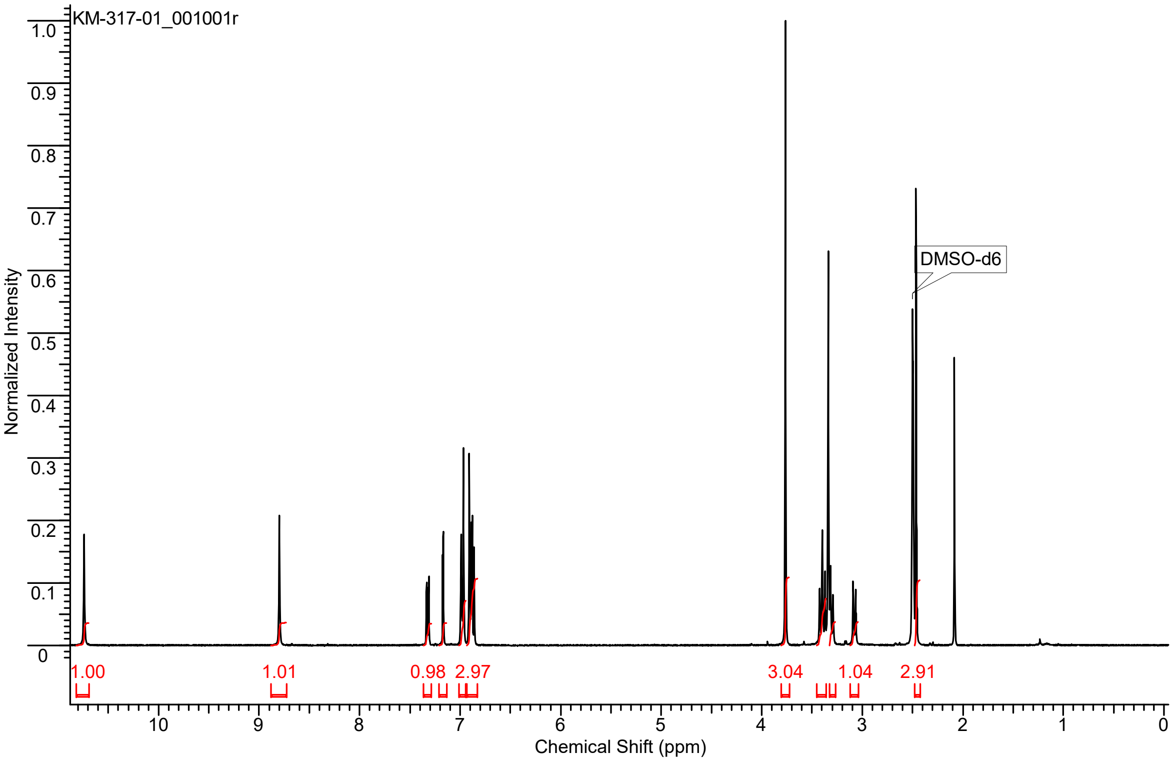

5''-Chloro-1-(4-ethoxyphenyl)-1'-methyldispiro[imidazolidine-4,3'-pyrrolidine-4',3''-indoline]-2,2'',5-trione (9f)

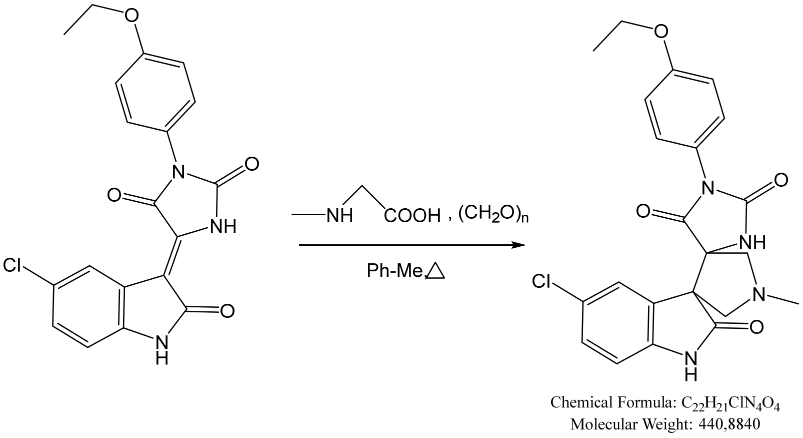

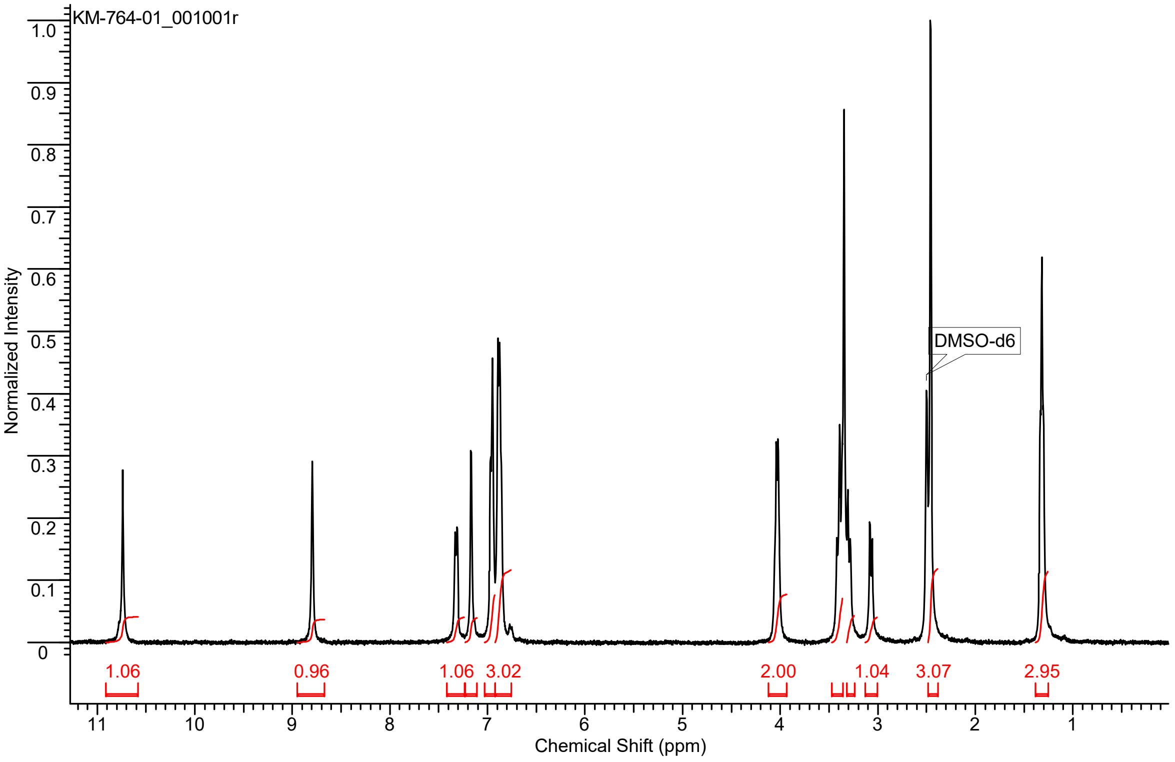

5''-Chloro-1-(3-chloro-4-fluorophenyl)-1'-methyldispiro[imidazolidine-4,3'-pyrrolidine-4',3''-indoline]-2,2'',5-trione (9r)

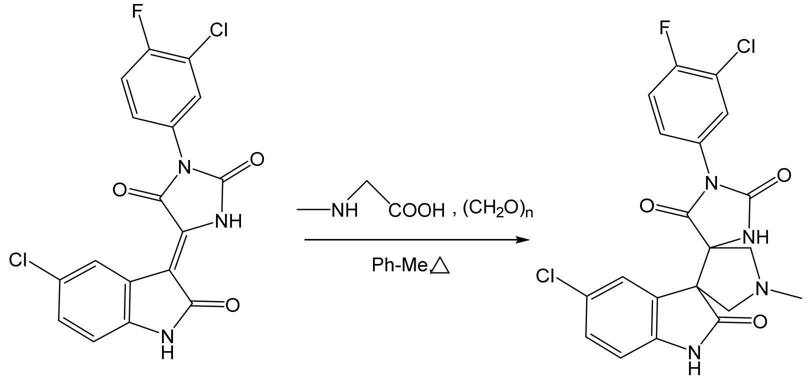

Chemical Formula: $C_{20}H_{15}Cl_2FN_4O_3$
Molecular Weight: 449,2634

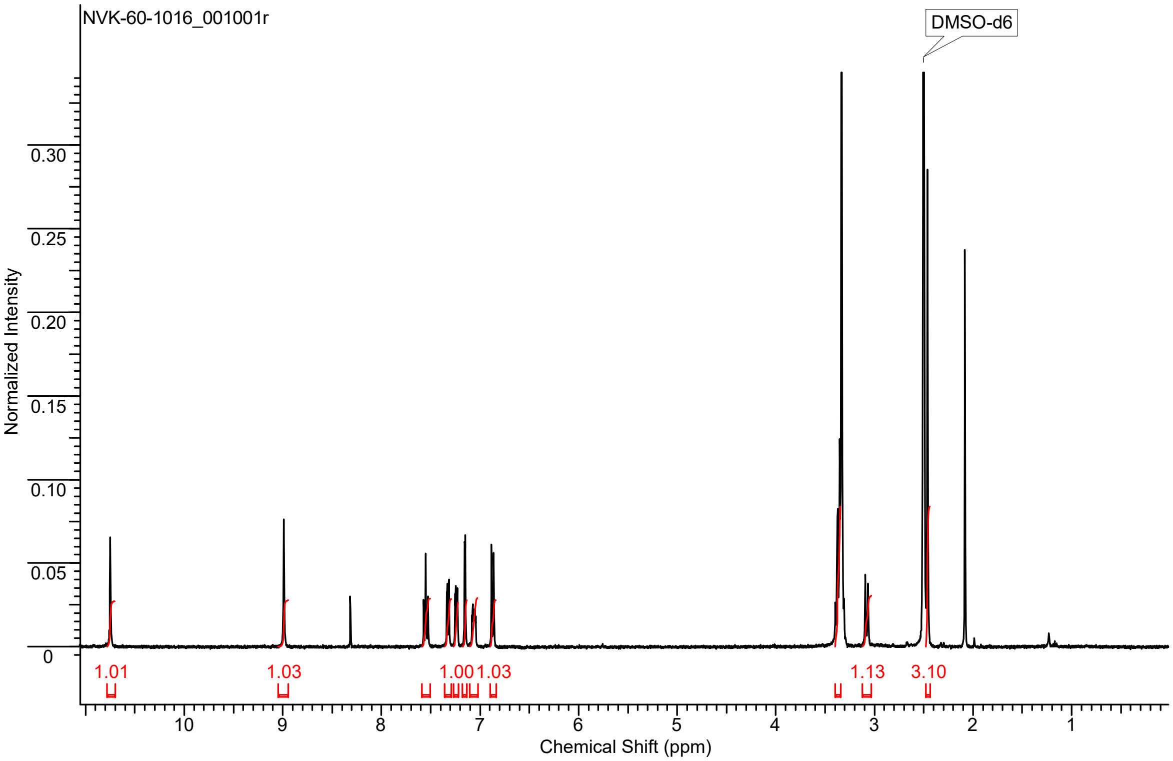

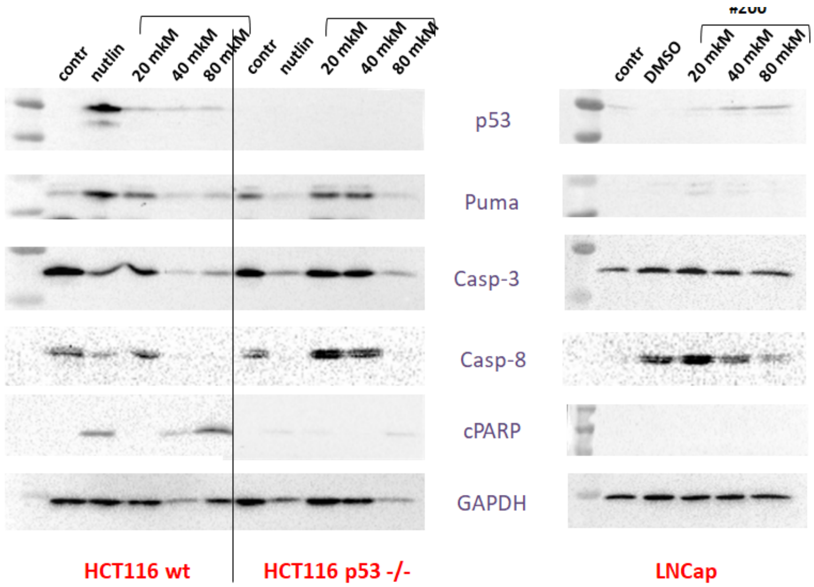

Figure S1. The results of a western blotting study of one of the previously synthesized dispiroindolinones (see chemical formula below) on cancer cell lines which are expressing (HCT116 wt, LNCap) and not expressing (HCT116 p53-/-) p53 protein [Ivanenkov, Y.A.; Vasilevski, S.V.; Beloglazkina, E.K.; Kukushkin, M.E.; Machulkin, A.E.; Veselov, M.S.; Chufarova, N.V.; Vanzcool, A.; Zyk, N.V.; Skvortsov, D.A.; Khutornenko, A.A.; Rusanov, A.L.; Tonevitsky, A.G.; Dontsova, O.A; Majouga, A.G. Design, Synthesis and Biological Evaluation of Novel Potent MDM2/p53 Small-Molecule Inhibitors. *Bioorg. Med. Chem. Lett.* **2015**, *25*, 404–409. DOI: 10.1016/j.bmcl.2014.09.070].

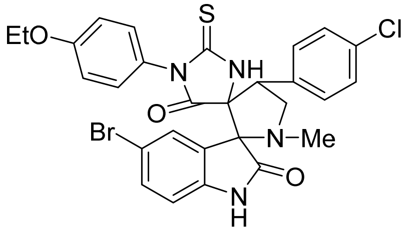

Supplement: Supplementary file 1 [file molecules-26-07645-s001.zip › molecules-1495483-supplementary.pdf]
